# Biotechnological and pharmaceutical potential of twenty-eight novel type strains of *Actinomycetes* from different environments worldwide

**DOI:** 10.1016/j.crmicr.2024.100290

**Published:** 2024-10-11

**Authors:** Imen Nouioui, Judith Boldt, Alina Zimmermann, Roman Makitrynskyy, Gabriele Pötter, Marlen Jando, Meike Döppner, Sarah Kirstein, Meina Neumann-Schaal, Juan Pablo Gomez-Escribano, Ulrich Nübel, Yvonne Mast

**Affiliations:** a Leibniz-Institut DSMZ – German Collection of Microorganisms and Cell Cultures, Inhoffenstraße 7B, 38124 Braunschweig, Germany; b German Center for Infection Research (DZIF), Braunschweig, Germany; c Braunschweig Integrated Centre of Systems Biology (BRICS), Rebenring 56, 38106 Braunschweig, Germany; d Technische Universität Braunschweig, Institut für Mikrobiologie, Rebenring 56, 38106 Braunschweig, Germany

**Keywords:** Actinobacteria, Systematics, Drug discovery, Antibiotics

## Abstract

Actinomycetes are a prolific source of bioactive natural compounds many of which are used as antibiotics or other drugs. In this study we investigated the genomic and biochemical diversity of 32 actinobacterial strains that had been deposited at the DSMZ–German Collection of Microorganisms and Cell Cultures decades ago. Genome-based phylogeny and *in silico* DNA-DNA hybridization supported the assignment of these strains to 26 novel species and two novel subspecies and a reclassification of a *Streptomyces* species. These results were consistent with the biochemical, enzymatic, and chemotaxonomic features of the strains. Most of the strains showed antimicrobial activities against a range of Gram-positive and Gram-negative bacteria, and against yeast. Genomic analysis revealed the presence of numerous unique biosynthetic gene clusters (BGCs) encoding for potential novel antibiotic and anti-cancer compounds. Strains DSM 41636^T^ and DSM 61640^T^ produced the antibiotic compounds A33853 and SF2768, respectively. Overall, this reflects the significant pharmaceutical and biotechnological potential of the proposed novel type strains and underlines the role of prokaryotic systematics for drug discovery. In order to compensate for the gender gap in naming prokaryotic species, we propose the eponyms for all newly described species to honour female scientists.

## Highlights

• Twenty-eight novel actinobacterial type strains including 23 *Streptomyces* species.
• Proposed species names in honour of female scientists.
• Most of the studied strains showed antimicrobial activities against a range of Gram-positive and Gram-negative bacteria, and yeast.
• Presence of numerous unique biosynthetic gene clusters (BGCs) encoding for potential novel bioactive compounds.

## Graphical abstract

**Figure:**
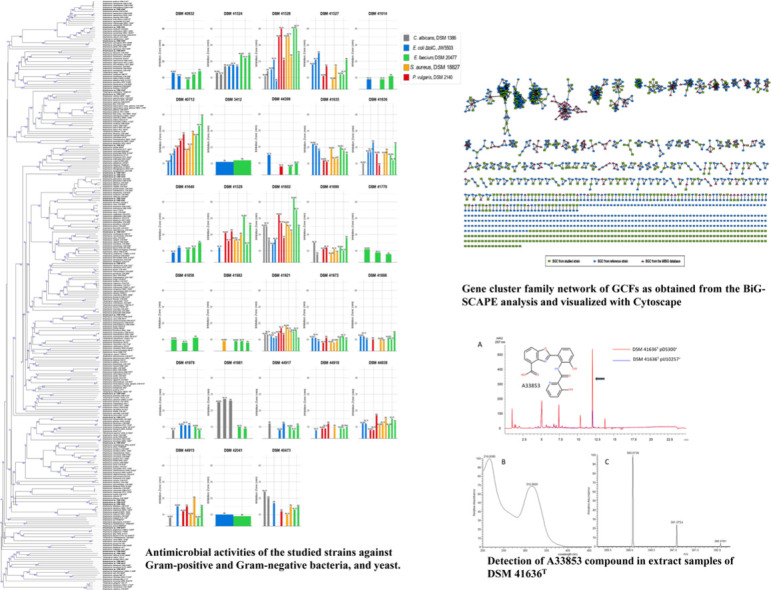

## Introduction

The phylum *Actinomycetota* encompasses Gram-positive bacteria with a high G+C DNA content, grouped into six classes and 35 orders (https://lpsn.dsmz.de/). Strains of this phylum have different lifestyles, such as saprophytes, pathogens, opportunistic pathogens, symbionts, gastrointestinal and plant commensals etc., and are known for their wide range of microscopic forms, including rods and coccoid forms as well as branched filamentous mycelium that may separate to form spores. The latter are special cellular structures known for their resistance to various factors such as heat, desiccation and radiation (Bobek et al., 2017). *Actinomycetota* are widely distributed across diverse habitats, including terrestrial and aquatic ecosystems, as well as extreme environments from hot deserts to Arctic deep seas (Goodfellow et al., 2018). Thereby, the organisms are adapted to a wide range of different physico-chemical parameters, which shape their habitats, including climatic conditions, pH, temperature, moisture etc. (Mohammadipanah and Wink, 2015, Xie and Pathom-Aree, 2021).

The assignment of actinobacterial taxa was based on different phenotypic and genetic approaches that relied heavily on morphological, physiological, and biochemical properties in the early descriptions (Waksman and Curtis, 1916, Bergey et al., 1923, Sneath and Sokal, 1962, Sokal, 1966, Sneath, 1973, Stanier and Van Niel, 1941, Niel, 1946, Stanier and Niel, 1962) Since then, the prokaryotic systematics has evolved significantly to include polyphasic taxonomy, which is based on a combination of phenotypic (including chemotaxonomy), genomic (DNA:DNA hybridization), and genetic analyses (Vandamme et al., 1996, Kämpfer, 2012, Hugenholtz et al., 2021, Nouioui and Sangal, 2022). The latter was mainly based on the 16S rRNA gene, which has been considered as the gold standard marker used for comparative phylogenetic studies of bacteria. However, the resolution of the 16S rRNA gene for some taxa is limited and often not sufficient to distinguish closely related species (Labeda et al., 2012, Nouioui et al., 2018). Alternatively, multi-locus sequence analysis (MLSA), has emerged offering intermediate phylogenetic resolution (Thompson et al., 2005, Gevers et al., 2005). This approach involves phylogenetic comparisons based on a set of housekeeping genes and has clarified the phylogenetic relatedness of several complex taxa such as *Streptomyces* (Rong and Huang, 2014, Labeda et al., 2014, Labeda et al., 2017). Nevertheless, MLSA has shown certain limitations associated with the suitability and relevance of housekeeping genes selected on the basis of their degree of polymorphism (Hugenholtz et al., 2021). These constraints have been addressed by full-length genome sequence comparisons, which has become efficient and affordable over the last decade in the course of the progress of next generation sequencing technology and provide the most accurate phylogenetic resolution (Nouioui et al., 2018).

The systematics of the phylum *Actinomycetota* has undergone several changes over time due to advances in molecular biology and computational genomics. The techniques of DNA-DNA hybridisation (DDH) and DNA G+C content analysis, used since the 1960s for prokaryotic species delineation, had shifted from laborious and time-consuming wet-lab work to digital and more accurate data that have adjusted the taxonomic status of many misclassified taxa due to errors related to the sensitivity of the experiments (JP Meier-Kolthoff et al., 2013, JP Meier-Kolthoff et al., 2014). Other taxogenomic approaches, such as average nucleotide identity (ANI) and average amino acid identity (AAI) have been developed with cut-off values of 95–96% for species demarcation, which has further improved prokaryotic systematics (Goris et al., 2007, Richter and Rosselló-Móra, 2009). Given all these technological advances and the user-friendliness of taxonomic software and web servers (eg. GGDC (https://ggdc.dsmz.de/; (Auch et al., 2010)), the systematics of prokaryotes has been modernised by embracing the whole genome analysis (Hugenholtz et al., 2021, Nouioui and Sangal, 2022). The application of modern technology for bacterial systematic classification allowed to resolve the taxonomic status of several heterogenous complex taxa and helped to distinguish between closely related organisms leading to the reclassification and emendation of many orders, families, genera, species and subspecies. (Nouioui et al., 2018, Hahnke et al., 2016, Carro et al., 2018).

Strains of the phylum *Actinomycetota* are of agricultural, biotechnological, ecological, and medicinal interest (Goodfellow, 2012, Demain, 2014). Two-thirds of all clinically relevant antibiotics, as well as many anticancer, antifungal and immunosuppressive agents are derived from actinomycetes (Bérdy, 2012). This heterogenous group of actinobacterial organisms is the most promising source of new drugs and even though exploited for decades, actinomycetes still hold a huge genetic potential for the production of novel bioactive natural compounds (Gavriilidou et al., 2022). Thereby, natural products (NPs) and semi-synthetic substances derived from them still represent the majority of leads for drug developments compared to compounds obtained by combinatorial biosynthesis or synthetic biology approaches (Newman, 2008) the latter of which represent more modern technologies that still rely on a fundamental understanding of natural compounds biosynthetic pathways. According to the World Health Organisation (WHO) (WHO 2022) the discovery and production of new antibiotics by the pharmaceutical industry has been hampered by lengthy approval procedures, high costs and low success rates. Mining rare, understudied or novel actinomycetes species has proven to be a promising strategy for novel drug discovery (Bauman et al., 2021). The choice of the biological material can be crucial for the success rate of finding new NPs (Leopold-Messer et al., 2023) with especially new sources and/or biology that lead to new chemical entities (Goodfellow et al., 2018 Aug, Goodfellow and Fiedler, 2010).

The genus *Streptomyces* has been extensively studied, resulting in several taxonomic reclassifications and amendments due to advances in the taxonomic approach (Labeda et al., 2012, Labeda et al., 2014, Labeda et al., 2017, Witt and Stackebrandt, 1990, Pridham et al., 1965, Szabó and Marton, 1964). Strains of this taxon are known as producers of secondary metabolites such as antibiotics, anticancer, and antiviral bioactive compounds (Sanjivkumar et al., 2016, Sanjivkumar et al., 2018). However, several actinobacterial taxa are still understudied and little is known about their biotechnological potential in terms of bioactive compounds production. The following non-*Streptomycetaceae* genera are also the focus of this study: *Nocardiopsis, Streptomonospora, Pseudonocardia, Blastococcus,* and *Jatrophihabitans.*

Representatives of all these genera are present in the DSMZ strain collection. The German culture collection DSMZ was founded in 1969 as the national centre for culture collection in Germany and is renowned for its long-standing commitment to microbial taxonomic studies. It is nowadays one of the largest and most diverse culture collections worldwide, currently comprising more than 85.000 bioresources in total, including about 38.000 different bacterial strains. The bacterial diversity covers 80% of all microbial type strains, which come from more than 90 countries. The actinobacteria sub-collection contains more than 6.000 actinobacterial strains including many rare taxa, such as *Actinoalloteichus, Kibdelosporangium, Tsukamurella*, or *Jiangella,* as well as strains that are difficult to handle and/or to maintain, such as *Frankia*. Moreover, the culture collection contains ∼2.500 streptomycetes, many of which have been genome sequenced as part of the Genomic Encyclopedia of Bacteria and Archaea (GEBA) initiative (Seshadri et al., 2022).

In view of the continued emergence of drug-resistant bacteria associated with life-threatening infections, there is an urgent need for new antibiotics, and natural products from taxonomically unique actinobacterial strains are a promising source for novel antimicrobials. In this respect, a screening campaign for new species among a large number of actinobacterial strains deposited at the DSMZ decades ago, has been started focusing on the genus *Streptomyces*, which is the leading producer of antibiotics (Nouioui et al., 2024).

In this study, poorly characterized strains of *Streptomyces, Nocardiopsis, Streptomonospora, Blastococcus, Pseudonocardia,* and *Jatrophihabitans*, were subjected to polyphasic taxonomic characterisation including whole genome sequence analysis. The biotechnological potential of the strains was evaluated based on genome mining approaches and an antimicrobial activity screening. In total, 26 novel species, and two novel subspecies were identified and described and a reclassification of a *Streptomyces* species, which is so far the largest all-at-once description of that many actinobacterial strains. In order to compensate for the gender gap in names of prokaryotes honouring persons (Freese et al., 2023), we propose new taxon names to all newly described species to honour female scientists: *Streptomyces hazeniae* sp. nov., *Streptomyces boetiae* sp. nov., *Streptomyces bugieae* sp. nov., *Streptomyces chisholmiae* sp. nov., *Streptomyces doebereineriae* sp. nov., *Streptomyces doudnae* sp. nov., *Streptomyces millisiae* sp. nov., *Streptomyces dubilierae* sp. nov., *Streptomyces edwardsiae* sp. nov., *Streptomyces evansiae* sp. nov., *Streptomyces gibsoniae* sp. nov., *Streptomyces gottesmaniae* sp. nov., *Streptomyces hesseae* sp. nov., *Streptomyces hintoniae* sp. nov., *Streptomyces mooreae* sp. nov., *Streptomyces johnsoniae* sp. nov.*, Streptomyces litchfieldiae* sp. nov., *Streptomyces lonegramiae* sp. nov., *Streptomyces salyersiae* sp. nov., *Streptomyces lancefieldiae* sp. nov., *Streptomyces stephensoniae* sp. nov., *Blastococcus goldschmidtiae* sp. nov., *Jatrophihabitans lederbergiae* sp. nov., *Nocardiopsis lambiniae* sp. nov., *Pseudonocardia charpentierae* sp. nov., and *Streptomonospora wellingtoniae* sp. nov. The diversity of the biosynthetic gene cluster families of the strains was comprehensively mapped and revealed the abundance of unique BGCs with the potential to encode novel secondary metabolites. For two of the strains, DSM 41636^T^ and DSM 61640^T^, antibiotic substances (A33853 and SF2768, respectively) have been identified.

## Materials and methods

### Bacterial strains, source, maintenance and cultivation

Thirty-two actinobacterial strains were isolated from environmental samples (soil, marine sediments, water, plants, wasp) collected from different geographical locations worldwide and deposited at the DSMZ open culture collection years ago (Table 1). Strains were classified at genus level as *Streptomyces* sp*.* (n=27), *Blastococcus* sp. (n=1), *Jatrophihabitans* sp. (n=1), *Nocardiopsis* sp. (n=1), *Pseudonocardiopsis* sp. (n=1), and *Streptomonospora* sp. (n=1), based on their phenotypic and genetic data. All the reference strains used in this study are available in the DSMZ online catalogue (https://www.dsmz.de/collection/catalogue). Freeze-dried cells were used for chemotaxonomic analysis, with the exception of fatty acids analysis, for which fresh biomass was used. The growth conditions of the strains are listed in the DSMZ catalogue (https://www.dsmz.de/collection/catalogue).

**Table 1:** Origin of the studied strains.

The DSMZ strains	Habitat	Country	Strain designation and culture collection numbers	Proposed species name
		***Streptomyces* strains**		
DSM 3412^T^	soil	Tunisia	Tü 2253^T^, KCTC 59181^T^	*Streptomyces gottesmaniae* sp. nov.
DSM 40473^T^	soil	Unknown country	ATCC 12568^T^, BA-3572^T^, CBS 695.72^T^, IFO 13394^T^, IFO 13907^T^, ISP 5473^T^, KCC S-0694^T^, KCC S-0812^T^, NBRC 13394^T^, NBRC 13907^T^, RIA 1355^T^	*Streptomyces hesseae* sp. nov.
DSM 40712^T^	–	Unknown country	ETH 21066^T^, NRRL 2835^T^, Tü 41^T^	*Streptomyces lancefieldiae* sp. nov.
DSM 40932^T^	–	Unknown country	ATCC 13741^T^, ATCC 13793^T^, CBS 372.58^T^, ETH 24437^T^, NRRL B-1354^T^	*Streptomyces stephensoniae* sp. nov.
DSM 41014^T^	*Sphagnum* pots	Unknown country	IMRU 3065^T^, KCTC 59176^T^	*Streptomyces hintoniae* sp. nov.
DSM 41524^T^	soil	Unknown country	A10598^T^, ATCC 15166^T^	*Streptomyces asiaticus* subsp *ignotus* subsp. nov.
DSM 41527^T^	–	Unknown country	ATCC 21705^T^, SF-1293^T^	*Streptomyces mooreae sp*. nov.
DSM 41528^T^	–	Unknown country	ATCC 21722^T^, FERM-P 602^T^, SF-1084^T^	*Streptomyces bugieae* sp. nov.
DSM 41529^T^	soil	Japan	A-130^T^, ATCC 21840^T^, FERM-P 639^T^	*Streptomyces lonegramiae* sp. nov.
DSM 41602	–	Unknown country	NRRL B-16257, U3S-25	*Streptomyces antimycoticus* subsp. *sporoclivatus* subsp. nov.
DSM 41635	rose root (rhizoplane, *Rosa laxa*)	Germany	5-A4	*Streptomyces edwardsiae* sp. nov.
DSM 41636^T^	rose root (rhizoplane, *Rosa laxa*)	Germany	31-A2^T^, KCTC 59179^T^	*Streptomyces edwardsiae* sp. nov.
DSM 41640^T^	rose root (rhizoplane, *Rosa laxa*)	Germany	13-A30^T^, KCTC 59177^T^	*Streptomyces doebereineriae* sp. nov.
DSM 41699^T^	soil sample from Tasek Bera	Malaysia	ATB-26^T^, KCTC 59183^T^	*Streptomyces gibsoniae* sp. nov.
DSM 41770^T^	water damaged gypsum liner in a children`s day care center	Finland	157/96^T^, KCTC 59182^T^	*Streptomyces salyersiae* sp. nov.
DSM 41859	agar plate culture of *Aureobasidium pullulans*	Germany	Agent 'Z'	*Streptomyces evansiae* sp. nov.
DSM 41886^T^	marine sediment	USA	CNB 984^T^, KCTC 59171^T^	*Streptomyces johnsoniae* sp. nov.
DSM 41921^T^	rhizospherical soil of resistant *Vitis vinifera*	Morocco	S6*,* KCTC 59180^T^	*Streptomyces dubilierae* sp. nov.
DSM 41972^T^	Atta colombica refuse dump	Panama	Av26–2^T^, DI-188^T^, KCTC 59178^T^	*Streptomyces althioticus* subsp. *attaecolombicae* subsp. nov.
DSM 41979^T^	*sirex noctilio*	USA	SA3-ActF^T^, KCTC 59185^T^	*Streptomyces evansiae* sp. nov.
DSM 41981^T^	solitary wasp	Panama	Sol5a-2^T^, KCTC 59175^T^	*Streptomyces doudnae* sp. nov.
DSM 41982	solitary wasp	Panama	Sol7th	*Streptomyces evansiae* sp. nov.
DSM 42041^T^	gorgonian coral	China	SCSIO 10374^T^, KCTC 59186^T^	*Streptomyces hazeniae* sp. nov.
DSM 44915^T^	marine sediment	Palau	SCRIPP CNJ 962^T^, KCTC 59194^T^	*Streptomyces chisholmiae* sp. nov.
DSM 44917^T^	marine sediment	USA	SCRIPP CNQ 259^T^, KCTC 59173^T^	*Streptomyces boetiae* sp. nov.
DSM 44918^T^	marine sediment	Guam / USA	SCRIPP CNQ 703^T^, KCTC 59174^T^	*Streptomyces millisiae* sp. nov.
DSM 44938^T^	marine sediment	Bahamas	SCRIPP CNR 954^T^, KCTC 59172^T^	*Streptomyces litchfieldiae* sp. nov.
		**Actinobacterial strains**		
*Blastococcus* sp. DSM 46792^T^	marble	Italy	7C^T^, BC543^T^, KCTC 59190^T^	*Blastococcus goldschmidtiae* sp. nov.
*Nocardiopsis* sp. DSM 44743^T^	soil	China	DCDM15A35^T^, KCTC 59192^T^	*Nocardiopsis lambiniae* sp. nov.
*Pseudonocardia* sp. DSM 45834^T^	desert sand sample	China	CPCC 203558^T^, KCTC 59187^T^	*Pseudonocardia charpentierae* sp. nov.
*Streptomonospora* sp. DSM 45055^T^	marine sediment	USA	CNQ 327^T^, KCTC 59191^T^	*Streptomonospora wellingtoniae* sp. nov.
*Jatrophihabitans* sp. DSM 44399^T^	sandstone of Linnaeus Terrace (1600 m)	Antarctica	IFAM AA-499^T^, AA-499^T^	*Jatrophihabitans lederbergiae* sp. nov.

### Previously reported antibiotic production profile of the studied strains

Based on previous studies, six out of 27 *Streptomyces* strains are known for their ability to produce antibiotics, antifungals, and enzymes. Strains DSM 41524^T^, DSM 41527^T^, DSM 41528^T^, and DSM 41529^T^ were deposited at the DSMZ collection on 19.05.1989 under the species name S. *hygroscopicus* subsp. *hygroscopicus,* while strain DSM 41602 was initially assigned to *Streptomyces violaceusniger* species*.* Strains DSM 41529^T^, DSM 41527^T^, DSM 41528^T^ were known as producers of antibiotic A-130 (Oikawa et al., 1975), herbicide bialaphos (Murakami et al., 1986)*,* and amylase (Koaze et al., 1974), respectively. Strains DSM 40932^T^ and DSM 40712^T^, originally affiliated to *Streptomyces griseus* subsp. *farinosus* and *Streptomyces albogriseolus*, respectively. These strains were found to synthesize streptolins A, B, streptothricin, vitamin B12 and chalcomycin and echinomycin, respectively. The assignment of strains DSM 41524^T^, DSM 41527^T^, DSM 41528^T^, DSM 40932^T^, DSM 40712^T^, and DSM 41602^T^ to the species listed above was based on numerical classification (Kämpfer et al., 1991). Strains DSM 41640^T^ and DSM 3412^T^, deposited at the DSMZ culture collection between 1985–1994, were originally described as *Streptomyces violaceusniger* and *Streptomyces galbus* subsp. *eurythermus,* respectively*.* Strain DSM 3412^T^ was known to produce antifungal macrolides called galbonolides A and B (Achenbach et al., 1988). Strain DSM 40473^T^ was previously known as *Streptoverticillium parvisporogenum* (Locci, 1969), basonym of *Streptomyces parvisporogenes* that has been considered as a heterotypic synonym to *Streptomyces abikoensis* (Umezawa et al., 1951) Witt and Stackebrandt 1990. For this reason, this strain appears in different culture collections as *Streptomyces abikoensis.* Strain DSM 40473^T^ was earlier described as the producer of the antibiotic PA-150 (Koe et al., 1957, English and McBride, 1957).

Strains DSM 41635 and DSM 41636^T^, and DSM 41770^T^ were formerly assigned to *Streptomyces griseoflavus* and *Streptomyces griseus,* respectively*.* Strains DSM 41014^T^, DSM 41981^T^, DSM 41921^T^, DSM 41699 ^T^, DSM 41982^T^, DSM 41859^T^, DSM 41972^T^, DSM 42041^T^, DSM 44917^T^, DSM 44915^T^, DSM 44938^T^, DSM 44918^T^, and DSM 41979^T^ were accessed at the DSMZ between 1980 and 2011 as *Streptomyces* sp. Strain DSM 41886^T^ was deposited in 2006 as ‘*Marinispora* sp.’; the genus *Marinispora* of the family *Streptomycetaceae* has never been validated. All the non-*Streptomyces* strains (*Nocardiopsis* sp. DSM 44743^T^, *Blastococcus* sp. DSM 46792^T^, *Streptomonospora* sp. DSM 45055^T^, *Pseudonocardia* sp. DSM 45834^T^) included in this study were deposited at the German collection between 2003–2013. All strain designations and culture accession numbers of the studied strains are provided in Table 1 and in the protologue for species description. No previous studies on the pharmaceutical biotechnological or ecological potential of these strains were performed, with the exception of those mentioned above.

### Growth and morphological properties

The ability of the strains to grow in the presence of the following media was evaluated: International *Streptomyces* Project (ISP), ISP1 (DSMZ 1764), ISP2 (DSMZ 987) ISP3 (DSMZ 84), ISP4 (DSMZ 252), ISP5 (DSMZ 993), ISP6 (DSMZ 1269), ISP7 (DSMZ 1619), GYM (Glucose-Yeast extract-Malt extract = DSMZ 65) (Shirling and Gottlieb 1966), TSA (Trypticase Soy Agar = DSMZ 535), N-Z amine (DSMZ 554), GPHF (DSMZ 553), DSMZ 714, Luedemann (DSMZ 877), Bennett's (DSMZ 548), Gause synthetic media N°1, nutrient agar (DSMZ 1), Czapek peptone (DSMZ 83) agar media. Morphological traits, including the colour of the aerial and substrate mycelia of the strains, were recorded using the RAL colour chart. Moreover, the strains were subjected to a wide range of temperatures (4 °C, 10 °C, 15 °C, 25 °C, 28 °C, 37 °C, 42 °C, and 45 °C) and pH (5.0, 5.5, 6.0, 6.5, 7.0, 7.5, 8.0, 8.5, 9.0) tests. A bacterial suspension of 5 on the McFarland scale was used for inoculation of all duplicated tests (McFARLAND, 1907).

### 16S rRNA gene-based identification and genome sequencing

Genomic DNA was extracted from active cultures of strains grown under optimal growth conditions (https://www.dsmz.de/collection/catalogue). DNA extraction was carried out by the microbial DNA service at the DSMZ as described before (Wright et al., 2023). The bacterial DNA was subjected to PCR-mediated amplification of a 16S rRNA gene and was sequenced using a 96-capillary-system from Applied Biosystems (ABI) as reported previously (Risdian et al., 2021).

For genome sequencing, a fresh biomass of 30–50 mg was harvested from the active culture of the strains and added to 500 µl of DNA/RNA Shield, a lysis buffer provided by the MicrobesNG service. Extraction, purification, quantitative and qualitative estimation of the DNA as well as sequencing on the Illumina platform were performed by MicrobesNG service (https://microbesng.com). 250 bp paired end reads and 30X depth of coverage for Illumina sequencing was used. MicrobesNG bioinformatic pipeline used for a final draft genome sequence includes Kraken (Wood and Salzberg, 2014), a system for taxonomic assignment, a software for mapping the reads, BWA mem (Li and Durbin, 2010, Li, 2013), and assembly programme, SPAdes (Bankevich et al., 2012). The genomes were annotated by NCBI, using the NCBI Prokaryotic Genome Annotation Pipeline (PGAP) (Li et al., 2021). The draft genome sequences of the studied strains have been deposited in GenBank. The genome accession numbers of the studied strains are listed in Table S1.

### Phylogeny and comparative genomic studies

To confirm the authenticity of the strains, an almost complete 16S rRNA gene sequence (>1.400 bp) extracted from the draft genome sequence of the strains was aligned with that obtained from PCR using Basic Local Alignment Search Tool (BLASTN) available on the NCBI web server (https://blast.ncbi.nlm.nih.gov/Blast.cgi) (Zhang et al., 2000, Morgulis et al., 2008). BLAST comparative analysis of the 16S rRNA gene sequence of the strains of interest against those of the type strains of validly named species was carried out via the EZBioCloud server (https://www.ezbiocloud.net/) (Yoon et al., 2017). Maximum likelihood 16S rRNA gene and genome-based phylogenetic trees were constructed using the Type Strain Genome Server [TYGS, (https://tygs.dsmz.de/)] (Meier-Kolthoff and Göker, 2019, Meier-Kolthoff et al., 2022). Comparative genomic analysis for prokaryotic species and subspecies delineation based on digital DNA-DNA hybridization (dDDH) was performed between the genome of the studied strains and their closest phylogenomic neighbours using the recommended formula 4 provided by the TYGS (http://ggdc.dsmz.de) web server (Meier-Kolthoff et al., 2022). Genomic features of the strains and their close phylogenomic neighbours, such as genome size, G+C content, number of coding sequences, number of RNA genes, N50, were retrieved from GenBank.

### Biochemical and chemotaxonomic analyses

The ability of the strains to metabolise a range of carbohydrates was examined using the API 50 CH (according to the manufacturer's instructions, Biomérieux, France). The enzymatic pattern of the strains was determined using the API ZYM kit (according to the manufacturor's instructions, Biomérieux, France). In addition, API 20 NE was used to determine the biochemical feature of *Streptomonospora* sp. DSM 45055^T^, *Nocardiopsis* sp. DSM 44743^T^, *Blastococcus* sp. DSM 46792^T^, *Jatrophihabitans* sp. DSM 44399^T^. Standard thin-layer chromatographic (TLC) procedures were used for analyses of polar lipids (Minnikin et al., 1984, Goodfellow and Kroppenstedt, 2006), isomers of diaminopimelic acid of the peptidoglycan (Schleifer and Kandler, 1972), and whole cell sugars (Lechevalier and Lechevalier, 1979). Fresh biomass of the strains was used for cellular fatty acid analysis. Fatty acids were converted into fatty acid methyl esters (FAMEs) following the protocol of saponification, methylation and extraction of (Sasser, 1990). FAMEs were analyzed by gas chromatography coupled to a flame ionization detector (Agilent instrument, model 6890N). A GCMS– run on an Agilent GC–MS 7000D instrument was used for identification of fatty acids (Vieira et al., 2021). Subsenquently, FAMEs were derivatized to dimethyl disulfide adducts to resolve the position of the single double bonds (Moss and Lambert-Fair, 1989) or to 4,4-dimethyloxazoline (DMOX) derivatives (Spitzer, 1996) to determine branched-chain fatty acid positions and cyclopostions, respectively.

### Biosynthetic gene cluster and cluster similarity network analysis

A total of 76 genome sequences of the studied strains (n = 32) and their related phylogenomic relatives (n = 44) were subjected to antiSMASH analysis version 7.0.1 (Blin et al., 2023), using GenBank annotation files from the assemblies published with NCBI as input and relaxed strictness settings. The results were subsequently separated into all BGC regions, regions within contigs, and regions on contig edges. All sets of BGC regions (all regions, within contigs, on contig edges) were clustered into GCFs with BiG-SCAPE version 1.1.5 (Navarro-Muñoz et al., 2020) using the default cut-off of 0.3, including the MIBiG database, and allowing the mixing of all classes of natural products (–mix). The gene cluster network obtained from BiG-SCAPE was visualized with Cytoscape (version 3.10.2, (Shannon et al., 2003)). The strains were clustered according to their GCF coverage using the python function seaborn.clustermap with default settings (method=’average’, metric=’euclidean’).

### Preparation of culture extracts and antimicrobial bioassay

The strains were cultivated in 50 mL of R5 medium at 28 °C (Nouioui et al., 2024). After three days, 5 mL of preculture were transferred in 50 mL of fresh R5 and NL800 medium, respectively, and the culture was grown for four days at 28 °C in 250 mL Erlenmeyer flasks on an orbital shaker (180 rpm) as described before (Nouioui et al., 2024). In cases where the strains did not grow in R5 or NL800, the optimal growth medium suggested for the strain was used, as listed on the DSMZ online catalogue. Organic compounds were extracted using 5 mL of ethyl acetate (EtAc) as described by Nouioui et al. (2024). Crude extracts were tested against reference strains of Gram-positive (*Staphylococcus aureus* DSM 18,827 (Multiple Antibiotic Resistant strain) and *Enterococcus faecium* DSM 20477**^T^**) and Gram-negative bacteria (*Escherichia coli ΔtolC* JW5503–1, *Proteus vulgaris* DSM 2140), as well as yeast (*Candida albicans* DSM 1386). All reference strains are available in the DSMZ online catalogue along with their respective growth conditions (https://www.dsmz.de/collection/catalogue). Antimicrobial tests were carried out according to Nouioui et al. (2024) using a 30 µl volume of methanolic crude extract applied in wells in agar plates inoculated with the test microorganisms listed above. The diameter of the inhibition zone was measured after incubation of the plates. Due to the large number of samples, only one biological replicate was prepared in each case.

### SARP overexpression in strain DSM 41636^T^ and DSM 61640^T^

To overexpress *papR2* in streptomycetes, a conjugative, φBT1-based integrative construct was generated. The plasmid pIJ10257 (Hong et al., 2005) was used as a vector, which contains the ermE* promoter for the induction of gene transcription, a hygromycin resistance cassette (*hyg*^R^) for selection, and *oriT*, required for the intergeneric conjugation from *E. coli* to *Streptomyces.* The *papR2* gene was PCR amplified from the genomic DNA of *Streptomyces pristinaespiralis* PR11 using a pair of primers RM_papR2_exp_for (AAAAAACATATGAAGTTCCGCATTCTCGGTCC) and RM_papR2_exp_rev (AAAAAAGCTTCTAGTGGCCCGAGGCCG). The obtained 1 kb long DNA fragment was digested with HindIII and NdeI and then cloned into the respective sites of pIJ10257. This resulted in the plasmid pDS300, where *papR2* transcription is under the control of the constitutive erythromycin promoter ermE*. The pDS300 plasmid was transferred to DSM 41636^T^ and DSM 41640^T^ by conjugation, as described in Kieser et al. (2000), resulting in the strain DSM 41636^T^::pDS300 and DSM 61640^T^::pDS300*,* respectively. As a control, strains carrying the empty vector pIJ10257 were used. Hygromycin B (50 µg/mL) was used for selection.

### Antibiotic production conditions for DSM 41636^T^ and DSM 61640^T^ and preparation of culture extracts

The strains DSM 41636^T^ and DSM 41640^T^ were cultivated by inoculation from a GYM plate in 25 mL medium TSB at 28 °C in 250 mL Erlenmeyer flasks on an orbital shaker (180 rpm). After two days of cultivation, 5 mL of the preculture were used to inoculate 500 mL Erlenmeyer flasks with 50 mL of production media R5 or NL 19 as production cultures, that were incubated for four days at 28 °C on an orbital shaker at 180 rpm. For extraction of organic compounds, 20 mL of culture was extracted with 20 mL of ethyl acetate for 3 h at room temperature under constant vertical rotation. After centrifugation at 5.000 rpm for 10 min, the organic phase was completely dried using a centrifugal evaporator (SP Genevac EZ-2, “Low BP” program). The concentrated extracts were dissolved in 0.5 mL methanol (MeOH) and then used for LC-HRMS (liquid chromatography-high resolution mass spectrometry) analysis.

### Chemical analyses for compound detection

Low resolution LC-MS data were recorded on a HPLC Agilent 1260 system connected to a G6125B mass spectrometer with a reversed-phase Poroshell C18 column (Agilent, 2.7 μm, 100 × 3 mm) using water as mobile phase A and acetonitrile as mobile phase B, both containing 0.1% formic acid (vol/vol) as a solvent modifier. Elution was carried out at 0.5 mL/min as follows: 0 min 95% A, 0.5 min 95% A, 18.5 min 5% A, 20.5 min 5% A, 20.8 min 95% A, 25 min 95% A. Full-scan mass spectra (*m/z* 200–2000) were collected in both positive and negative ESI modes. The following parameters were used: capillary voltage, 3000 V; nebulizer gas pressure, 35 psi; drying gas flow rate (N_2_), 10 L/min; drying gas temperature, 350 °C.

Identification of A33853 from DSM 41636^T^: A33853 was identified using a combination of its UV/vis spectrum and LC-HRMS. The UV/vis spectrum of the identified compound is virtually identical to the previously reported spectrum of A33853 (Michel et al., 1984). Its deprotonated molecule = *m/z* 390.0726 [M – H]^–^ (calculated (calcd) for C_20_H_13_N_3_O_6_, 390.0732, Δ −1.54 ppm) yielded a formula (C_20_H_13_N_3_O_6_) matching that of A33853. Identification of SF2768 from DSM 41640^T^: SF2768 was identified using LC-HRMS. Its protonated molecule = *m/z* 337.1865 [M+H]^+^ (calculated (calcd) for C_16_H_24_N_4_O_4_, 337.187, Δ −1.48 ppm) yielded a formula (C_16_H_24_N_4_O_4_) matching that of SF2768.

## Results and discussion

### 16S phylogenetic analysis leads to the identification of new actinobacterial type strains

#### Phylogenetic analysis of Streptomyces strains based on 16S rRNA gene

Molecular identification based on the 16S rRNA gene sequence assign the strains to the class *Actinomycetes* and phylum *Actinomycetota*. Pairwise 16S rRNA gene sequence similarity confirms the affiliation of the strains to their respective genus. The authenticity of the strains was confirmed by comparing the 16S rRNA sequence obtained by PCR with that from the genome sequences of the strains. The 28 *Streptomyces* strains had 16S rRNA gene similarities between 98.2–100% with their closest phylogenetic relatives. The genus *Streptomyces* of the family *Streptomycetaceae* (Waksman and Henrici, 1943) and order *Kitasatosporales* (Kämpfer 2012) comprises more than 700 species with validly published names, with *Streptomyces albus* (Waksman and Henrici, 1943, Rossi, 1891) as the type species. Streptomycetes are present in different ecological niches (soil, sediment, plant, animal, human, etc.), though they predominate in soil. They are known for their saprophytic lifestyle. These filamentous actinomycetes are characterized by a classic sporulating life cycle consisting of spore germination and the development of primary substrate mycelia, followed by the formation of secondary aerial mycelia and production of spores.

A Maximum-likelihood (ML) phylogenetic tree based on 16S rRNA gene sequences showed that most of the *Streptomyces* strains had a distinct phylogenetic position within the evolutionary radiation of the genus *Streptomyces (*Figure S1). The phylogenetic distribution of the studied strains (DSM 41524^T^, DSM 41,602, DSM 41529^T^, DSM 41527^T^, DSM 42041^T^, DSM 41979^T^, DSM 41,982, DSM 41,859, DSM 40712^T^, DSM 41921^T^, DSM 41981^T^, DSM 41014^T^, DSM 41699^T^, DSM 44915^T^) in the ML tree is in concordance with the 16S rRNA gene sequence similarity (Table S2), as they were closely related to their reference strains which have the highest similarity values, unlike strains DSM 40473^T^, DSM 41528^T^, DSM 41,635, DSM 41636^T^, DSM 41770^T^, DSM 40932^T^ DSM 3412^T^ DSM 41640^T^, DSM 41640^T^, DSM 44938^T^, DSM 44917^T^, DSM 41886^T^, DSM 44918^T^ (Figure S1, Table S2). The phylogenetic relationships of the strains were not well supported in the tree.

#### Phylogenetic analysis of non-Streptomyces strains based on 16S rRNA gene

The genera *Nocardiopsis* (Brocq-Rousseau, 1904, Meyer, 1976) and *Streptomonospora* (Cui et al., 2001, Li et al., 2003) belong to the family *Nocardiopsidaceae* (RAINEY et al., 1996, Zhi et al., 2009) and the order *Streptosporangiales* (Goodfellow, 2015), harbouring 47 and 10 validly named species, respectively. The type genus *Pseudonocardia* (Henssen, 1957) of the family *Pseudonocardiaceae* (Embley et al., 1988) and the order *Pseudonocardiales* (Labeda and Goodfellow, 2015) contains more than 65 validly named species. The genera *Blastococcus* (Ahrens and Moll, 1970) and *Jatrophihabitans* (Madhaiyan et al., 2013) of the families *Geodermatophilaceae* (Normand, 2006) and *Jatrophihabitantaceae* (Nouioui et al., 2018) are classified in the orders *Geodermatophilales* (Sen et al., 2014) and *Jatrophihabitantales,* which encompass 14 and 5 validly named species, respectively. The five non-*Streptomyces* strains had 16S rRNA gene similarities between 97.56–99.72% with their closest phylogenetic relatives.

Strains of the genus *Blastococcus,* which has *Blastococcus aggregatus* (Ahrens and Moll, 1970) as the type species, are characterized by the presence of rod and/or cocci-shaped cells that can be aggregated (Urzì et al., 2004, Hezbri et al., 2016). The cells have been shown to be motile rod-shaped cells and /or non-motile cocci (Lee, 2006). This group of microorganisms has been isolated from different habitats, such soil and sand from archaeological sites and deserts, marble, sediments, and medicinal plants (Montero-Calasanz et al., 2022). Strain DSM 46792^T^ had 99.7% 16S rRNA gene similarity with the type strain of *Blastococcus fimeti* species. In the ML phylogenetic tree, the studied strain occupied a distinct branch closely associated with the type strains of *B. fimeti* and *Blastococcus aggregatus* species and next to *Blastococcus aurantiacus* DSM 44268^T^. It is evident from the low resolution of the 16S rRNA gene phylogeny that a more in-depth phylogenetic investigation is needed to clarify the taxonomic status of the strains. The genus *Jatrophihabitans*, type species *Jatrophihabitans endophyticus* (Madhaiyan et al., 2013), comprises non-spore forming strains with short rod cells. *Jatrophihabitans* strains have been isolated from plants and soils. *Jatrophihabitans* sp. DSM 44399^T^ showed had 94.8% 16S rRNA gene similarity with the type strain of *Jatrophihabitans telluris* KCTC 39922^T^. These strains formed together a subclade closely related to *Jatrophihabitans endophyticus* DSM 45,627 ^T^.

The genus *Pseudonocardia,* with *Pseudonocardia thermophila* (Henssen, 1957) as the type species, contains strains characterized by the presence of substrate mycelium with various degree of branching and thickness and an aerial mycelium generating square or oval elements or a chain of spores (>2 nm) when fragmented (Riahi et al., 2022). They have been isolated from soils, plants, sediments, wastewater, a gold mine cave, or molds (Riahi et al., 2022). As shown in Figure S2, *Pseudonocardia* sp. DSM 45834^T^ is loosely associated with ‘*Pseudonocardia humida’* strain S2–4^T^ with 16S rRNA gene identity of 97.8%. However, *Streptomonospora* sp. DSM 45055^T^ appeared in a distinct branch close to *Streptomonospora litoralis* DSM 106425^T^, though the highest 16S rRNA gene similarity of 99.5% was obtained with the type strain of *Streptomonospora arabica* S186^T^ (Table S2).

Strains of the genus *Nocardiopsis* are widely distributed in the environment, including compost, animal, human clinical samples, plant material, the indoor environment and soil. They are fairly resistant to desiccation and play an important role in recycling organic substances due to their genetic ability to generate exoenzymes, surfactants, antibiotics that help them to survive under different conditions (Bennur et al., 2015). *Nocardiopsis* strains are filamentous bacteria with straight to flexuous or zigzag-shaped aerial mycelium and a smooth spore surface. *Nocardiopsis* sp. DSM 44743^T^ was loosely associated with *Nocardiopsis algeriensis* CECT 8712^T^ (99.3%) and adjacent to a poorly supported subcluster encompassing the type strain of *Nocardiopsis flavescens* (99.3%) and *Nocardiopsis lucentensis* (99.2%). Overall, it is clear from the 16S rRNA gene phylogeny that a single gene is not sufficient to distinguish between closely related species. Therefore, a genome-based tree was constructed.

### Phylogenomic and comparative genomics analyses show the actual relationships of the identified actinobacterial strains

#### Phylogenomic and comparative genomic analysis of Streptomyces strains

**Fig. 1:**
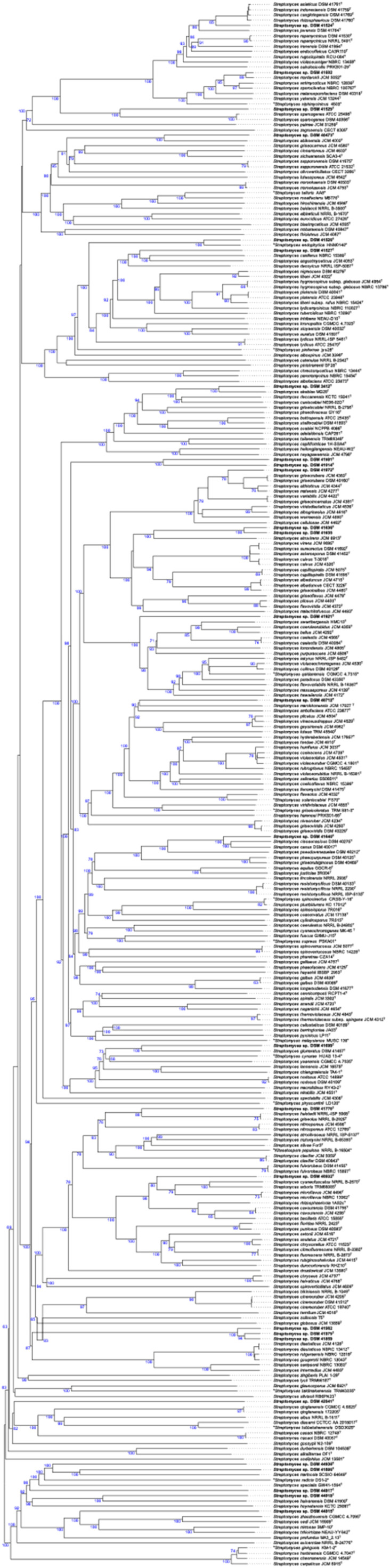
Phylogenomic tree showing the relationships between the *Streptomyces* strains and their closely related species. Tree inferred with FastME from GBDP distances calculated from genome sequences. The branch lengths are scaled in terms of GBDP distance formula *d5*.

In order to assign all the strains to their correct taxonomic rank with high resolution and to have a clear overview of their evolutionary radiation with the corresponding genera, a genome-based phylogeny was constructed. The genome blast distance phylogenetic (GBDP) tree shown in Fig. 1 has highly supported branches. The taxonomic description given below is in the descending order of the strains in the genomic tree. Strain DSM 41524^T^ was found in a distinct branch closely associated with *S. asiaticus* DSM 41761^T^, *S. indonesiensis* DSM 41759^T^, *S. cangkringensis* DSM 41769^T^, and *S. rhizosphaericus* DSM 41760^T^ (Fig. 1). The branch lengths of *S. indonesiensis, S. cangkringensis*, and *S. rhizosphaericus* in the genome-based tree reflected their evolutionary convergence and call for revision of the taxonomic status of these validly named species. A narrow range of dDDH values ranged was obtained between strain DSM 41524^T^ and *S. asiaticus* DSM 41761^T^ (71.6%), *S. indonesiensis* DSM 41759^T^ (71.5%), *S. cangkringensis* DSM 41769^T^ (71.5%), and *S. rhizosphaericus* DSM 41760^T^ (71.0%). However, the pairwise dDDH values between the genomic sequences of the type strains of S. *asiaticus, S. indonesiensis, S. cangkringensis*, and *S. rhizosphaericus* ranged between 96.2% to 98.4%, a value above the cut-off point of species demarcation. Therefore, *S. indonesiensis, S. cangkringensis*, and *S. rhizosphaericus* are heterotypic synonyms of *S. asiaticus*. These findings call for emending the description of the *S. asiaticus* species*.* The dDDH results between the genome sequence of strain DSM 41524^T^ and its close neighbours showed that strain DSM 41524^T^ forms a new subspecies within the *Streptomyces asiaticus* species for which the name *Streptomyces asiaticus* subsp. *ignotus* subsp. nov. is proposed**.**

In the same cluster of strain DSM 41524^T^, strain DSM 41602 formed a subcluster with *S. mordarskii* JCM 5052^T^ placed next to *S. antimycoticus* NBRC 12839^T^ and *S. sporoclivatus* NBRC 100767^T^ forming together a well-supported cluster (Fig. 1). *S. sporoclivatus* species is a later heterotypic synonym of *S. antimycoticus* species. The dDDH values between strain DSM 41602 and *S. mordarskii* JCM 5052^T^ was 93.5% while the scores obtained between the following pairs of strains DSM 41602 and *S. sporoclivatus* NBRC 100767^T^ (76.0%); DSM 41602 and *S. antimycoticus* NBRC 12839^T^ (75.6%); *S. mordarskii* JCM 5052^T^ and *S. antimycoticus* NBRC 12839 ^T^ (75.6%) were between the threshold, 70–79%, for prokaryotic subspecies delineation. Furthermore, the *in-silico* G+C content of strain DSM 41602 is 70.9%, while the type strain of *S. antimycoticus* has 70.9 mol%; value within species variation of 1%. Therefore, strain DSM 41602, *S. mordarskii* JCM 5052^T^, *S. sporoclivatus* NBRC 100767^T^ merit to be classified as a subspecies of *S. antimycoticus* for which the name *Streptomyces antimycoticus* subspecies *sporoclivatus* comb. nov. was proposed.

Fig. 1 showed that strain DSM 41529^T^ formed a well-supported sub-cluster with the type strains of *S. sparsogenes* (Fig. 1). The phylogenomic position of the strain and its branch length clearly showed that it forms a new species within the evolutionary radiation of the genus *Streptomyces*. This conclusion agreed with the dDDH value below the threshold of 70% for species demarcation between the genome sequence of strain DSM 41529^T^ and its close relative. Therefore, the strain DSM 41529^T^ is considered as a type strain of a new species for which the name *Streptomyces lonegramiae* sp. nov., is proposed.

The phylogenomic tree showed that strain DSM 40473^T^ was grouped with *S. abikoensis* JCM 4002^T^, forming a well-supported sub-cluster (Fig. 1). These results were in concordance with the dDDH value of 47.1% and justify the attribution of the strain to a new species, *Streptomyces hesseae* sp. nov.

In another phylogenetic lineage, strain DSM 41528^T^ formed a well-supported sub-cluster with ‘*S. endophytica* HNM01140’ (Zhou et al., 2023) and next to a subclade housed strain DSM 41527^T^ and its close relatives (Fig. 1). The dDDH value between strains DSM 41528^T^ and HNM01140 was 70.8%, value slightly above the borderline of the 70% threshold for species demarcation. The strain has a G+C content of 71.7% while the type strain of *Streptomyces endophytica* has 72.2%, a value within species variation of 1% G+C (JP Meier-Kolthoff et al., 2014). At the time of writing, the name of *S. endophytica* species (Zhou et al., 2023) was not yet validly published and it is unclear how distant *S. endophytica* is phylogenetically from *S. endophyticus* (Li et al., 2013), as the genome of *S. endophyticus* is unavailable. In addition, the species epithet *endophytica* leads to confusion with the validly named *S. endophyticus* species*.* According to rule 53 of the International code of nomenclature of Prokaryotes (revision 2022) ‘*an epithet is illegitimate if it duplicates a specific or subspecific epithet previously validly published for a species or subspecies’.* As only legitimate names and epithets are taken into consideration*,* strain DSM 41528^T^ cannot be affiliated to a new subspecies of ‘*S. endophytica’,* but it deserves to be considered as a novel species for which the name *Streptomyces bugieae* sp. nov. is proposed.

Strain DSM 41527^T^ appeared in a distinct branch closely associated with *S. caniferus* NBRC 15389^T^ and *S. angustmyceticus* JCM 4053^T^ (Fig. 1). The dDDH values between the strain and its close relatives (41.7–42.5 < 70%) permit the assignment of strain DSM 41527^T^ to a new *Streptomyces* species for which the name of *Streptomyces mooreae* sp. nov. is proposed.

In the next cluster, strain DSM 3412^T^ had a phylogenetic position closely associated with ‘*S. akebiae* MG28^T^’, together forming a subclade next to *S. deccanensis* KCTC 19241^T^ and *S. caniscabiei* NE06–02D^T^ (Fig. 1). The dDDH between the genome sequence of strain DSM 3412^T^ and its close relative ‘*S. akebiae* M G28^T^’ was between 64.7–46.8%, value below the 70% cut-off point for species demarcation (Table S3). Therefore, strains DSM 3412^T^ should be considered as the type strain of new species for which the name *Streptomyces gottesmaniae* sp. nov., is proposed.

Strains DSM 41981^T^ and DSM 41014^T^ formed a well-supported distinct group next to the subcluster of strain DSM 41972^T^ (Fig. 1) and showed a dDDH values below the 70% threshold for prokaryotic species affiliation. Therefore, strains DSM 41981^T^ and DSM 41014^T^ merit to be considered as novel species for which the names *Streptomyces doudnae* sp. nov., and *Streptomyces hintoniae* sp. nov. are proposed, respectively. Strain DSM 41972^T^ was closely related to the type strains of *S. griseorubens, S. althioticus,* and *S. matensis* species (Fig. 1). *S. griseorubens* and *S. matensis* are heterotypic synonyms of *S. althioticus*. The dDDH values between the genomic sequences of strain DSM 41972^T^ and those of *S. althioticus* JCM 4344^T^ (72.3%) and *S. matensis* JCM 4277^T^ (72.0%) fell between the established threshold for the prokaryotic subspecies demarcation, 70–79% (JP Meier-Kolthoff et al., 2013). Therefore, strain DSM 41972^T^ merits to be considered as a new sub-species of *S. althioticus* for which the name *Streptomyces althioticus* subsp. *attaecolombicae* subsp. nov. is proposed.

Strains DSM 41635 and DSM 41636^T^ formed a well-supported subclade closely related to the type strain of *S. atrovirens* JCM 6913^T^ (Fig. 1). The close phylogenomic relationship of strains DSM 41635 and DSM 41636^T^ was in line with a dDDH value of 82.1%, which is above the borderline of prokaryotic species delineation calling for affiliating them to the same species. The variation of the G+C content between strains DSM 41635 and DSM 41636^T^ is within the range of 1% defined within a species (JP Meier-Kolthoff et al., 2014). Moreover, these strains have very similar genomic features as shown in Table S3. However, the dDDH values between strains DSM 41635 and DSM 41636^T^ and validly named *Streptomyces* species were far below the 70% (Table S3). Thus, strains DSM 41635 and DSM 41636^T^ merit to be considered as a new species for which the name *Streptomyces edwardsiae* sp. nov. is proposed with strain DSM 41636^T^ as the type strain.

In a distant sub-clade from the strains listed above, strain DSM 41921^T^ was placed in a distinct branch which reflected its genetic divergence from the validly named reference strains (Fig. 1). These findings were consistent with the dDDH score (36.2%) below 70%. Thus, strain DSM 41921^T^ should be considered as new species within the genus *Streptomyces* for which the name *Streptomyces dubilierae* sp. nov. is proposed.

Strain DSM 40712^T^ was closely related to *S. ambofaciens* ATCC 23877^T^ and *S. marokkonensis* JCM 17027^T^, forming together a well-supported sub-group (Fig. 1). The genetic divergence of these strains was in line with the low dDDH values of 37.1–37.6%. Strain DSM 40712^T^ is therefore a type strain of a new species for which the name *Streptomyces lancefieldiae* sp. nov., is proposed.

Strain DSM 41640^T^ and its close relatives *S. ciscaucasicus* DSM 40275^T^ and *S. canus* DSM 40017^T^ (Fig. 1). However, *S. ciscaucasicus* is a later heterotypic synonym of *S. canus.* The divergent phylogenetic position of this strain within the evolution of the genus *Streptomyces* was justified by dDDH value below the cut-off point for species demarcation (Table S3). Thus, strain DSM 41640^T^ forms a novel species for which the name *Streptomyces doebereineriae* sp. nov. is proposed.

Strain DSM 41699^T^ was associated with *S. glomeratus* DSM 41457^T^ and next to ‘*S. cynarae* HUAS 13–4′ and *S. yaanensis* CGMCC 4.7035^T^ (Fig. 1). The dDDH values between the genome sequence of strain DSM 41699^T^ and its closest relatives listed above ranged from 30.8–32.3%. Thus, strain DSM 41699^T^ forms a new species within the genus *Streptomyces* for which the name *Streptomyces gibsoniae* sp. nov. is proposed.

Strain DSM 41770^T^ was closely related to *S. halstedii* NRRL ISP-5068^T^, an earlier heterotypic synonym of *S. griseolus*, forming together a well-supported sub-group adjacent to the *S. nitrosporeus* JCM 4598^T^ branch (Fig. 1). In another subcluster, DSM 40932^T^ was placed in a distinct branch closely associated with *S. cyaneofuscatus* NRRL B-2570^T^, *S. arboris* TRM68085^T^, and the type strains of *S. microflavus* (Fig. 1). The divergence of the strains DSM 41770^T^ and DSM 40932^T^ from validly named *Streptomyces* species is evidenced by dDDH values below the 70% threshold defined for species demarcation (Wayne et al., 1987) as shown in Table S3. Therefore, strains DSM 41770^T^ and DSM 40932^T^ merit to be considered as novel species for which the names *Streptomyces salyersiae* sp. nov., and *Streptomyces stephensoniae* sp. nov., are proposed, respectively.

Strains DSM 41979^T^, DSM 41859, and DSM 41982 formed together a well-supported sub-cluster related to a sub-clade encompassing the type strains of *S. diastaticus, S. rutgersensis, S. gourgerotii, S. coelicolor, S. limosus* and *S. albidoflavus* (Fig. 1). However*, S. rutgersensis, S. gourgerotii* are a later heterotypic synonym of *S. diastaticus* while *S. limosus* is a later heterotypic synonym of *S. albidoflavus*. The dDDH between the genome sequence of the studied strains DSM 41979^T^, DSM 41859, and DSM 41982 ranged from 80.1% to 87.7%; which is coherent with their close phylogenetic relationships and their similar genomic features, including the G+C content (Table S1-S2). However, the highest dDDH value between these studied strains and *Streptomyces* species validly named was 23.4%, a value below the threshold for species demarcation. Consequently, strains DSM 41979^T^, DSM 41859, and DSM 41982 form a new species for which the name *Streptomyces evansiae* sp. nov. is proposed with strain DSM 41979^T^ as the type strain.

In another phylogenetic lineage, the strain DSM 42041^T^ was placed in a distinct branch associated with a sub-clade containing the type strains of *S. qinglanensis, S. albus, S. diacarni, S. tubbatahanensis,* and *S. cacaoi*, and next to the branch of *S. grossypii* N2–109^T^ (Fig. 1). These results were consistent with the dDDH value below the cut-off point of 70% between the strain and all validly named *Streptomyces* species. Thus, strain DSM 42041^T^ merits to be affiliated to a new species for which the name *Streptomyces hazeniae* sp. nov., is proposed.

The distinct branch of strain DSM 44938^T^ was closely associated with a subclade that contained strains DSM 41886^T^ formed with *S. marincola* SCSIO 64649^T^ and next to *S. radicis* DS1–2^T^ (Fig. 1). Strains DSM 44917^T^ was found to be attached to this subclade with *S. specialis* GW41–1564^T^ as the closest neighbour. In the same cluster, strain DSM 44918^T^ was closely related to *S. hainanensis* DSM 41900^T^ forming together a well-supported subclade next to *Streptomyces hoynatensis* KCTC 29097^T^. These strains were adjacent to a sub-cluster encompassed strain DSM 44915^T^ and its closest phylogenetic neighbours *S. zhaozhouensis* CGMCC 4.7095^T^, *S. sedi* JCM 16909^T^, *S. mimosae* 3MP-10^T^, and *S. triticirhizae* NEAU-YY642^T^ (Fig. 1). These phylogenetic relationships between the strains and their close phylogenomic neighbours were in line with the dDDH values below the threshold of 70% for prokaryotic species demarcation (Wayne et al., 1987) (Table S3). Therefore, strains DSM 41886^T^, DSM 44938^T^, DSM 44917^T^, DSM 44918^T^, and DSM 44915^T^ form novel species for which the names *Streptomyces johnsoniae* sp. nov*., Streptomyces litchfieldiae* sp. nov., *Streptomyces boetiae* sp. nov., *Streptomyces millisiae* sp. nov., *Streptomyces chisholmiae* sp. nov. are proposed, respectively.

#### Phylogenomic and comparative genomic analysis of non-Streptomyces strains

**Fig. 2:**
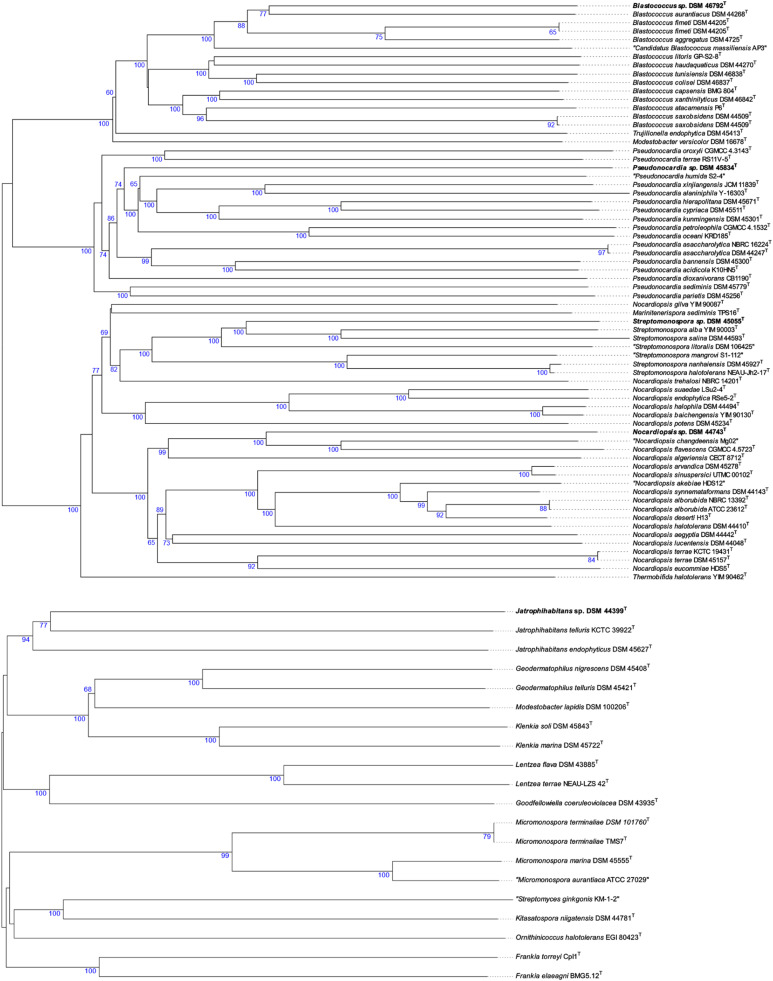
Phylogenomic tree showing the relationships between different actinobacterial strains and their closely related species. Tree inferred with FastME from GBDP distances calculated from genome sequences. The branch lengths are scaled in terms of GBDP distance formula *d*5.

The phylogenetic position of all the non-*Streptomyces* strains in the phylogenomic tree (Fig. 2) is well supported and a clear conclusion about the taxonomic status of the strains could be drawn. *Blastococcus* sp. DSM 46792^T^ formed a well-supported subclade with *Blastococcus aurantiacus* DSM 44268^T^ within the radiation of its respective genus (Fig. 2). These results were coherent with a dDDH value of 31.4% between strains DSM 46792^T^ and DSM 44268^T^. Therefore, strain DSM 46792^T^ deserves to be affiliated to a new species for which the name *Blastococcus goldschmidtiae* sp. nov. is proposed.

*Pseudonocardia* sp. DSM 45834^T^ was found to be a distinct branch closely related to a subcluster consisted of the type strains of ‘*Pseudonocardia humida’, Pseudonocardia xinjiangensis, Pseudonocardia alaniniphila, Pseudonocardia cypriaca, Pseudonocardia kunmingensis, Pseudonocardia petroleophila, Pseudonocardia abyssalis,* and *Pseudonocardia oceani* species (Fig. 2). The dDDH between strain DSM 45834^T^ and its close phylogenomic relatives listed above (<22.0%) were far below the 70% threshold for species demarcation as shown in Table S3 and call for assigning strain DSM 45834^T^ to a new *Pseudonocardia* species for which the name *Pseudonocardia charpentierae* sp. nov. is proposed. *Streptomonospora* sp. DSM 45055^T^ appeared in a distinct branch close to the type strains of *Streptomonospora alba* and *Streptomonospora salina* species and phylogenetically distant from *Streptomonospora arabica* (Fig. 2). These outcomes were in line with the low dDDH values (28.1–26.8%) between the genome sequence of strain DSM 45055^T^ and its close phylogenetic relatives. Thus, strain DSM 41055^T^ merits to be assigned to a new species for which the name *Streptomonospora wellingtoniae* sp. nov. is proposed.

*Nocardiopsis* sp. DSM 44743^T^ was placed in a well-supported distinct branch closely related to a subclade encompassed *Nocardiopsis flavescens* CGMCC 4.5723^T^ and ‘*Nocardiopsis changdeensis* Mg02^T^’ and next to *Nocardiopsis algeriensis* CECT 8712^T^ (Fig. 2). The dDDH between the genome of the studied strain and their close phylogenomic neighbours (24.2–29.1%) were below the 70% cut-off point for species demarcation. Therefore, strain DSM 44743^T^ forms a new species for which the name *Nocardiopsis lambiniae* sp. nov., is proposed. *Jatrophihabitans* sp. DSM 44399^T^ was grouped with *Jatrophihabitans telluris* KCTC 39922^T^ and alongside the type strain of *Jatrophihabitans endophyticus* (Fig. 2). The phylogenetic relationship of strain DSM 44399^T^ with its close relative is in line with the dDDH value of 20.4%, proving that the strain forms a new species for which the name *Jatrophihabitans lederbergiae* sp. nov. is proposed.

Genomic features including genome size, G+C content, number of RNA and coding sequences for all the studied strains and their closest phylogenomic neighbours are provided in Table S1.

### Cultural and growth properties of the novel actinomycetes strains

Most of the *Streptomyces* strains were able to grow from 10°C to 37°C with optimal growth between 28°C and 37°C. Strains DSM 40932^T^, DSM 3412^T^, DSM 40434^T^, DSM 41979^T^, DSM 41982, DSM 41859, and DSM 41770^T^ showed poor to moderate growth at 4°C and 10°C (Table S4). Only strains DSM 41981^T^ and DSM 41921^T^ showed a good growth at 45°C while strains DSM 44918^T^ and DSM 44938^T^ grew without aerial mycelia. All *Streptomyces* strains showed an optimal growth on DSMZ 65 medium and also on other media as shown in Table S3. A brown diffusible pigment was observed on ISP6 and ISP7 for strains DSM 41921^T^, DSM 3412^T^, DSM 41699^T^, while strains DSM 44917^T^ and DSM 44915^T^ produced a brown pigment on GYM and NZ amine media. In addition, strain DSM 42041^T^ produced brown pigment on NZ amine. More detail about the growth properties and the morphological features of the strains are presented in Table S3 and Figure S3.

Strains DSM 41635 and DSM 41636^T^, which were found to belong to the same new species, were distinguished from each other on the basis of their morphology. Strain DSM 41636^T^ developed lavender aerial mycelium after 7 days of incubation at 28°C on ISP2, while strain DSM 41635 had white-grey aerial mycelium under the same condition. Strains DSM 41979^T^, DSM 41859, and DSM 41982 had white aerial mycelium on ISP3, ISP5, ISP7 after 7 days of incubation at 28°C. Strain DSM 41859 showed white grey aerial mycelium on NZ amine medium unlike strains DSM 41979^T^ and DSM 41982, which had no aerial mycelium, after 7 days of incubation at 28°C. Strain DSM 41528^T^ showed a good growth on ISP1 to ISP7, DSMZ 535, GYM, and NZ amine media, on which it developed white aerial mycelium, which was greyish and pinkish on ISP1/ISP3 and NZ amine media, respectively. Moderate growth was observed at 25°C and 42°C, while good growth was recorded between 28°C and 37°C. However, the type strain of *S. endophytica* was not able to grow on ISP7 according to Zhou et al. 2023. Strain DSM 41972^T^, which forms a new subspecies within *S. althioticus,* had light grey (ISP1, ISP3, ISP6), white (ISP4, ISP5, ISP7), and light yellow (DSMZ 535, DSMZ 65, and NZ amine media) aerial mycelium after 7 days of incubation at 28°C. The strain showed weak growth at 17°C, 20°C, 42°C, and 45°C, while good growth was observed at 28°C, 35°C, and 37°C and at pH values between 5.0–9.0. Strain DSM 41602^T^ showed good growth on ISP1 to ISP6, GYM, and NZ amine medium, on which the strain developed white-lavender (ISP2), grey (ISP3 and ISP4), and white (GYM) aerial mycelia. No aerial mycelium was observed on ISP1, ISP5, ISP6, and NZ amine medium despite the good growth of yellow brown substrate mycelium. A moderate growth of the strain was obtained on ISP7 and DSMZ 535 media. No diffusible pigment was observed on any of the tested media. Growth was detected between 17°C to 37°C. These growth properties were in line with those reported for its close relative *S. antimycoticus* species by Komaki and Tamura, 2020. Strain DSM 41524^T^ showed a good growth on ISP1 to ISP7, DSMZ 535, DSMZ 65, and NZ amine agar plates on which aerial mycelium with grey (ISP1), white (ISP3, ISP4), and white greyish (ISP7) colour was detected. No aerial mycelium was observed on ISP2, ISP5, ISP6, DSMZ 535, and NZ amine, though a good development of yellow-brown substrate mycelium was obtained. A weak aerial mycelium was observed on medium DSMZ 65. No diffusible pigment was observed. The strain grew from 17°C to 42°C, whereby optimal growth was observed from 28°C to 37°C, unlike its close phylogenomic neighbour *S. asiaticus* DSM 1761^T^, which was able to grow at 45°C (Sembiring et al., 2000).

The growth properties for the non-*Streptomyces* strains were also determined and found to be in line with their respective genera*. Blastococcus* sp. DSM 46792^T^ showed an optimal growth on GYM, DSMZ 553, DSMZ 714, and DSMZ 877 media at 28°C. A well grown culture of the strain was obtained at 28°C, 35°C, and 37°C while poor to moderate growth was observed at 17°C to 25°C, respectively. No growth was observed at 42°C and 45°C.

*Streptomonospora* sp. DSM 45055^T^ showed a good growth on ISP1 (DSMZ 1764), DSMZ 514, and nutrient agar (DSMZ 1) media on which white-beige colonies were developed. The latter turned to have a dark brown colour on DSMZ 1159 medium. Optimal growth temperature was 28°C and at pH 6.0–7.0 on DSMZ 1159 medium. Moderate growth of this strain was obtained at 25°C, 35°C, and 37°C and on DSMZ 514 medium. *Pseudonocardia* sp. DSM 45834^T^ showed optimal growth at 28°C on GYM and DSMZ 83 media. Poor and moderate growth was observed at 17°C and at 20°C, 25°C, 35°C, and 37°C, respectively. The growth of *Nocardiopsis* sp. DSM 44743^T^ was poor at 4°C, 10°C, and 42°C, moderate at 17°C, 20°C, 25°C, and good at 28°C, 35°C, and 37°C (Table S1). Good growth was observed at pH 5.0 to 8.5 on GYM medium after 7 days of incubation. The strain showed white aerial mycelium at DSMZ 514 medium after 7 days of incubation at 28°C. *Jatrophihabitans* sp. DSM 44399^T^ formed orange colonies on medium 621, ISP2, Bennet's media after incubation at 20°C. The strain was able to grow well at 20°C and 25°C and at pH 5.5 to 8.5.

### Biochemical, enzymatic and chemotaxonomic analyses reveal the physiological properties of the strains

All the *Streptomyces* strains were distinguished from each other by their ability to metabolise a wide range of carbon and nitrogen sources. As shown in Table S5, most of the *Streptomyces* strains were unable to metabolise sugar alcohol substrates, such as L-arabitol, erythritol, xylitol, and D-sorbitol (except strain DSM 3412^T^). Strains DSM 41527^T^, DSM 41529^T^, DSM 41640^T^, DSM 41921^T^, DSM 44918^T^, DSM 44915^T^, and DSM 44938^T^ oxidized D-adonitol, whereas strains DSM 40712^T^ and DSM 41859 were able to use dulcitol.

The biochemical properties distinguished the studied strains, which belonged to the same new species (strains DSM 41635 and DSM 41636^T^; DSM 41859, DSM 41982, and DSM 41979^T^) from their close phylogenomic relatives (Table S5). The same applies to the studied strains DSM 41972^T^ and DSM 41602^T^, which form new subspecies. Strains DSM 41635 and DSM 41636^T^ were distinguished from each other by the ability of strain DSM 41635 to metabolise D-xylose, D-galactose, amygdalin, salicin, gentiobiose, and D-turanose and to produce ß-galactosidase and α-glucosidase, unlike strain DSM 41636^T^. The strains DSM 41979^T^, DSM 41859, and DSM 41982, which belong to the proposed new species, *Streptomyces sirexnoctilio* sp. nov. were differentiated from each other by the ability of strain DSM 41859^T^ to degrade dulcitol and the inability of strain DSM 41982 to use arbutin and salicin. However, strain DSM 41979^T^ and DSM 41859 were unable to oxidise D-melibiose and to produce α-galactosidase, respectively. More details about the biochemical and enzymatic profiles of the strains are shown in Table S5. The type strain of *S. endophytica* was able to use D-arabinose, D-xylose, L-rhamnose, D-mannitol, D-sorbitol (Zhou et al., 2023) unlike strain DSM 41528^T^. The culture of strain DSM 41528^T^ was prepared following the same growth condition (ISP2 at 28°C for 7 days) of its close phylogenomic neighbour.

DSM 41602^T^ forms a new subspecies within *S. antimycoticus* species. The strain was able to metabolise methyl-α-D-glucopyranoside unlike its close relative *S. antimycoticus* DSM 40284^T^. The latter oxidized dulcitol, inositol, D-sorbitol, methyl-α-S-mannopyranoside, amygdalin, D-saccharose, and inulin, unlike strain DSM 41602^T^. These strains had a very similar enzymatic profile though strain DSM 41602^T^ was able to produce lipase (C 14), while strain DSM 42084^T^ produced esterase (C 4) and esterase lipase (C 8). The two latter enzymes, cystine arylamidase and trypsin were produced by strain DSM 41972^T^ unlike its relative *S. althioticus* DSM 40092^T^ (Table S5).

For the non-*Streptomyces* strains, *Pseudonocardia* sp. DSM 45834^T^ shared a similar enzymatic profile with its close phylogenomic neighbour *Pseudonocardia kunmingensis* DSM 45301^T^ with the exception that it was unable to produce N-acetyl-ß-glucosaminidase, α-mannosidase, and α-fucosidase like its relative. *Nocardiopsis* sp. DSM 44743^T^ was able to metabolise arbutin while its close phylogenomic relative *Nocardiopsis flavescens* DSM 45786^T^ oxidized D-galactose, L-sorbose, dulcitol, D-sorbitol, starch, glycogen, D-tagatose, D-fucose, L-fucose, and potassium 5-ketogluconate. These strains had a similar enzymatic profile, though strain DSM 45786^T^ produced α-mannosidase unlike strain DSM 44743^T^. More data are provided in Table S5.

*Streptomonospora* sp. DSM 45055^T^ was differentiated from its close genomic neighbour *Streptomonospora alba* DSM 44588^T^ by its ability to metabolise D-arabitol, D-adonitol, N-acetylglucosamine, glycerol, erythritol, D-ribose, L-rhamnose, and D-lyxose (Table S5).

*Blastococcus* sp. DSM 46792^T^ was able to produce esterase C4, α-chymotrypsin and trypsin unlike its close neighbour *Blastococcus aurantiacus* DSM 44268^T^.

The chemotaxonomic traits of the genus *Streptomyces* are *LL*-diaminopimelic acid (*LL*-A_2_pm) in the cell wall peptidoglycan; a high amount of saturated isoand *ante*isofatty acids; and MK-9(H_6_) and MK-9(H_8_) as the predominant quinones. *Streptomyces* strains have a polar lipid pattern composed of diphosphatidylglycerol (DPG), phosphatidylethanolamine (PE), phosphatidylinositol (PI), and phosphatidylinositol mannosides (PIM) (Kämpfer, 2012). The genus *Nocardiopsis* is characterized by the presence of *meso*-diaminopimelic acid (*meso*-A_2_pm) in its cell wall peptidoglycan and by the predominance of menaquinone MK-10 with various hydrogens. Quinone with 9 and 11 isoprene units have been detected in minor quantities. The major polar lipids are PI, phosphatidylcholine (PC), phosphatidylglyerol (PG); and phosphatidylmethylethanolamine (PME). The main fatty acids are branched (Hozzein et al., 2012). The chemotaxonomic traits of *Pseudonocardia* consist of *meso*-A_2_pm in the peptidoglycan and arabinose and galactose as cell wall sugars. *Pseudonocardia* strains have MK-8 (H_4_) as the predominant menaquinone; glucosamine-containing phospholipids, PC, PG, PE, PME, and glucosamine-phospholipids (PLs) as major polar lipids. The fatty acids pattern is predominated by isobranched fatty acids with a 16-carbon chain (Huang et al., 2012).

*Blastococcus* strains have cell wall peptidoglycan with *meso*-A_2_pm; galactose and arabinose as whole cell sugars; isobranched fatty acids with the presence of *iso*C_16:1_, C_18:1_ ω9*c*, C_17:1_ ω8*c, iso*C_15:0_, and C_17:0_; MK-9 (H_4_) as the predominant menaquinone. The main polar lipids are PC, PI, PE, DPG, and PG (Stackebrandt et al., 2012). The genus *Jatrophihabitans* is characterized by the presence of *meso*-A_2_pm in the cell wall peptidoglycan with acyl-type N-glycolylated; MK-9 (H_4_) as the predominant quinone; DPG and unidentified PL, as well as aminolipids; and *iso*palmitic acid, C_18:1_ ω9c, *anteiso*C_17: 0_ and C_17:__1_ ω8c, as major fatty acid (Madhaiyan et al., 2013).

The chemotaxonomic features of all studied strains were coherent with those of their respective genera (Table S6). All the studied strains were distinguished from their closest neighbours by chemotaxonomic, biochemical and enzymatic traits.

### New actinobacterial species and their antimicrobial activities

**Fig. 3:**
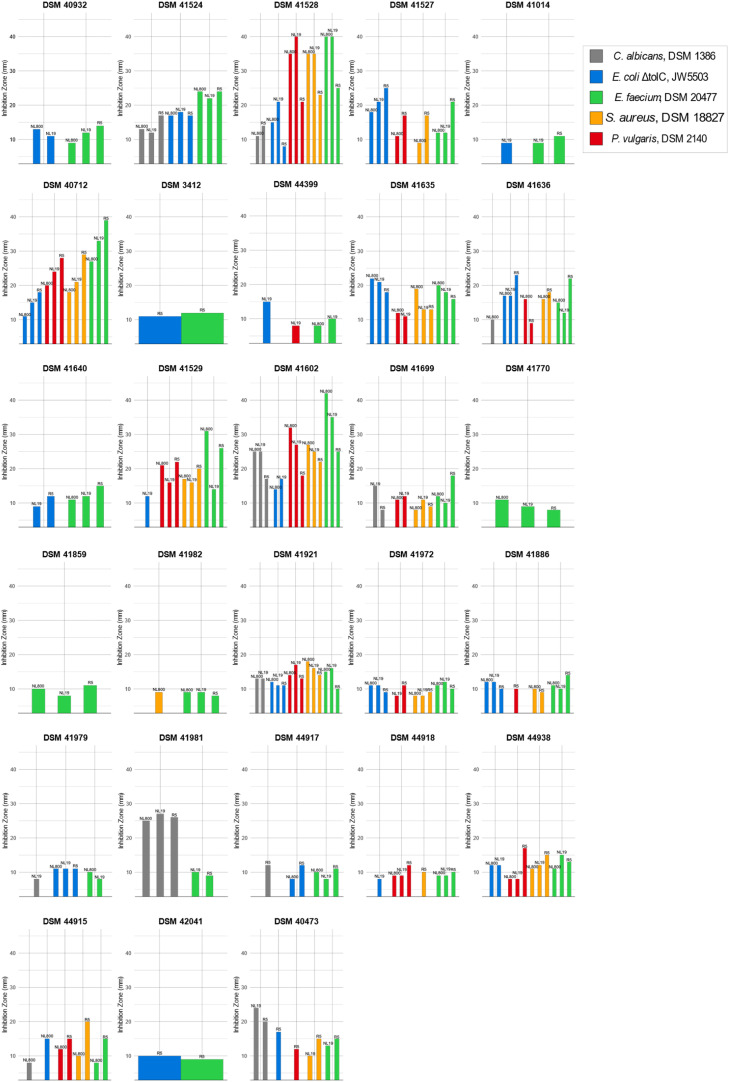
Antimicrobial activities of the studied strains against Gram-positive and Gram-negative bacteria, and yeast.

*Streptomyces* strains have been explored for new NPs and served as producers of not only hundreds of antibiotics but also enzymes, nanoparticles, or other industrially and pharmaceutically valuable biomolecules (Sanjivkumar et al., 2016, Sanjivkumar et al., 2018). In drug research, the discovery of the existence of numerous BGCs in the genomes of these strains has revived the research field (de Lima Júnior et al., 2023). The richness of the genetic composition of these filamentous bacteria in terms of specialized secondary metabolites, as well as the biotechnological advancements that have been successfully applied to *Streptomyces,* call for further exploiting the genetic diversity of this taxon, particularly after proving the effectiveness of taxonomy in drug discovery (Goodfellow and Fiedler, 2010, Thumar et al., 2010, Khodamoradi et al., 2021). It has been reported that *Nocardiopsis* strains produce toxins and immunomodulators, enzymes (amylases, cellulases, etc.) and bioactive compounds with antifungal, anticancer, and antimicrobial activities, such as pendolmycin or griseusins (Bennur et al., 2015). *Streptomonospora* strains are an interesting source of novel bioactive compounds, as shown by the discovery of two new anticancer compounds, named litoralimycin A1 and B2 from *Streptomonospora litoralis* (Khodamoradi et al., 2021), and an antibacterial compound named persiamycin A, which was isolated from *Streptomonospora* sp. PA3 (Matroodi et al., 2020). Some *Pseudonocardia* strains have been shown to be sources of interesting new bioactive compounds with antibacterial activity, such as branimycin and pseudonocardians A-C isolated from *P. carboxydivorans* M227 (Braña et al., 2017) and *Pseudonocardia* sp*.* SCSIO 01299 (Dekker et al., 1998, Li et al., 2011), respectively. Several anticancer compounds such as 4-(2-acetamidoethyl)-phenyl acetate and 4-((1,4-dioxooctahydropyrrolo [1,2-a] pyrazin-3-yl) methyl)-phenyl acetate were extracted from *P. endophytica* VUK-10 (Mangamuri et al., 2015, Mangamuri et al., 2016). Other strains (eg. *Pseudonocardia* sp. EC080529–01, *Pseudonocardia autotrophic*) produce bioactive compounds with antifungal activities, such as nystatin, garamycin, the polyene NPP A1, pseudonocardones A–C, 6-deoxy-8-O-methylrabelomycin (Mangamuri et al., 2016a, 2016b, Kim et al., 2017, Challinor and Bode, 2015, Barke et al., 2010, Sit et al., 2015), and the neuroprotective compound phenazostatin D, which was isolated from *Pseudonocardia* sp. B6273 (Maskey et al., 2003). Other strains appear to be more useful for bioremediation, such as *Pseudonocardia* sp. M43 (Lee et al., 2006), *Pseudonocardia* 1190 (Daye et al., 2003), *Pseudonocardia* sp. K1 (Kohlweyer et al., 2000), *Pseudonocardia* sp. KSF27 (Sakakibara et al., 2011), *Pseudonocardia* sp. RM423 (Apinya et al., 2015), *Pseudonocardia alni* AS4.1531 (Konkit and Lumyong, 2012), and *Pseudonocardia* sp. N23 (Derosa et al., 1996, Yamamoto et al., 2017). To assess the antibiotic potential of the strains, cell extracts were tested in bioassays against a panel of Gram-positive and Gram-negative bacterial test strains, as well as yeast, including clinically relevant pathogens, such as *Staphylococcus aureus* DSM 18,827, *Enterococcus faecium* DSM 20477^T^, *Proteus vulgaris* DSM 2140, *E. coli ΔtolC* JW5503–1, and *Candida albicans* DSM 1386, some of which are part of the recently established WHO Priority Pathogens list of the DSMZ (https://www.dsmz.de/collection/catalogue/microorganisms/special-groups-of-organisms/who-priority-pathogens-list). For some of the extracts, broad spectrum bioactivities were observed against all test strain. The vast majority of all *Streptomyces* strains exerted bioactivity against at least one microbial test strains as are summarized in Fig. 3.

However, no antimicrobial activities were detected from the crude extract of the non-*Streptomyces* strains.

### Gene cluster network analyses depict biosynthetic potential

**Fig. 4:**
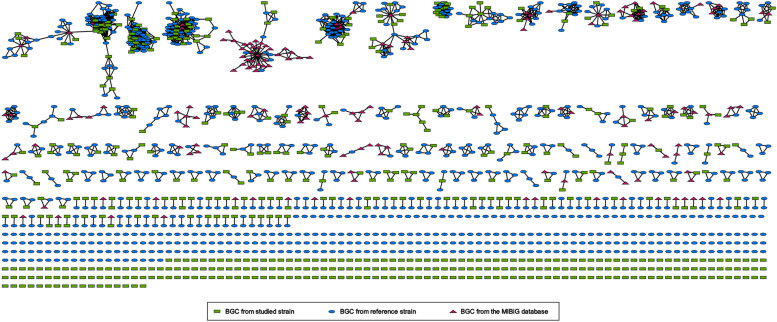
Gene cluster family network of GCFs as obtained from the BiG-SCAPE analysis and visualized with Cytoscape. Each node corresponds to a BGC. Green rectangles represent BGCs from the 32 studied strains, blue ovals are BGCs from reference strains, and purple triangles indicate known BGCs from the MIBiG database. Edges between two nodes represent a distance between the BGCs below the BiG-SCAPE default threshold of 0.3.

To investigate the BGC distribution between the identified strains (n=32) and their close phylogenomic neighbours (n=44), a gene cluster networking analysis was carried out using the BiG-SCAPE software with BGCs predicted by antiSMASH from seventy-six draft genome sequences in total. Additionally, the analysis included the MIBiG database to assess the novelty of the identified gene clusters. Due to the fragmented nature of the draft genomes, we separated the antiSMASH results into regions within contigs and on contig edges, and analyzed them separately. Out of 982 BGC regions predicted for the thirty-two identified strains (28 new type and 4 non-type strains), 407 were located on contig edges and 575 within contigs (Table S7). In the following, we will focus on the cluster networking analysis with BGC regions located within contigs to avoid overestimating biosynthetic diversity due to fragmented BGCs (Table S7). Together with the predicted BGC regions from the close phylogenetic neighbours and similar known BGCs from the MIBiG database (Terlouw et al., 2023), the BiG-SCAPE analysis of the regions inside contigs comprised 1508 BGC regions. BiG-SCAPE clustered these into 882 (excluding GCFs consisting only of MIBiG BGCs) gene cluster families (GCFs), of which 449 contained BGC regions from the newly identified strains (Fig. 4). A total of 265 out of these 449 GCFs were singletons. On the other hand, among the remaining 184 of the 449 GCFs, only 40 GCFs (comprising 65 BGCs from novel strains) were associated with a known BGC from MIBiG, indicating that few BGCs encode for the biosynthesis of known natural products (Table S7). For the other 144 GCFs (comprising 245 BGCs from novel strains), as well as the 265 singletons from novel strains, the associated NPs are unknown, showing a large potential of the respective strains for the production of novel compounds.

Among the 882 GCFs, 112 contained BGCs consisting of type I PKSs, 47 with PKS/NRPS-hybrid BGCs, and 259 with other PKS-related systems. Furthermore, 234 GCF contained NRP-related BGCs, 263 were associated with the production of RiPPs, and 149 with terpenes. Please note that some GCFs contain superclusters (BGC regions with multiple separate BGCs) and therefore fall into several of the above-mentioned classes.

In order to further analyse the relationship between the 28 novel strains and forty-four related strains, we performed a hierarchical clustering on the presence and absence of GCFs (only BGCs within contigs, singletons excluded). The resulting presence/absence map in combination with the dendrograms showed clearly distinguishable clusters (Figure S6). The grouping of these strains on the basis of GCFs is in line with their phylogenomic position within the genus *Streptomyces*. For instance, based on their GCFs, the novel strains DSM 41524^T^ and DSM 41602^T^ were grouped with the previously published strains *Streptomyces asiaticus* DSM 41761^T^ and *Streptomyces antimycoticus* NBRC 12839^T^ in a sub-cluster. This corresponds to their evolutionary relationship according to our phylogenomic analysis (Fig. 1). The cluster networking analysis revealed that these strains shared several GCFs, covering different types of BGCs (Figure S6) and it confirmed for some of the strains, such as DSM 41527^T^ and DSM 3412^T^, the presence of BGCs encoding for the previously identified compounds. Strain DSM 41527^T^, known as bialaphos producer, contained a BGC similar to the bialaphos BGC0000406 deposited in MiBIG, whereas the galbonolid producer strain DSM 3412^T^, harboured a BGC similar to the galbonolid BGC0000065 deposited in MiBIG. However, for several strains previously reported as antibiotic producers, the respective BGCs could not be identified, as for example strains DSM 40932^T^ (reported streptothricin producer), DSM 40712^T^ (reported chalcomycin producer), and DSM 41529^T^ (reported antibiotic A-130 producer). This could either be due to the fragmentation of the genomes and thus, missing BGCs, or indicate that the strains are not the original producers, but different strain deposits. The current standards for identifiying and confirming the authenticity of these old strains were not available at the time of deposition (between 1989–1993). Accordingly, it is worth analysing old strain deposits with new methods for their natural compound biosynthesis potential. In general, the current study can serve as preliminary data for further analytical chemistry research in the field of novel drug discovery.

### Identification of compounds from new actinobacterial type strains

**Fig. 5:**
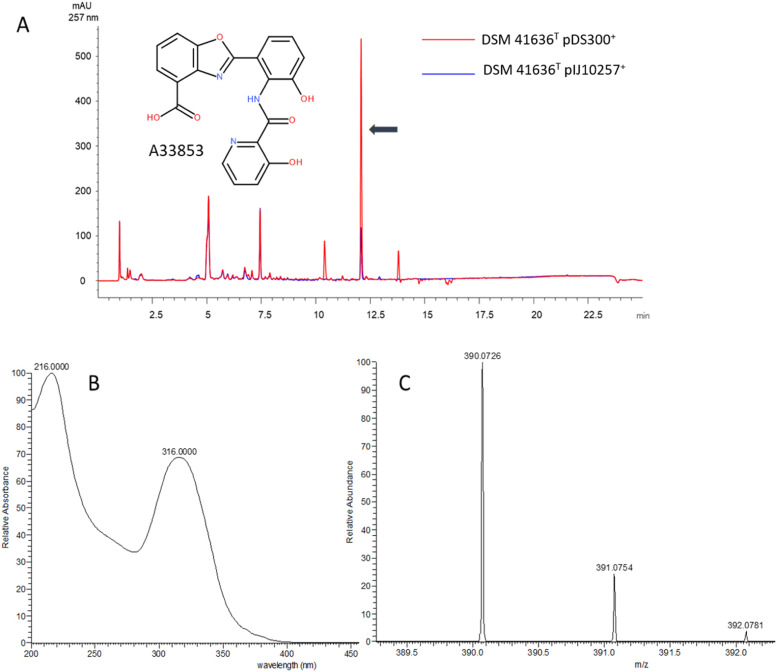
Detection of A33853 compound in extract samples of DSM 41636^T^::pDS300 by LC-MS analysis. (A) Overlaid chromatograms of ethyl acetate extracts derived from DSM 41636^T^ strains grown in R5. A peak corresponding to A33853 is indicated by a black arrow. (B) UV–Vis profile of the identified compound that is virtually identical to the previously reported spectrum of A33853 (Michel et al., 1984). (C) HRMS data of the DSM 41636^T^::pDS300 sample with a deprotonated molecular ion of A33853 (*m/z* [M – H]^–^ = 390.0726).

**Fig. 6:**
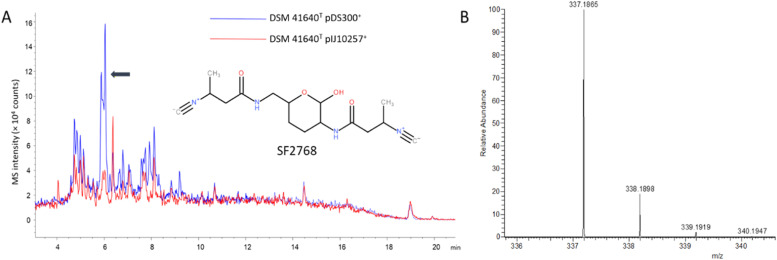
Detection of SF2768 in extract samples of DSM 41640^T^::pDS300 by LC-MS analysis. (A) Overlaid total ion chromatograms of ethyl acetate extracts derived from DSM 41640^T^ strains grown in NL19. A peak corresponding to SF2768 is indicated by a black arrow. (B) HRMS data of the DSM 41640^T^::pDS300 sample with a protonated molecular ion of SF2768 (*m/z* [M + H]^+^ = 337.1865).

In addition to guiding novel drug discovery, our BiG-SCAPE analysis provided further insight on known natural compounds and their underlying BGCs. In the course of studies on the regulator-guided activation of BGCs, the SARP gene *papR2* was heterologously expressed in strain DSM 41636^T^ and DSM 41640^T^, as similarly reported in Nouioui et al. (2024). Bioassays with samples from the DSM 41636^T^::pDS300 and DSM 41640^T^::pDS300 heterologous expression strains resulted in increased bioactivity against *K. rhizophila* in comparison to the respective control samples, carrying an empty vector (data not shown). Extracts obtained from 4-day old cultures were analyzed with LC-MS. Comparison of metabolic profiles resulted in the identification of the benzoxazole antibiotic A33853 as a product from strain DSM 41636^T^::pDS300 (Fig. 5) and the diisonitrile natural product SF2768 from DSM 41640^T^::pDS300 (Fig. 6).

The benzoxazole compound A33853 was first isolated from *Streptomyces* sp., NRRL 12068 and shows anti-leishmania activity (Michel et al., 1984). A BGC was identified in DSM 41636^T^ and the related strain DSM 41635, which showed 95% and 100% gene similarity in antiSMASH, respectively, to the A33853 BGC (MIBiG accession BGC0001292, (Lv et al., 2015)) (Figure S5 A-B).

SF2768 was first identified as a product from *Streptomyces* sp. (Tabata, 1995). The substance was originally isolated based on its fungicidal activity, but was also known for its bactericidal activity, later shown to be a copper-binding metallophore (Wang et al., 2017). A BGC was identified in DSM 41640^T^, which showed 66% gene similarity in antiSMASH to the SF2768 BGC (MIBiG accession BGC0001574) (Wang et al., 2017) (Figure S5 C) with all essential genes for SF2768 biosynthesis (SfaA-E from Wang et al, 2017) being present in the BGC of DSM 41640^T^. In accordance with the lower similarity, BiG-SCAPE clustered the BGC region from DSM 41640 with the published SF2768 BGC at a similarity threshold of 0.5, but not at the default threshold of 0.3.

Overall, genome-mining predicted two compounds from two strains of the set of ∼30 novel type strains from the DSMZ strain collection and SARP overexpression helped to confirm the production of these compounds.

Natural products have played an important role in a number of therapeutic areas such as cancer, infection, cardiovascular diseases (eg. statins), and sclerosis (eg. fingolimod). They are characterized by a wide range of molecular architecture and are liable to be improved, as illustrated by the RiPPs products, which can be predicted based on genome mining approaches using various computational tools and also can be modified by site-directed mutagenesis of their scaffolds (Delivoria et al., 2019, Zhong et al., 2020). NPs have more advantages than synthetic molecules, exemplified by their greater molecular rigidity, which can be very helpful in protein-protein interaction (Lawson et al., 2018, Atanasov et al., 2021). The large number of unknown BCGs suggest the genomic richness of the proposed novel type strains in producing novel secondary metabolites. These findings support previous findings that novel strains can lead to novel chemical entities and underline the importance of modern polyphasic taxonomy in the selection of biological material for novel drug discovery (Goodfellow et al., 2018 Aug, Goodfellow and Fiedler, 2010, Hoffmann et al., 2018). The strains included in this study were isolated decades ago and kept in a vital and stable form in the DSMZ's open collection. This report highlights the vital role of the culture collection in safeguarding microorganisms that can be very useful for novel drug discovery, health, food, and the environment, and therefore ensure the continuity of research studies. Conservation of microbial diversity and deposit in open culture collections should be essential for all beneficial strains and not only required for taxonomic description of novel type strain. This study provides an insight into the pharmaceutical and biotechnological potential of the proposed novel type strains, which are made available to the scientific communities (https://www.dsmz.de/collection/catalogue) for further research.

## Taxonomic consequences

### Description of *Streptomyces gottesmaniae* sp. nov

*Streptomyces gottesmaniae* (got.tes.ma'ni.ae. N.L. gen. fem. n. *gottesmaniae*, of Gottesman, named in honour of Susan Gottesman for her major contribution in the field of microbiology, biochemistry and molecular biology).

Gram-stain positive, aerobic actinobacterium with extensively branched substrate mycelium that develop white to grey aerial mycelium on ISP2, ISP3, and GYM media. The strain grows well from 25 to 42 °C, optimally at 28 °C, from pH 5.5 to 8.0, optimally at pH 7.0. The strain is able to assimilate D- and L-arabinose, D-arabitol, arbutin, D-cellobiose, D-fructose, L-fucose, D-galactose, gentiobiose, D-glucose, glycerol, glycogen, inositol, inulin, D-lactose (bovine origin), D-maltose, D-mannitol, D-mannose, D-melibiose, N-acetylglucosamine, potassium gluconate, potassium 5-ketogluconate, D-raffinose, L-rhamnose, D-ribose, D-saccharose (sucrose), D-sorbitol, starch (amidon), D-trehalose, and D-xylose. Positive for acid phosphatase, alkaline phosphatase, cystine arylamidase, esterase (C 4), esterase lipase (C 8), α-galactosidase, ß-galactosidase, α-glucosidase, ß-glucosidase, ß-glucuronidase, leucine arylamidase, lipase (C 14), α-mannosidase, N-acetyl-ß-glucosaminidase, naphthol-AS-BI-phosphohydrolase, and valine arylamidase. Whole cell sugars consist of glucose, ribose, with traces of mannose, and galactose. The peptidoglycan contains LL-A_2_pm and trace of DL-A_2_pm. The fatty acids profile comprises (>5%) *ante*isoC_15:0_, C_15:0_, C_16:0_, *iso*C_15:0_, *iso*C_16:0_, C_16:1_ cis9, *ante*isoC_17:0_. Polar lipid pattern consists of diphosphatidylglycerol, phosphatidylinositol, phosphatidylethanolamine, unidentified phospholipid, phosphoaminolipid, phosphoglycolipid. The strain was known to produce galbonolides A and B. The genome size of strain DSM 3412^T^ is 11.2 Mb with DNA G+C content of 70.0%.

The type strain DSM 3412^T^ (= Tü 2253^T^ = KCTC 59181^T^) was isolated from soil sample collected in Tunisia. The GenBank accession number of the assembled draft genome is JAVRFJ000000000.1.

### Description of *Streptomyces hesseae* sp. nov

*Streptomyces hesseae* (hes'se.ae. N.L. gen. fem. n. *hesseae*, of Hesse, in honour of Fanny Hesse (1850–1934) who introduced agar into microbiology).

Gram-stain positive, aerobic actinobacterium with extensively branched substrate mycelium that develop white greyish to grey aerial mycelium on ISP1, ISP2, ISP5, ISP6, DSMZ 535, GYM, and NZ amine media that turn to have white beige and light green colour on ISP3, and ISP7 media, respectively. The strain grows well from 25 to 45°C, optimally at 28°C, from pH 5.5 to 8.0, optimally at pH 7.0. The strain is able to assimilate D-arabitol, D-glucose, glycerol, glycogen, D-mannose, N-acetylglucosamine, potassium gluconate, D-ribose, and starch (amidon). Positive for acid phosphatase, alkaline phosphatase, esterase (C 4), esterase lipase (C 8), leucine arylamidase, naphthol-AS-BI-phosphohydrolase, and valine arylamidase. Whole cell sugars consist of glucose, ribose, with trace of mannose. The peptidoglycan contains LL-A_2_pm. The fatty acids profile comprises (>5%) *anteiso*C_15:0_, *iso*C_15:0_, C_16:0_, *iso*C_16:0_, *iso*C_17:0_, *anteiso*C_17:0_. Polar lipid pattern consists of diphosphatidylglycerol, phosphatidylinositol, phosphatidylethanolamine, phospholipid, phosphoglycolipid, and lipids. The genome size of strain DSM 40473^T^ is 7.6 Mb with DNA G+C content of 71.7%.

The type strain DSM 40473^T^ (= ATCC 12568^T^ = CBS 695.72^T^ = IFO 13394^T^ = IFO 13907^T^ = BA-3572^T^ = ISP 5473^T^ = KCC S-0694^T^ = KCC S-0812^T^ = NBRC 13394^T^ = NBRC 13907^T^ = RIA 1355^T^) was isolated from soil with unknown origin, but known as producer of antibiotic PA-150. The GenBank accession number of the assembled draft genome is JAVRFI000000000.1.

### Description of *Streptomyces lancefieldiae* sp. nov

*Streptomyces lancefieldiae* (lan.ce.fi.el'di.ae. N.L. gen. fem. n. *lancefieldiae,* of Lancefield, in honour of Rebecca Lancefield (1895–1981) known for her significant contributions to microbiology, particularly bacteriology).

Gram-stain positive, aerobic actinobacterium with extensively branched substrate mycelium that develop white aerial mycelium on ISP2, TSA, and NZ amine agar media that turn to have lavender colour on ISP3, ISP4, ISP5, ISP7, and GYM media. The strain grows well from 25 to 45°C, optimally at 28°C, from pH 5.5 to 8.0, optimally at pH 7.0. The strain is able to assimilate amygdalin, L-arabinose, D-arabitol, arbutin, D-cellobiose, dulcitol, D-fructose, D-galactose, D-glucose, glycerol, glycogen, inositol, inulin, D-lactose (bovine origin), D-maltose, D-mannitol, D-mannose, methyl-α-D-glucopyranoside, methyl-α-D-mannopyranoside, N-acetylglucosamine, potassium gluconate, L-rhamnose, D-ribose, salicin, and starch (amidon). Positive for acid phosphatase, alkaline phosphatase, α-chymotrypsin, esterase (C 4), esterase lipase (C 8), α-galactosidase, ß-galactosidase, α-glucosidase, leucine arylamidase, α-mannosidase, N-acetyl-ß-glucosaminidase, naphthol-AS-BI-phosphohydrolase, and valine arylamidase. Whole cell sugars consist of ribose, with traces of glucose, mannose, and galactose. The peptidoglycan contains LL-A_2_pm. The fatty acids profile comprises (>5%) *ante*isoC_15:0_, *iso*C_15:0_, *anteiso*C_17:0_, C_16:0_, *iso*C_16:0_, C_16:1_ cis9. Polar lipid pattern consists of diphosphatidylglycerol, phosphatidylinositol, phosphatidylethanolamine, unknown lipid, phospholipid, aminolipid, phosphoglycolipid, and phosphoaminolipid. The genome size of strain DSM 40712^T^ is 9.3 Mb with DNA G+C content of 71.4%.

The type strain DSM 40712^T^ (= NRRL 2835^T^ = Tü 41^T^ = ETH 21066^T^) is with unknown isolation source. The GenBank accession number of the assembled draft genome is JAVRFH000000000.1.

### Description of *Streptomyces stephensoniae* sp. nov

*Streptomyces stephensoniae* (s*te.phen.so'ni.ae.* N.L. gen. fem. n. *stephensoniae,* of Stephenson, named in honour of Marjory Stephenson (1885–1948) for her significant contributions to biochemistry and microbiology).

Gram-stain positive, aerobic actinobacterium with extensively branched substrate mycelium that develop orange aerial mycelium on ISP2 and GYM media that turn to have sandy yellow and white colour on ISP3 and DSMZ 535 media, respectively. The strain grows well from 20 to 37°C, optimally at 28°C, from pH 5.5 to 8.5, optimally at pH 7.0. The strain is able to assimilate amygdalin, D-arabitol, arbutin, D-cellobiose, gentiobiose, D-glucose, glycerol, glycogen, D-maltose, D-mannitol, D-mannose, N-acetylglucosamine, potassium gluconate, D-ribose, salicin, starch (amidon), and D-trehalose. Positive for acid phosphatase, alkaline phosphatase, α-chymotrypsin, cystine arylamidase, esterase lipase (C 8), ß-galactosidase, α-glucosidase, ß-glucosidase, leucine arylamidase, lipase (C 14), N-acetyl-ß-glucosaminidase, naphthol-AS-BI-phosphohydrolase, trypsin, and valine arylamidase. Whole cell sugars consist of glucose, ribose with traces of mannose and galactose. The peptidoglycan contains LL-A_2_pm. The fatty acids profile comprises (>5%) *iso*C_15:0_, *anteiso*C_15:0_, *anteiso*C_17:0_, C_16:0_, *iso*C_16:0_. Polar lipid pattern consists of diphosphatidylglycerol, phosphatidylinositol, phosphatidylethanolamine, unidentified phospholipids, aminolipid, phosphoglycolipid. The strain was known to produce streptolins A and B, streptothricin, vitamin B12. The genome size of strain DSM 40932^T^ is 7.9 Mb with DNA G+C content of 72.0%.

The type strain DSM 40932^T^ (= ATCC 13741^T^ = ATCC 13793^T^ = CBS 372.58^T^ = ETH 24437^T^ = NRRL B-1354^T^) is with unknown origin. The GenBank accession number of the assembled draft genome is JAVRFG000000000.1.

### Description of *Streptomyces hintoniae* sp. nov

*Streptomyces hintoniae* (hin.to'ni.ae. N.L. gen. fem. n. *hintoniae*, of Hinton, named in honour of Jane Hinton for her contributions to microbiology, particularly for co-developing the Mueller-Hinton agar).

Gram-stain positive, aerobic actinobacterium with extensively branched substrate mycelium that develop beige aerial mycelium on DSMZ 535 and GYM media. The strain grows well from 25 to 28°C, optimally at 28°C, from pH 5.5 to 8.0, optimally at pH 7.0. The strain is able to assimilate amygdalin, L-arabinose, D-arabitol, arbutin, D-cellobiose, D-fructose, D-fucose, D-galactose, gentiobiose, D-glucose, glycerol, glycogen, inositol, D-lactose, D-maltose, D-mannitol, D-mannose, methyl-α-D-glucopyranoside, N-acetylglucosamine, potassium gluconate, potassium 2-ketogluconate, L-rhamnose, D-ribose, salicin, (bovine origin), starch (amidon), D-trehalose, D-turanose, and D-xylose. Positive for acid phosphatase, alkaline phosphatase, cystine arylamidase, esterase (C 4), esterase lipase (C 8), α-fucosidase, ß-galactosidase, α-glucosidase, ß-glucosidase, leucine arylamidase, lipase (C 14), α-mannosidase, N-acetyl-ß-glucosaminidase, naphthol-AS-BI-phosphohydrolase, and valine arylamidase. Whole cell sugars consist of glucose and ribose with trace of mannose. The peptidoglycan contains LL-A_2_pm. The fatty acids profile comprises (>5%) *iso*C_14:0_, *ante*isoC_15:0_, *ante*isoC_17:0_, *iso*C_15:0_, *iso*C_16:0_, C_16:0_. Polar lipid pattern consists of diphosphatidylglycerol, phosphatidylinositol, phosphatidylethanolamine, lipid, phospholipids, phosphoglycolipid. The genome size of strain DSM 41014^T^ is 9.0 Mb with DNA G+C content of 72.0%.

The type strain DSM 41014^T^ (= IMRU 3066^T^ = KCTC 59176^T^) was isolated from *Sphagnum* pots with unknown origin. The GenBank accession number of the assembled draft genome is JAVRFF000000000.1.

### Description of *Streptomyces asiaticus* subsp*. ignotus* subsp. nov

*Streptomyces asiaticus* subsp*. ignotus* (i.gno'tus. L. masc. adj. *ignotus*, unknown, referring to the unknown origin of the strain).

Gram-stain positive, aerobic actinobacterium with extensively branched substrate mycelium that develop grey (on ISP1 medium), white- (ISP3, ISP4, DSMZ 65), and white grey aerial mycelium (on ISP7 medium) after 7 days of incubation at 28°C. The strain grows well at 25°C, 28°C, 35°C to 37°C, optimally at 28°C, from pH 5.5 to 9.0, optimally at pH 7.0. The strain is able to assimilate L-arabinose, glycerol, D-ribose, D-galactose, D-glucose, D-fructose, D-mannose, L-rhamnose, inositol, D-mannitol, methyl-a-D-mannopyranoside, methyl-a-D-glucopyranoside, N-acetylglucosamine, salicin, D-cellobiose, D-trehalose, D-raffinose, starch (amidon), glycogen, gentiobiose, potassium gluconate, and potassium 2-ketogluconate. The strain shows positive reactions for alkaline phosphatase, esterase lipase (C 8), lipase (C 14), leucine arylamidase, valine arylamidase, cystine arylamidase, trypsin, a-chymotrypsin, acid phosphatase, naphthol-AS-BI-phosphohydrolase, ß-galactosidase, ß-glucuronidase, and N-acetyl-ß-glucosaminidase. The whole cell sugars consist of glucose, mannose, and ribose. The peptidoglycan contains LL-A_2_pm. The fatty acids profile comprises (>5%) *iso*C_14:0_, *ante*isoC_15:0_, C_16:0_, *iso*C_15:0_, *iso*C_16:0_. Polar lipid pattern consists of diphosphatidylglycerol, phosphatidylinositol, phosphatidylethanolamine, phosphatidylmethylethanolamine, and unidentified lipids, phospholipids, and phosphoglycolipids. The genome size of strain DSM 41524^T^ is 11.4 Mb with DNA G+C content of 75%.

The type strain DSM 41524^T^ (= ATCC 15166^T^ = A10598^T^) was isolated from soil of unknown origin. The GenBank accession number of the assembled draft genome is JAZBJO000000000.

### Description of *Streptomyces mooreae* sp. nov

*Streptomyces mooreae* (moore'ae. N.L. gen. fem. n. *mooreae*, of Moore, in honour of Ruth Ella Moore (1903-1994) for her great work in the field of bacteriology).

Gram-stain positive, aerobic actinobacterium with extensively branched substrate mycelium that develop white greyish to grey aerial mycelium on ISP1, ISP2, ISP3, ISP4, ISP5, ISP6, and ISP7 media that turn to have white to white beige colour on TSA, GYM, and NZ amine media. The strain grows well from 25 to 28°C, optimally at 28°C, from pH 5.5 to 8.0, optimally at pH 7.0. The strain is able to assimilate D-adonitol, D-arabitol, D-galactose, D-glucose, glycerol, glycogen, L-fucose, inositol, D-maltose, D-mannitol, D-mannose, D-melibiose, N-acetylglucosamine, potassium gluconate, D-raffinose, D-ribose, D-saccharose (sucrose), starch (amidon), D-trehalose, D-turanose, and D-xylose. Positive for acid phosphatase, alkaline phosphatase, esterase lipase (C 8), ß-galactosidase, leucine arylamidase, N-acetyl-ß-glucosaminidase, naphthol-AS-BI-phosphohydrolase, and valine arylamidase.

Whole cell sugars consist of glucose, ribose, with traces of mannose and galactose. The peptidoglycan contains LL-A_2_pm. The fatty acids profile comprises (>5%) *iso*C_15:0_, *ante*isoC_15:0_, *ante*isoC_17:0_, *iso*C_17:0_, C_16:0_, *iso*C_16:0_. Polar lipid pattern consists of diphosphatidylglycerol, phosphatidylethanolamine, phosphatidylmethylethanolamine, phosphatidylinositol, unidentified phospholipids, lipids, and phosphoglycolipid. The strain is known as producer of bialaphos. The genome size of strain DSM 41527^T^ is 8.4 Mb with DNA G+C content of 71.1%.

The type strain DSM 41527^T^ (= ATCC 21705^T^ = SF-1293^T^) is with unknown origin. The GenBank accession number of the assembled draft genome is JAVRFE000000000.1.

### Description of *Streptomyces bugieae* sp. nov

*Streptomyces bugieae* (bu'gie.ae. N.L. gen. fem. n. *bugieae*, of Bugie, in honour of Elizabeth Bugie for her role in the discovery of streptomycin, the first antibiotic effective against tuberculosis).

Gram-stain positive, aerobic actinobacterium with extensively branched substrate mycelium that develop white-greyish (on ISP1, ISP2, ISP3 media), white (ISP4, ISP5, ISP6, ISP7, DSMZ 535, DSMZ 65), and white pinkish aerial mycelium (on DSMZ 553) after 7 days of incubation at 28 °C. The strain grows well at 28°C, 35 °C to 37 °C, optimally at 28 °C, from pH 5.5 to 9.0, optimally at pH 7.0. The strain is able to assimilate D-arabitol, glycerol, D-ribose, D-adonitol, D-galactose, D-glucose, D-fructose, D-mannose, D-mannitol, D-sorbitol, N-acetylglucosamine, D-maltose, D-melibiose, D-saccharose, D-trehalose, D-melezitose, D-raffinose, starch (amidon), glycogen, xylitol, D-turanose, and potassium gluconate. The strain shows positive reactions for alkaline phosphatase, esterase (C 4), esterase lipase (C 8), lipase (C 14), leucine arylamidase, valine arylamidase, cystine arylamidase, trypsin, a-chymotrypsin, acid phosphatase, naphthol-AS-BI-phosphohydrolase, ß-galactosidase, ß-glucuronidase, α-glucosidase, ß-glucosidase, and N-acetyl-ß-glucosaminidase. Whole cell sugars consist of galactose, glucose, mannose, and ribose. The peptidoglycan contains LL-A_2_pm. The fatty acids profile comprises (>5%) *ante*isoC_15:0_, *ante*isoC_17:0_, *iso*C_17:0_, C_16:0_, *iso*C_15:0_, *iso*C_16:0._ Polar lipid pattern consists of diphosphatidylglycerol, phosphatidylinositol, phosphatidylethanolamine, phosphatidylmethylethanolamine, unidentified lipids, phospholipid, and phosphoglycolipid, aminolipid. The strain was found to produce amylase. The genome size of strain DSM 41528^T^ is 9.0 Mb with DNA G+C content of 71.7%.

The type strain, DSM 41528^T^ (= ATCC 21722^T^ = FERM-P 602^T^ = SF-1084^T^) is with unknown origin. The GenBank accession number of the assembled draft genome is JAZBJP000000000.

### Description of *Streptomyces lonegramiae* sp. nov

*Streptomyces lonegramiae* (lo.ne.gra'mi.ae. N.L. gen. fem. n. *lonegramiae*, of Lone Gram, in honour of Lone Gram for her significant contributions to microbial secondary metabolite research).

Gram-stain positive, aerobic actinobacterium with extensively branched substrate mycelium that develop white aerial mycelium on ISP1, ISP2, ISP4, ISP7, TSA, and GYM media that turn to have light grey colour on ISP3 medium. The strain grows well from 25 to 28 °C, optimally at 28 °C, from pH 6.0 to 8.0, optimally at pH 7.0. The strain is able to assimilate D-adonitol, amygdalin, D- and L-arabinose, D-arabitol, arbutin, D-cellobiose, D-fructose, L-fucose, D-galactose, gentiobiose, D-glucose, glycerol, glycogen, inositol, D-lactose (bovine origin), D-maltose, D-mannitol, D-mannose, D-melibiose, N-acetylglucosamine, potassium gluconate, potassium 2-ketogluconate, potassium 5-ketogluconate, D-raffinose, L-rhamnose, D-ribose, D-saccharose (sucrose), salicin, starch (amidon), and D-xylose. Positive for acid phosphatase, alkaline phosphatase, leucine arylamidase, N-acetyl-ß-glucosaminidase, naphthol-AS-BI-phosphohydrolase, and valine arylamidase. Whole cell sugars consist of glucose, ribose, with traces of mannose, and galactose. The peptidoglycan contains LL-A_2_pm. The fatty acids profile comprises (>5%) *ante*isoC_15:0_, *ante*isoC_17:0_, C_16:0_, *iso*C_15:0_, *iso*C_16:0_. Polar lipid pattern consists of diphosphatidylglycerol, phosphatidylinositol, phosphatidylethanolamine, phosphatidylmethylethanolamine, unidentified phosphoglycolipid, and phospholipid. The strain was known to produce antibiotic A-130. The genome size of strain DSM 41529^T^ is 11.9 Mb with DNA G+C content of 71.2%.

The type strain DSM 41529^T^ (= FERM-P 639^T^ = ATCC 21840^T^ = A-130^T^) was isolated from a soil collected in Ikeda City in Japan. The GenBank accession number of the assembled draft genome is JAVRFD000000000.1.

### Description of *Streptomyces antimycoticus* subsp. *sporoclivatus* (Preobrazhenskaya 1986 ex Krassilnikov 1970) comb. nov

*Streptomyces antimycoticus* subsp. *sporoclivatus* (spo.ro.cli'va.tus. N.L. masc. adj. *clivatus*; N.L. masc. adj. *sporoclivatus*).

Basonym: *Streptomyces sporoclivatus* (*ex* Krassilnikov 1970) Preobrazhenskaya 1986

The description is the same as provided by Gauze et al. 1983 and Komaki and Tamura (2020).

The genome size of strain NBRC 100767^T^ is 11.0 Mb with DNA G+C content of 70.9%.

The type strain DSM 41461^T^ = ATCC 43693^T^ = INMI 97^T^ = JCM 9094^T^ = NBRC 100767^T^ = VKM Ac-315^T^ was isolated from soil. The GenBank accession number of the assembled genome is AP019620.1.

### Description of *Streptomyces edwardsiae* sp. nov

*Streptomyces edwardsiae* (ed.ward'si.ae. N.L. gen. fem. n. *edwardsiae*, of Edwards, in honour of Katrina Jane Edwards for her significant contributions to soil microbiology).

Gram-stain positive, aerobic actinobacterium with extensively branched substrate mycelium that develop grey aerial mycelium on ISP1, ISP2, and GYM media. The strain grows well from 20 to 37 °C, optimally at 28 °C, from pH 5 to 8.5, optimally at pH 7.0. The strain is able to assimilate L-arabinose, D-arabitol, D-cellobiose, D-fructose, D-glucose, glycerol, glycogen, inositol, D-lactose (bovine origin), D-maltose, D-mannitol, methyl-α-D-glucopyranoside, N-acetylglucosamine, potassium gluconate, L-rhamnose, D-ribose, starch (amidon) and D-trehalose. Positive for alkaline phosphatase, esterase (C 4), esterase lipase (C 8), leucine arylamidase, lipase (C 14), and valine arylamidase.

Whole cell sugars consist of glucose, ribose with traces of galactose and mannose. The peptidoglycan contains LL-A_2_pm. The fatty acids profile comprises (>5%) *ante*isoC_15:0_, *ante*isoC_17:0_, C_16:0_, *iso*C_16:0_, isoC_15:0_, C_16:1_ cis9. Polar lipid pattern consists of diphosphatidylglycerol, phosphatidylinositol, phosphatidylethanolamine, phospholipids, and phosphoglycolipid. The genome size of strain DSM 41636^T^ is 7.9 Mb with DNA G+C content of 71.2%.

The type strain DSM 41636^T^ (= 31-A2^T^ = KCTC 59179^T^) was isolated from a rose root, *Rosa laxa*, collected in Germany. The GenBank accession number of the assembled draft genome is JAVRFA000000000.1.

### Description of *Streptomyces doebereineriae* sp. nov

*Streptomyces doebereineriae* (doe.be.rei.ne'ri.ae. N.L. gen. fem. n. *doebereineriae*, of Döbereiner, in honour of Johanna Döbereiner (1924–2000) for her significant contributions in the field of plant-microbe interaction).

Gram-stain positive, aerobic actinobacterium with extensively branched substrate mycelium that develop white aerial mycelium on ISP2, TSA, and NZ amine agar media that turn to have lavender colour on ISP3, ISP4, ISP5, ISP7, and GYM media. The strain grows well from 25 to 45 °C, optimally at 28 °C, from pH 5.5 to 8.0, optimally at pH 7.0. The strain is able to assimilate D-adonitol, L- and D-arabinose, D-arabitol, D-cellobiose, D-fructose, L-fucose, D-galactose, gentiobiose, D-glucose, glycerol, glycogen, inositol, inulin, D-lactose, D-maltose, D-mannitol, D-mannose, D-melibiose, N-acetylglucosamine, potassium gluconate, potassium 5-ketogluconate, D-raffinose, L-rhamnose, D-ribose, D-saccharose, starch, and D-xylose. Positive for acid phosphatase, alkaline phosphatase, cystine arylamidase, esterase (C 4), esterase lipase (C 8), ß-galactosidase, α-glucosidase, ß-glucosidase, leucine arylamidase, lipase (C 14), naphthol-AS-BI-phosphohydrolase, and valine arylamidase. Whole cell sugars consist of glucose, ribose, rhamnose with traces of mannose and galactose. The peptidoglycan contains LL-A_2_pm. The fatty acids profile comprises (>5%) *iso*C_15:0_, *ante*isoC_15:0_, *ante*isoC_17:0_, C_16:0_, *iso*C_16:0_, C_16:1_ cis9, *iso*C_17:0_. Polar lipid pattern consists of diphosphatidylglycerol, phosphatidylinositol, phosphatidylethanolamine, phospholipids, phosphoglycolipid. The genome size of strain DSM 41640^T^ is 11.2 Mb with DNA G+C content of 70.0%.

The type strain DSM 41640^T^ (= 13-A30^T^ = KCTC 59177^T^) was isolated from a rose root, *Rosa laxa*, collected in Germany. The GenBank accession number of the assembled draft genome is JAVREZ000000000.1.

### Description of *Streptomyces gibsoniae* sp. nov

*Streptomyces gibsoniae* (gib.so'ni.ae. N.L. gen. fem. n. *gibsoniae*, of Gibson, named in honour of Jane Gibson for her great contributions in the field of microbial physiology and biochemistry).

Gram-stain positive, aerobic actinobacterium with extensively branched substrate mycelium that develop grey, white, and light grey aerial mycelium on ISP2, ISP3, and GYM media, respectively. The strain grows well from 25 °C to 37 °C, optimally at 28 °C, from pH 5.5 to 8.5, optimally at pH 7.0. The strain is able to assimilate D-arabitol, D-cellobiose, D-fructose, D-galactose, gentiobiose, D-glucose, glycerol, glycogen, inositol, D-mannitol, D-mannose, N-acetylglucosamine, potassium gluconate, potassium 5-ketogluconate, L-rhamnose, D-ribose, starch (amidon), and D-trehalose. Positive for acid phosphatase, alkaline phosphatase, cystine arylamidase, α-fucosidase, α-galactosidase, ß-galactosidase, α-glucosidase, ß-glucosidase, leucine arylamidase, α-mannosidase, N-acetyl-ß-glucosaminidase, naphthol-AS-BI-phosphohydrolase, and valine arylamidase. Whole cell sugars consist of glucose, ribose, and mannose with traces of galactose. The peptidoglycan contains LL-A_2_pm. The fatty acids profile comprises (>5%) isoC_15:0_, *ante*isoC_15:0_, *ante*isoC_17:0_, isoC_17:0_, isoC_16:0_. Polar lipid pattern consists of diphosphatidylglycerol, phosphatidylinositol, phosphatidylethanolamine, unidentified phospholipids, aminolipid, and phosphoglycolipid. The genome size of strain DSM 41699^T^ is 10.2 Mb with DNA G+C content of 70.6%.

The type strain, DSM 41699^T^ (= ATB-26^T^ = KCTC 59183^T^) was isolated from soil sample collected in Tasek Bera, Malaysia. The GenBank accession number of the assembled draft genome is JAVREY000000000.1.

### Description of *Streptomyces salyersiae* sp. nov

*Streptomyces salyersiae* (*sa.ly.er'si.ae.* N.L. gen. fem. n. *salyersiae*, of Salyers, in honour of Abigail A. Salyers (1942–2013) known as the mother of microbiome research).

Gram-stain positive, aerobic actinobacterium with extensively branched substrate mycelium that develop grey aerial mycelium on ISP1, ISP2, ISP5, ISP6, ISP7, DSMZ 535, GYM, and NZ amine media. The strain grows well from 20 to 37 °C, optimally at 28 °C, from pH 5.5 to 8.0, optimally at pH 7.0. The strain is able to assimilate amygdalin, L-arabinose, D-arabitol, D-cellobiose, D-fructose, gentiobiose, D-glucose, glycerol, glycogen, D-maltose, D-mannitol, D-mannose, N-acetylglucosamine, potassium gluconate, L-rhamnose, D-ribose, starch (amidon), D-trehalose, D-turanose, and D-xylose. Positive for esterase lipase (C 8), ß-galactosidase, α-glucosidase, leucine arylamidase, and lipase (C 14). Whole cell sugars consist of glucose, ribose, rhamnose with traces of mannose. The peptidoglycan contains LL-A_2_pm and trace of DL-A_2_pm. The fatty acids profile comprises (>5%) *ante*isoC_15:0_, *anteiso*C_17:0_, C_16:0_, *iso*C_15:0_, *iso*C_16:0_. Polar lipid pattern consists of diphosphatidylglycerol, phosphatidylinositol, phosphatidylethanolamine, phospholipids, aminolipid, phosphoglycolipid. The genome size of strain DSM 41770^T^ is 11.2 Mb with DNA G+C content of 70.0%.

The type strain DSM 41770^T^ (= 157/96^T^ = KCTC 59182^T^) was isolated from water collected from a damaged gypsum liner in a children`s day care centre in Finland. The GenBank accession number of the assembled draft genome is JAVREX000000000.1.

### Description of *Streptomyces evansiae* sp. nov

*Streptomyces evansiae* (e.van'si.ae. N.L. gen. fem. n. *evansiae*, of Evans, in honour of Alice Catherine Evans, known for her great bacteriological work on milk and cheese and for discovering that *Bacillus abortus* caused Brucellosis in animals and humans, which resulted in the pasteurization of milk).

Gram-stain positive, aerobic actinobacterium with extensively branched substrate mycelium that develop white aerial mycelium on ISP3, ISP5, and DSMZ 535 media. The strain grows well from 25 °C to 37 °C, optimally at 28 °C, from pH 5.5 to 8.0, optimally at pH 7.0. The strain is able to assimilate amygdalin, D-arabitol, arbutin, D-cellobiose, D-fructose, L-fucose, D-galactose, D-glucose, glycerol, D-lactose (bovine origin), D-mannitol, D-mannose, methyl-α-D-glucopyranoside, methyl-ß-D-xylopyranoside, N-acetylglucosamine, potassium gluconate, L-rhamnose, salicin, D-trehalose, and D-xylose. Positive for acid phosphatase, alkaline phosphatase, α-chymotrypsin, esterase (C 4), esterase lipase (C 8), ß-galactosidase, α-glucosidase, ß-glucosidase, ß-glucuronidase, leucine arylamidase, lipase (C 14), naphthol-AS-BI-phosphohydrolase, and valine arylamidase.

Whole cell sugars consist of glucose, ribose, with traces of mannose and galactose. The peptidoglycan contains LL-A_2_pm and trace of LL-A_2_pm. The fatty acids profile comprises (>5%) *ante*isoC_15:0_, *iso*C_15:0_, C_16:0_, *iso*C_16:0_, *anteiso*C_17:0_. Polar lipid pattern consists of diphosphatidylglycerol, phosphatidylinositol, phosphatidylethanolamine, unidentified phospholipids, phosphoaminolipid, phosphoglycolipid. The genome size of strain DSM 41979^T^ is 7.5 Mb with DNA G+C content of 73.3%.

The type strain DSM 41979^T^ (= SA3-ActF^T^ = KCTC 59185^T^) was isolated from *Sirex noctilio* collected in the USA. The GenBank accession number of the assembled draft genome is JAVRET000000000.1.

### Description of *Streptomyces johnsoniae* sp. nov

*Streptomyces johnsoniae* (john.so'ni.ae. N.L. gen. fem. n. *johnsoniae*, of Johnson, in honour of Mattiedna Johnson (1918–2003) for her significant contributions in antibiotic research).

Gram-stain positive, aerobic actinobacterium with extensively branched substrate mycelium that develop a weak white aerial mycelium on ISP1 agar media that is absent in NZ amine medium. The strain grows from 20 to 45 °C, optimally at 28 °C, from pH 5.5 to 8.0, optimally at pH 7.0. The strain is positive for acid phosphatase, alkaline phosphatase, ß-galactosidase, α-glucosidase, ß-glucosidase, leucine arylamidase, α-mannosidase, and valine arylamidase. Whole cell sugars consist of glucose, ribose, with traces of mannose. The peptidoglycan contains LL-A_2_pm. The fatty acids profile comprises (>5%) *iso*C_16:0_, *iso*C_16:1_ cis9, *anteiso*C_17:0_, *anteiso*C_17:1_ cis9. Polar lipid pattern consists of diphosphatidylglycerol, phosphatidylethanolamine, phosphatidylinositol, unidentified phospholipids, and phosphoglycolipid. The genome size of strain DSM 41886^T^ is 7.5 Mb with DNA G+C content of 72.5%.

The type strain DSM 41886^T^ (= CNB 984^T^ = KCTC 59171^T^) was isolated from marine sediment collected in the USA and known as cyclomarin A producer. The GenBank accession number of the assembled draft genome is JAVREV000000000.1

### Description of *Streptomyces dubilierae* sp. nov

*Streptomyces dubilierae* (du.bi.li'er.ae. N.L. gen. fem. n. *dubilierae*, of Dubilier, in honour of Nicole Dubilier for her significant contributions to the ecological and evolutionary symbiosis of microbes).

Gram-stain positive, aerobic actinobacterium with extensively branched substrate mycelium that develop yellow grey aerial mycelium on ISP1 and ISP2 media that turn to have light brown colour on ISP3 and GYM media. The strain grows well from 25 to 50 °C, optimally at 28 °C, from pH 5.5 to 8.0, optimally at pH 7.0. The strain is able to assimilate D-adonitol, amygdalin, D- and L-arabinose, D-arabitol, arbutin, D-cellobiose, D-fructose, L-fucose, D-galactose, gentiobiose, D-glucose, glycerol, glycogen, inositol, D-maltose, D-mannitol, D-mannose, D-melibiose, methyl-α-D-glucopyranoside, methyl-α-D-mannopyranoside, N-acetylglucosamine, potassium gluconate, D-raffinose, L-rhamnose, D-ribose, D-saccharose (sucrose), salicin, starch (amidon), D-trehalose and D-xylose. Positive for acid phosphatase, alkaline phosphatase, esterase lipase (C 8), ß-galactosidase, α-glucosidase, ß-glucosidase, leucine arylamidase, lipase (C 14), N-acetyl-ß-glucosaminidase, naphthol-AS-BI-phosphohydrolase, and valine arylamidase. Whole cell sugars consist of glucose, ribose, with traces of mannose and galactose. The peptidoglycan contains LL-A_2_pm. The fatty acids profile comprises (>5%) *iso*C_14:0_, *iso*C_15:0_, *anteiso*C_15:0_, *anteiso*C_17:0_, C_16:0_, *iso*C_16:0_, C_16:1_ cis9. Polar lipid pattern consists of diphosphatidylglycerol, phosphatidylinositol, phosphatidylethanolamine, phospholipids, aminolipid, phosphoglycolipid. The genome size of strain DSM 41921^T^ is 8.2 Mb with DNA G+C content of 72.4%.

The type strain DSM 41921^T^ (= S6^T^ = KCTC 59180^T^) was isolated from rhizospherical soil of *Vitis vinifera*, collected in Morocco. The GenBank accession number of the assembled draft genome is JAVREU000000000.1.

### Description of *Streptomyces althioticus* subsp*. attaecolombicae* subsp*.* nov

*Streptomyces althioticus* subsp*. attaecolombicae* (at.tae.co.lom'bi.cae. N.L. gen, fem. n. *attaecolombicae,* of *Atta colombica,* the leafcutter ant species from which the strain was isolated).

Gram-stain positive, aerobic actinobacterium with extensively branched substrate mycelium with well-developed light grey (ISP1, ISP3 media), white (on ISP4, ISP5, ISP7 medium), light grey (ISP6), and yellow (DSMZ 535 and 65, NZ-amine media) aerial after 7 days of incubation at 28 °C. The strain grows well at 28 °C, 35 °C to 37 °C, optimally at 28 °C, from pH 5.0 to 9.0, optimally at pH 7.0. The strain is able to assimilate a wide range of carbon source such as glycerol, L-arabinose, D-xylose, D-glucose, D-fructose, D-mannose, L-rhamnose, inositol, D-mannitol, N-acetylglucosamine, esculin / ferric citrate, salicin, D-cellobiose, D-maltose, D-trehalose, starch (amidon), glycogen, gentiobiose, L-arabitol, and potassium gluconate. The strain has enzymatic profile contains alkaline phosphatase, esterase (C 4), lipase (C 14), esterase lipase (C 8), leucine arylamidase, valine arylamidase, cystine arylamidase, and trypsin. The whole cell sugars consist of glucose and ribose. The peptidoglycan contains LL-A_2_pm. The fatty acids profile comprises (>5%) *ante*isoC_15:0_, *anteiso*C_17:0_, C_16:0_, *iso*C_15:0_, *iso*C_16:0_, C_15:0_. Polar lipid pattern consists of diphosphatidylglycerol, phosphatidylinositol, phosphatidylethanolamine, phosphoglycolipid, unidentified aminolipid, phospholipid, and phosphoaminolipid. The genome size of strain DSM 41972^T^ is 6.64 Mb with DNA G+C content of 72.2%.

The type strain DSM 41972^T^ (= Av26–2^T^ = DI-188^T^ = KCTC 59178^T^) was isolated from *Atta colombica* refuse dump collected in Panama. The GenBank accession number of the assembled draft genome is JAVSGH000000000.

### Description of *Streptomyces doudnae* sp. nov

*Streptomyces doudnae* (do.ud'nae. N.L. gen. fem. N. *doudnae*, of Doudna, in honour of Jennifer A. Doudna for her great contributions in biochemistry, genetics, and microbiology. She had a Nobel Prize (2020) in Chemistry for her pioneering work in the field of CRISPR genome editing).

Gram-stain positive, aerobic actinobacterium with extensively branched substrate mycelium that develop white aerial mycelium on ISP2, TSA and NZ amine agar media that turn to have lavender colour on ISP3, ISP4, ISP5, ISP7, GYM media. The strain grows well from 25 to 45 °C, optimally at 28 °C, from pH 5.5 to 8.0, optimally at pH 7.0. The strain is able to assimilate L- and D-arabinose, D-arabitol, D-cellobiose, D-fructose, L-fucose, D-galactose, D-glucose, glycerol, glycogen, inositol, D-maltose, D-mannitol, D-mannose, N-acetylglucosamine, L-rhamnose, D-ribose, starch, D-trehalose, and D-xylose. Positive for acid phosphatase, alkaline phosphatase, cystine arylamidase, α-fucosidase, ß-galactosidase, α-glucosidase, ß-glucosidase, leucine arylamidase, lipase (C 14), α-mannosidase, naphthol-AS-BI-phosphohydrolase, and valine arylamidase. Whole cell sugars consist of glucose, ribose, and traces of mannose. The peptidoglycan contains LL-A_2_pm. The fatty acids profile comprises (>5%) *iso*C_14:0_, *iso*C_15:0_, *anteiso*C_15:0_, *anteiso*C_17:0_, C_16:0_, *iso*C_16:0_. Polar lipid pattern consists of diphosphatidylglycerol, phosphatidylinositol, phosphatidylethanolamine, phospholipids, phosphoaminolipid, phosphoglycolipids. The genome size is 9.2 Mb with DNA G+C content of 72.9%.

The type strain DSM 41981^T^ (= Sol5a-2^T^ = KCTC 59175^T^) was isolated from a solitary wasp collected in Panama. The GenBank accession number of the assembled draft genome is JAVRES000000000.1.

### Description of *Streptomyces hazeniae* sp. nov

*Streptomyces hazeniae* (ha.ze'ni.ae. N.L. gen. fem. n. *hazeniae,* of Hazen, in honour of Elizabeth Lee Hazen (1885–1975) for her great contributions to microbiology and the development of nystatin).

Gram-stain positive, aerobic actinobacterium with extensively branched substrate mycelium with no aerial mycelium on ISP1, ISP2, ISP3, ISP4, ISP5, ISP7, and GYM media. The strain grows well from 17°C to 37 °C, optimally at 28 °C, from pH 5.5 to 8.0, optimally at pH 7.0. The strain is able to assimilate D-arabitol, D-cellobiose, D-fructose, gentiobiose, D-glucose, glycogen, D-maltose, D-mannitol, D-mannose, D-melibiose, D-raffinose, D-ribose, D-saccharose (sucrose), starch (amidon), D-turanose, and D-xylose. Positive for acid phosphatase, alkaline phosphatase, esterase (C 4), esterase lipase (C 8), α-glucosidase, leucine arylamidase, α-mannosidase, N-acetyl-ß-glucosaminidase, naphthol-AS-BI-phosphohydrolase, and valine arylamidase. Whole cell sugars consist of glucose, ribose, galactose with trace of mannose. The peptidoglycan contains LL-A_2_pm. The fatty acids profile comprises (>5%) *anteiso*C_15:0_, *iso*C_15:0_, *anteiso*C_17:0_, *iso*C_16:0._ Polar lipid pattern consists of diphosphatidylglycerol, phosphatidylinositol, phosphatidylethanolamine, and unidentified phospholipids. The genome size of strain DSM 42041^T^ is 6.4 Mb with DNA G+C content of 72.8%.

The type strain DSM 42041^T^ (= SCSIO 10374^T^ = KCTC 59186^T^) was isolated from gorgonian coral in China. The GenBank accession number of the assembled draft genome is JAVREQ000000000.1.

### Description of *Streptomyces chisholmiae* sp. nov

*Streptomyces chisholmiae* (chis.hol'mi.ae. N.L. gen. fem. n. *chisholmiae*, of Chisholm, in honour of Sallie ‘’Penny’’ W. Chisholm for her significant contributions to the ecology and evolution of ocean microbes.).

Gram-stain positive, aerobic actinobacterium with branched substrate mycelium with white and lavender aerial mycelium on ISP1, DSMZ 535, and ISP3 media, respectively. Red brown pigment was diffused produced on ISP7 medium. The strain grows well from 20 to 42 °C, optimally at 28 °C, from pH 5.5 to 8.0, optimally at pH 7.0. The strain is able to assimilate D-adonitol, amygdalin, L-arabinose, D-arabitol D-cellobiose, D-galactose, D-glucose, glycerol, D-lactose (bovine origin), D-maltose, D-mannitol, D-mannose, N-acetylglucosamine, L-rhamnose, D-ribose, starch (amidon), D-trehalose, and D-xylose. Positive for alkaline phosphatase, esterase lipase (C 8), leucine arylamidase, naphthol-AS-BI-phosphohydrolase, trypsin, and valine arylamidase. Whole cell sugars consist of glucose, ribose, with traces of mannose. The peptidoglycan contains LL-A_2_pm. The fatty acids profile comprises (>5%) *anteiso*C_17:0_, *iso*C_16:0_, *iso*C_16:1_ cis4, C_15:0_. Polar lipid pattern consists of diphosphatidylglycerol, phosphatidylinositol, phosphatidylethanolamine, phospholipids, lipid, phosphoglycolipid. The genome size of strain DSM 44915^T^ is 7.5 Mb with DNA G+C content of 73.7%.

The type strain DSM 44915^T^ (= SCRIPP CNJ 962^T^ = KCTC 59194^T^) was isolated from marine sediment collected in Palau. The GenBank accession number of the assembled draft genome is JAVREO000000000.1

### Description of *Streptomyces boetiae* sp. nov

*Streptomyces boetiae* (bo.e'ti.ae. N.L. gen. fem. n. *boetiae*, of Boetius, in honour of Antje Boetius for her outstanding contributions to marine microbiology, formed under the assumption that Boetius is a Latin or Latinized name).

Gram-stain positive, aerobic actinobacterium with branched substrate mycelium with white and lavender aerial mycelium on ISP1, ISP2, TSBA and ISP3, ISP4, ISP7, and GYM media, respectively. White griseus aerial mycelium was developed on NZ amine agar medium. No diffusible pigment was produced. The strain grows well from 20 to 42 °C, optimally at 28 °C, from pH 5.5 to 8.0, optimally at pH 7.0. The strain is able to assimilate D-fructose, glycerol, glycogen, potassium gluconate, and D-trehalose. Positive for alkaline phosphatase, cystine arylamidase, esterase (C 4), esterase lipase (C 8), α-glucosidase, leucine arylamidase, lipase (C 14), and valine arylamidase. Whole cell sugars consist of glucose and ribose with traces of mannose. The peptidoglycan contains LL-A_2_pm. The fatty acids profile comprises (>5%) *anteiso*C_15:0_, *iso*C_16:1_ cis9, *anteiso*C_17:0_, *iso*C_16:0._ Polar lipid pattern consists of diphosphatidylglycerol, phosphatidylinositol, phosphatidylethanolamine, phosphatidylmethylethanolamine, unidentified phospholipids, phosphoglycolipid and phosphoaminolipid. The genome size of strain DSM 44917^T^ is 6.0 Mb with DNA G+C content of 74.7%.

The type strain DSM 44917^T^ (= SCRIPP CNQ 259^T^ = KCTC 59173^T^) was isolated from marine sediment collected in the USA. The GenBank accession number of the assembled draft genome is JAVREN000000000.1.

### Description of *Streptomyces millisiae* sp. nov

*Streptomyces millisiae* (mil.li'si.ae. N.L. gen. fem. n, *millisiae.* of Millis, in honour of Nancy F. Millis (1922–2012) who introduced applied microbiology and promoted wastewater microbiology in Australia).

Gram-stain positive, aerobic actinobacterium with branched substrate mycelium with grey brownish to brown colour on ISP2 and ISP3 media, respectively. Red brown pigment was diffused produced on ISP7 medium. The strain grows well from 25 to 45 °C, optimally at 28 °C, from pH 5.5 to 8.0, optimally at pH 7.0. The strain is able to assimilate D-adonitol, arbutin, D-maltose, D-mannitol, D-melibiose, methyl-α-D-glucopyranoside, L-rhamnose, salicin, and D-trehalose. Positive for acid phosphatase, alkaline phosphatase, α-galactosidase, ß-galactosidase, α-glucosidase, ß-glucosidase, leucine arylamidase, α-mannosidase, naphthol-AS-BI-phosphohydrolase, trypsin, and valine arylamidase. Whole cell sugars consist of glucose, ribose, with traces of mannose and galactose. The peptidoglycan contains LL-A_2_pm. The fatty acids profile comprises (>5%) *anteiso*C_15:0_, C_16:1_ cis9, C_16:0_, *anteiso*C_17:0_, *ante*isoC_17:1_ cis9, *iso*C_16:0_. Polar liprikhoid pattern consists of diphosphatidylglycerol, phosphatidylinositol, phosphatidylethanolamine, phosphatidylmethylethanolamine, unidentified phospholipids, and phosphoglycolipid. The genome size of strain DSM 44918^T^ is 7.6 Mb with DNA G+C content of 72.4%.

The type strain DSM 44918^T^ (= SCRIPP CNQ 703^T^ = KCTC 59174^T^) was isolated from marine sediment collected in Guam, USA. The GenBank accession number of the assembled draft genome is JAVREM000000000.1

### Description of *Streptomyces litchfieldiae* sp. nov

*Streptomyces litchfieldiae* (*litch.fiel'di.ae.* N.L. gen. fem. n. *litchfieldiae,* of Litchfield, in honour of Carol Ann Darlene Litchfield (1936–2012) for her great contributions to the microbiology of oceans and extreme environments).

Gram-stain positive, aerobic actinobacterium with branched substrate mycelium with lavender aerial mycelium on ISP3, ISP4, ISP5, ISP6, ISP7, and GYM media. White aerial mycelium develops on ISP2 and TSBA media. No diffusible pigment was produced on the tested media. The strain grows well from 20 to 37 °C, optimally at 28 °C, from pH 5.5 to 8.0, optimally at pH 7.0. The strain is able to assimilate D-adonitol, D- and L-arabinose, D-arabitol, D-cellobiose, D-fructose, L-fucose, D-galactose, gentiobiose, D-glucose, glycerol, glycogen, inositol, D-lactose (bovine origin), D-maltose, D-mannitol, D-mannose, D-melibiose, N-acetylglucosamine, potassium gluconate, D-raffinose, L-rhamnose, D-saccharose (sucrose), salicin, starch (amidon), D-trehalose, and D-xylose. Positive for acid phosphatase, alkaline phosphatase, α-chymotrypsin, cystine arylamidase, esterase (C 4), esterase lipase (C 8), α-fucosidase, α-galactosidase, ß-galactosidase, α-glucosidase, ß-glucosidase, leucine arylamidase, α-mannosidase, N-acetyl-ß-glucosaminidase, naphthol-AS-BI-phosphohydrolase, trypsin, and valine arylamidase. Whole cell sugars consist of glucose, ribose, with traces of mannose and galactose. The peptidoglycan contains LL-A_2_pm. The fatty acids profile comprises (>5%) *anteiso*C_15:0_, *anteiso*C_17:0_, *anteiso*C_17:1_ cis9, *iso*C_16:1_ cis9, *iso*C_16:0_. Polar lipid pattern consists of diphosphatidylglycerol, phosphatidylinositol, phosphatidylethanolamine, unidentified lipid, phospholipid, aminolipid, phosphoglycolipid. The genome size of strain DSM 44938^T^ is 7.6 Mb with DNA G+C content of 72.4%.

The type strain DSM 44938^T^ (= SCRIPP CNR954^T^ = KCTC 59172^T^) was isolated from marine sediment collected in Bahamas. The GenBank accession number of the assembled draft genome is JAVREL000000000.1.

### Description of *Blastococcus goldschmidtiae* sp. nov

*Blastococcus goldschmidtiae* (gold.schmid'ti.ae. N.L. gen. fem. n. *goldschmidtiae*, of Goldschmidt, in honour of Millicent C. Goldschmidt for her great contributions in the field of microbiology in particular in rapid testing methods for detecting microbial contaminants).

Gram-stain positive, aerobic actinobacterium with rod-, coccoid and non-motile cells forming orange colonies on ISP2, TSA, and GYM media. The strain grows well from 25 to 37 °C, optimally at 28 °C, from pH 5.5 to 8.0, optimally at pH 7.0. Positive for acid phosphatase, alkaline phosphatase, α-chymotrypsin, cystine arylamidase, esterase (C 4), esterase lipase (C 8), ß-glucosidase, leucine arylamidase, naphthol-AS-BI-phosphohydrolase, trypsin, and valine arylamidase. Whole cell sugars consist of glucose, mannose, and ribose. The fatty acids profile comprises (>5%) *iso*C_16:1_ cis9, *iso*C_16:0_, C_17:1_ cis9, C_18:1_ cis9. Polar lipid pattern consists of diphosphatidylglycerol, phosphatidylcholine, phosphatidylinositol, unidentified lipid, glycolipids. The genome size of strain DSM 46792^T^ is 4.3 Mb with DNA G+C content of 73.3%.

The type strain DSM 46792^T^ (= 7C^T^ = BC543^T^ = KCTC 59190^T^) was isolated from marble collected in Italy. The GenBank accession number of the assembled draft genome is JAVREI000000000.1.

### Description of *Nocardiopsis lambiniae* sp. nov

*Nocardiopsis lambiniae* (lam.bi'ni.ae. N.L. gen. fem. n. *lambiniae,* of Lambin, in honour of Suzanne Lambin (1902–2008) for her great work on the evolution of bacterial cultures).

Gram-stain positive, aerobic actinobacterium with branched substrate mycelium with light brown and orange colonies on ISP1 and ISP2 media, respectively. White aerial mycelium develops on ISP6, TSBA, and DSMZ 83 media. No diffusible pigment was produced on the tested media. The strain grows well from 20 to 37 °C, optimally at 28 °C, from pH 5.5 to 8.0, optimally at pH 7.0. Positive for acid phosphatase, alkaline phosphatase, α-chymotrypsin, esterase (C 4), esterase lipase (C 8), α-glucosidase, ß-glucosidase, leucine arylamidase, lipase (C 14), N-acetyl-ß-glucosaminidase, and valine arylamidase. Whole cell sugars consist of ribose. The peptidoglycan contains DL*-*A_2_pm. The fatty acids profile comprises (>5%) *anteiso*C_15:0_, *iso*C_16:0_, *anteiso*C_17:0_, C_17:1_ cis9, C_18:1_ cis9, C_18:2_ cis9,12. Polar lipid pattern consists of phosphatidylcholine, phosphtidylglycerol, phosphatidylmethylethanolamine, phospholipids. The genome size of strain DSM 44743^T^ is 6.2 Mb with DNA G+C content of 71.3%.

The type strain DSM 44743^T^ (= DCDM15A35^T^ = KCTC 59192^T^) was isolated from soil collected in China. The GenBank accession number of the assembled draft genome is JAVREP000000000.1.

### Description of *Pseudonocardia charpentierae* sp. nov

*Pseudonocardia charpentierae* (char.pen'tie.rae. N.L. gen. fem. n. *charpentierae*, of Charpentier, in honour of Emmanuelle Charpentier for her great contributions to microbiology, genetics and biochemistry. She had a Nobel Prize (2020) in Chemistry for her pioneering work in the field of CRISPR genome editing).

Gram-stain positive, aerobic non-motile actinobacterium with orange colonies that shows a good growth on GYM media. The strain grows well from 25 to 37 °C, optimally at 28 °C, from pH 5.5 to 8.0, optimally at pH 7.0. The strain is able to assimilate D- and L-arabitol, gentiobiose, glycogen, D-lyxose, N-acetylglucosamine, D-melibiose, potassium gluconate, D-saccharose (sucrose), starch (amidon), and D-turanose. Positive for acid phosphatase, esterase (C 4), esterase lipase (C 8), α-galactosidase, ß-galactosidase, α-glucosidase, ß-glucosidase, leucine arylamidase, naphthol-AS-BI-phosphohydrolase, and valine arylamidase. Whole cell sugars consist of arabinose, galactose, glucose, mannose and ribose. The peptidoglycan contains DL-A_2_pm. The fatty acids profile comprises (>5%) C_16:0_, *iso*C_16:0_, C_16:1_ cis9, C_16:0_ 10 methyl. Polar lipid pattern consists of diphosphatidylglycerol, phosphatidylcholine, phosphatidylinositol, phosphatidylethanolamine, phsophatidylmethylethanolamine, unidentified glycolipid, glycophospholipid, phospholipids, phosphoglycoaminolipid. The genome size of strain DSM 45834^T^ is 6.7 Mb with DNA G+C content of 71.9%.

The type strain DSM 45834^T^ (= CPCC 203558^T^ = KCTC 59187^T^) was isolated from a desert sand, collected in China. The GenBank accession number of the assembled draft genome is JAVREJ000000000.1.

### Description of *Streptomonospora wellingtoniae* sp. nov

*Streptomonospora wellingtoniae* (wel.ling.to'ni.ae. N.L. gen. fem. n. of *wellingtoniae,* in honour of Elizabeth Wellington for her great contributions to microbiomes and environmental microbiology).

Gram-stain positive, aerobic actinobacterium with white beige colonies that develop on ISP1 and nutrient agar media. Weak growth without aerial mycelium of the strain on ISP2, ISP3, and ISP4. The strain grows well from 25 to 37 °C, optimally at 28 °C, from pH 5.5 to 8.0, optimally at pH 7.0. Whole cell sugars consist of glucose, galactose, and ribose with traces of mannose. The peptidoglycan contains DL-A_2_pm. The fatty acids profile comprises (>5%) *iso*C_16:1_ cis4, *iso*C_16:0_, C_16:1_ cis9, C_16:0_, *anteiso*C_17:0_, TSBA C_18:0_ 10 methyl. Polar lipid pattern consists of diphosphatidylglycerol, phosphatidylglycerol, phosphatidylinositol, phosphatidylcholine, glycolipids, phospholipids, and phosphoglycolipid. The genome size of strain DSM 45055^T^ is 5.5 Mb with DNA G+C content of 72.6%.

The type strain DSM 45055^T^ (= CNQ 327^T^ = KCTC 59191^T^) was isolated from marine sediment collected in the USA. The GenBank accession number of the assembled draft genome is JAVREK000000000.1.

### Description of *Jatrophihabitans lederbergiae* sp. nov

*Jatrophihabitans lederbergiae* (le.der.ber'gi.ae. N.L. gen. fem. n. *lederbergiae*, of Lederberg, named in honour of Esther Miriam Zimmer Lederberg (1922–2006) for her great contributions to bacterial genetics and molecular biology).

Gram-stain positive, aerobic actinobacterium with extensively branched substrate mycelium that develop white aerial mycelium on ISP2, TSA, and NZ amine agar media that turn to have lavender colour on ISP3, ISP4, ISP5, ISP7, and GYM media. The strain grows well from 25 to 45 °C, optimally at 28 °C, from pH 5.5 to 8.0, optimally at pH 7.0. The enzymatic profile of the strain consists of leucine arylamidase, valine arylamidase, cystine arylamidase, acid phosphatase, naphthol-AS-BI-phosphohydrolase, ß-galactosidase, α-glucosidase, and ß-glucosidase. Whole cell sugars consist of glucose, ribose, rhamnose with traces of mannose and galactose. The peptidoglycan contains DL-A_2_pm. The fatty acids profile comprises (>5%) *iso*C_16:1_ cis9, *iso*C_16:0_, C_17:1_ cis9, C_17:0_ 10 methyl, C_18:1_ cis9, TBSA C_18:0_ 10 methyl. Polar lipid pattern consists of diphosphatidylglycerol, phosphatidylinositol, phosphatidylmethylethanolamine, glycoaminophosphatidylinositol, glycophospholipid, aminolipid, phosphoaminolipid, and phospholipids. The genome size of strain DSM 44399^T^ is 5.2 Mb with DNA G+C content of 68.2%.

The type strain DSM 44399^T^ (= AA-499^T^ = IFAM AA-499^T^) was isolated from sandstone of Linnaeus Terrace (1600 m), Antarctica. The GenBank accession number of the assembled draft genome is JAVREH000000000.1.

### Emended description of *Streptomyces asiaticus* Sembiring et al. 2001

The description is as stated by Sembiring et al. 2001 with the following modification.

The strain degrades adenine and pectin and grows at 10°C and 45°C. The type strain A14P1^T^ (= DSM 41761^T^ = JCM 11443^T^ = NBRC 100774^T^ = NCIMB 13675^T^) has a genome size 11.9 Mb with G+C content of 71.0%. The genome sequence is available in the GenBank under accession number NZ_JAGSHX000000000.1.

## Author contributions

Phenotypic, genetic, genomic and phylogenetic analyses were carried out by IN, GP, MJ, CS, YM. Chemotaxonomic analyses were performed by MNS, SK, GP, IN. Genome assemblies were curated by IM and JPG-E. *In silico* and *in vitro* evaluation of the microbial potential of the strains were carried out by AZ, OH, MD, JPG-E, RM, YM. Cluster networking was performed by JB, UN. RM carried out genetic manipulation of strains and chemical analysis thereof. The manuscript was written by IN and YM. All authors read and approved the final version of the manuscript after contributing on previous versions.

## Declaration of competing interest

The authors declare that they have no known competing financial interests or personal relationships that could have appeared to influence the work reported in this paper.

## Data Availability

The genome sequences of the 36 studied strains have been deposited at DDBJ/ENA/GenBank and the accessions numbers are provided in the manuscript (Table S1).

## Appendix Supplementary materials

Image, application 1

Image, application 2

Image, application 3

Image, application 4
