# Supplementary material for: Biotechnological and pharmaceutical potential of twenty-eight novel type strains of Actinomycetes from different environments worldwide
Source: Curr Res Microb Sci. 2024 Oct 11;7:100290. doi: 10.1016/j.crmicr.2024.100290 (PMC11533595; doi:10.1016/j.crmicr.2024.100290)
Supplement: Supplementary file 1 [file mmc1.docx]

**Biotechnological and pharmaceutical potential of twenty-eight novel type strains of *Actinomycetes* from different environments worldwide.**

Imen Nouioui^1^*, Judith Boldt^1,2^, Alina Zimmermann^1,2^, Roman Makitrynskyy^1^, Gabriele Pötter^1^, Marlen Jando^1^, Meike Döppner^1^, Sarah Kirstein^1^, Meina Neumann-Schaal^1,3^, Juan Pablo Gomez-Escribano^1^, Ulrich Nübel^1,2,3,4^, Yvonne Mast^1,2,3,4^*

^1^Leibniz-Institut DSMZ – German Collection of Microorganisms and Cell Cultures, Inhoffenstraße 7B, 38124 Braunschweig, Germany

^2^German Center for Infection Research (DZIF) Braunschweig, Germany

^3^Braunschweig Integrated Centre of Systems Biology (BRICS), Rebenring 56, 38106 Braunschweig, Germany

^4^Technische Universität Braunschweig, Institut für Mikrobiologie, Rebenring 56, 38106 Braunschweig, Germany

*Correspondence:

Prof. Dr. Yvonne Mast

yvonne.mast@dsmz.de

Dr. Imen Nouioui

Imen.nouioui@dsmz.de


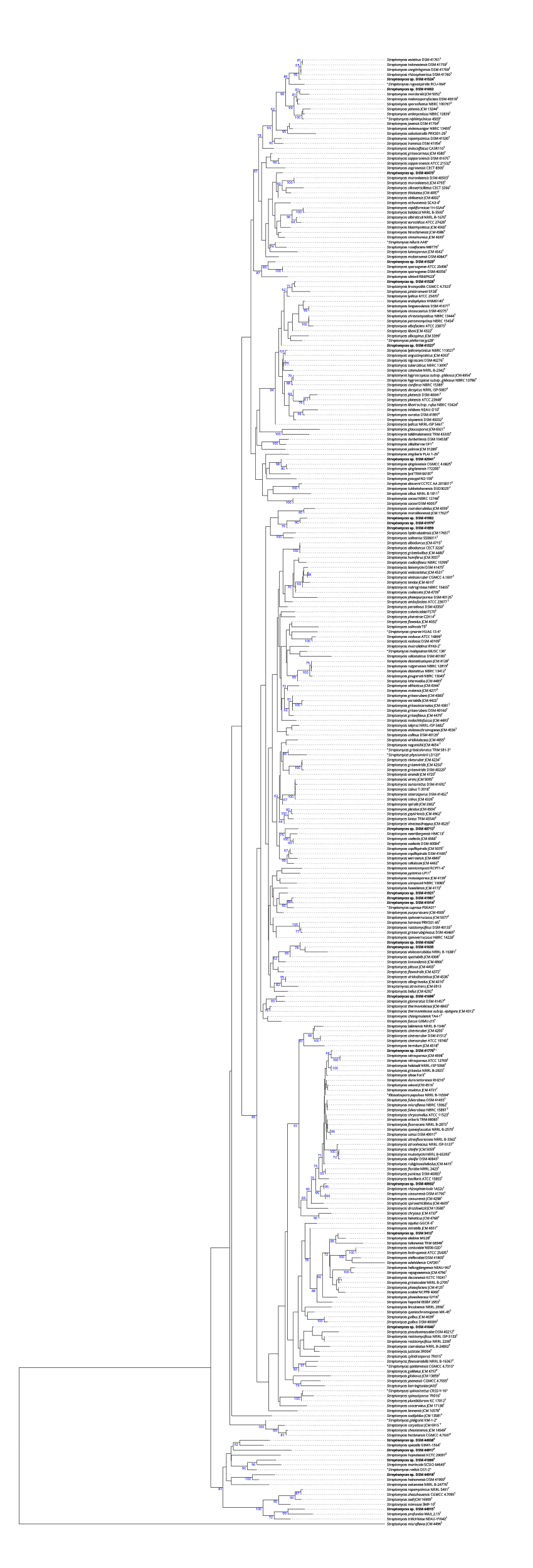


**Figure S1.** Maximum likelihood phylogenetic tree of the *Streptomyces* strains based on 16S rRNA gene sequences, showing the relationships between the strains and their closely related species. The numbers above branches are bootstrap value above 60.


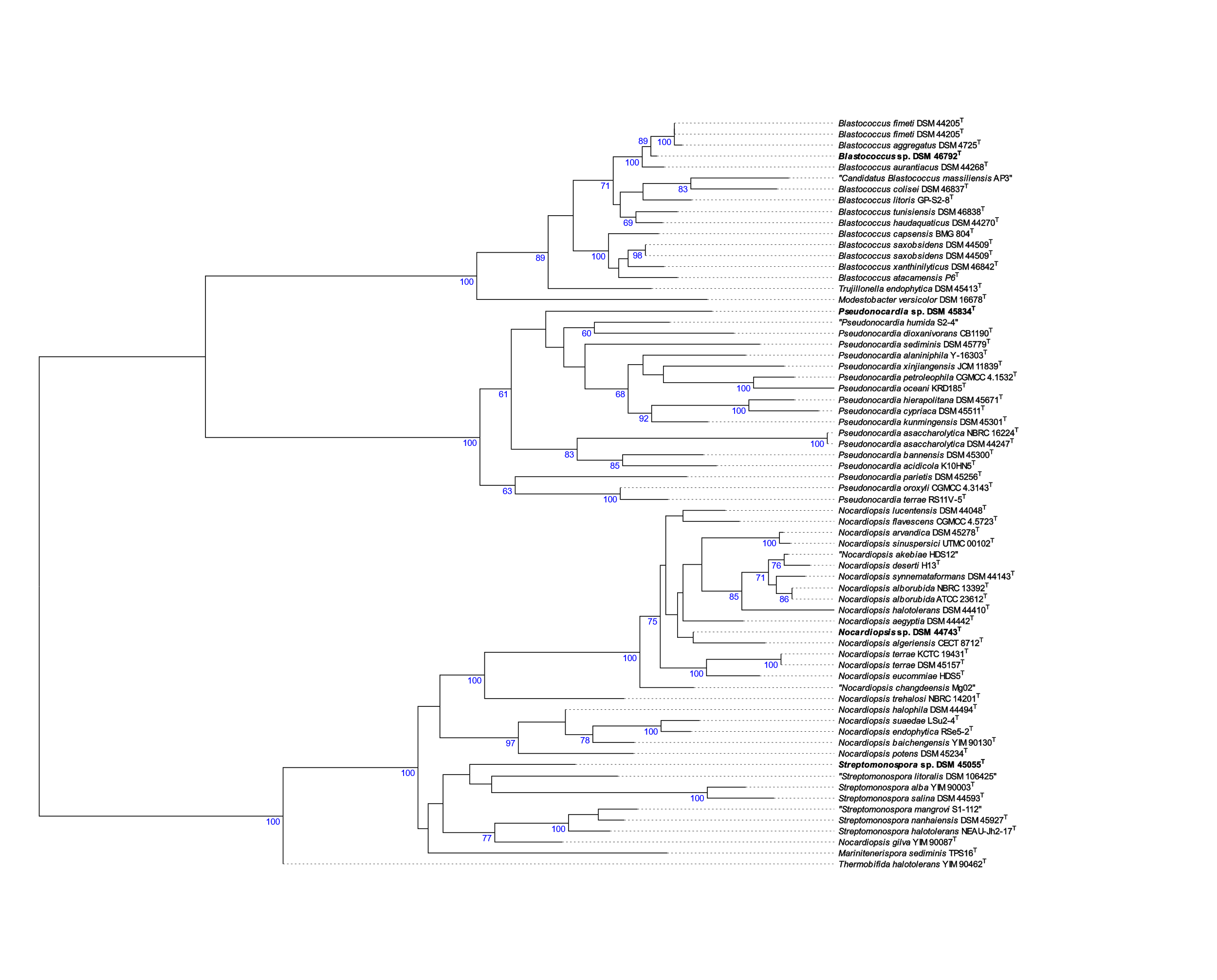


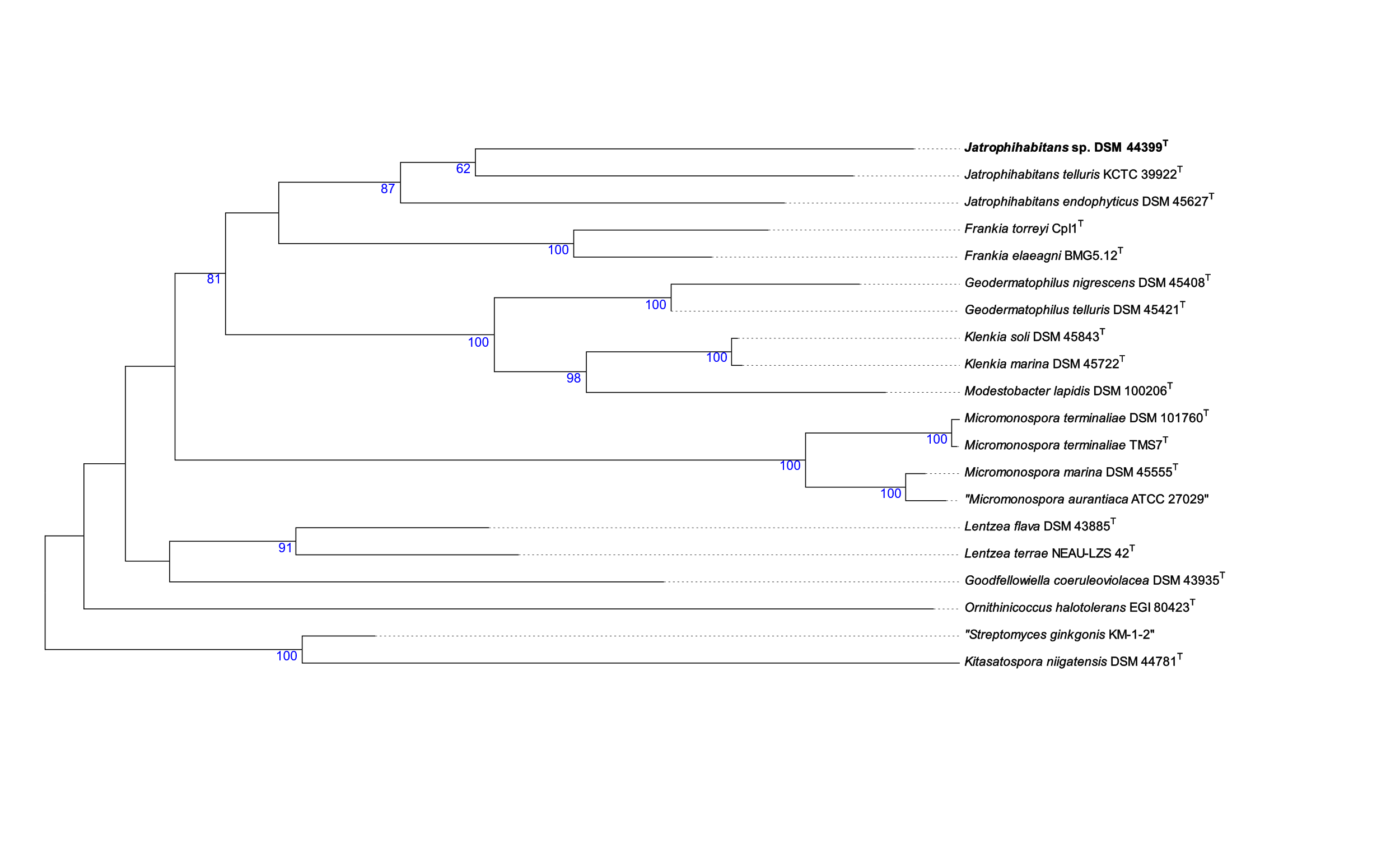


**Figure S2.** Maximum likelihood phylogenetic tree of the actinobacterial strains based on 16S rRNA gene sequences, showing the relationships between the strains and their closely related species. The numbers above branches are bootstrap value above 60.

**Figure S3.** Morphological features of the strains after incubation in an optimal growth conditions.


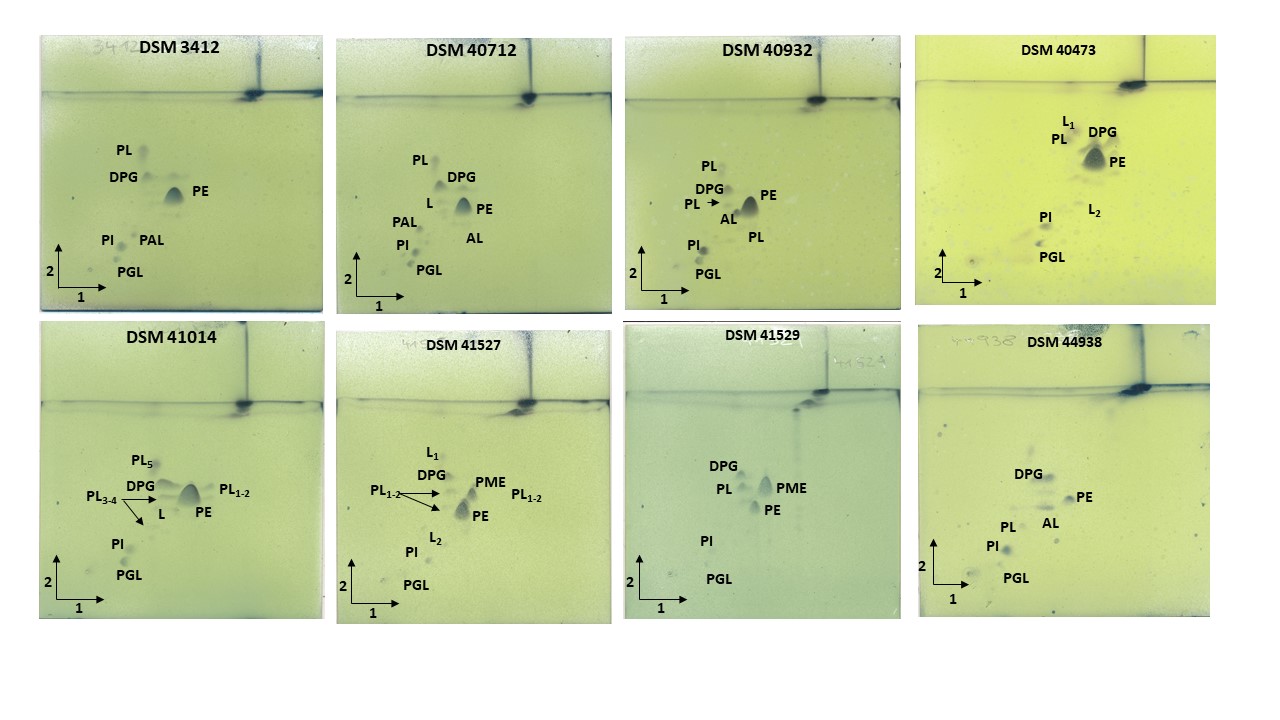


**
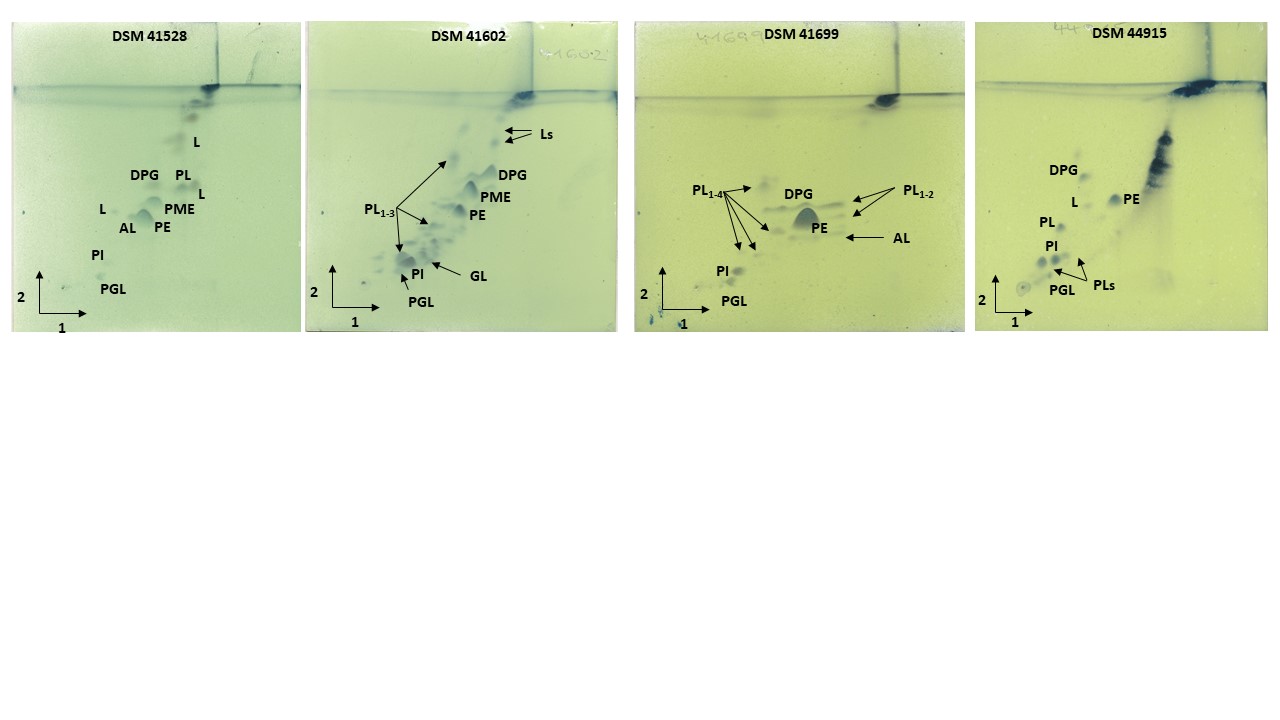

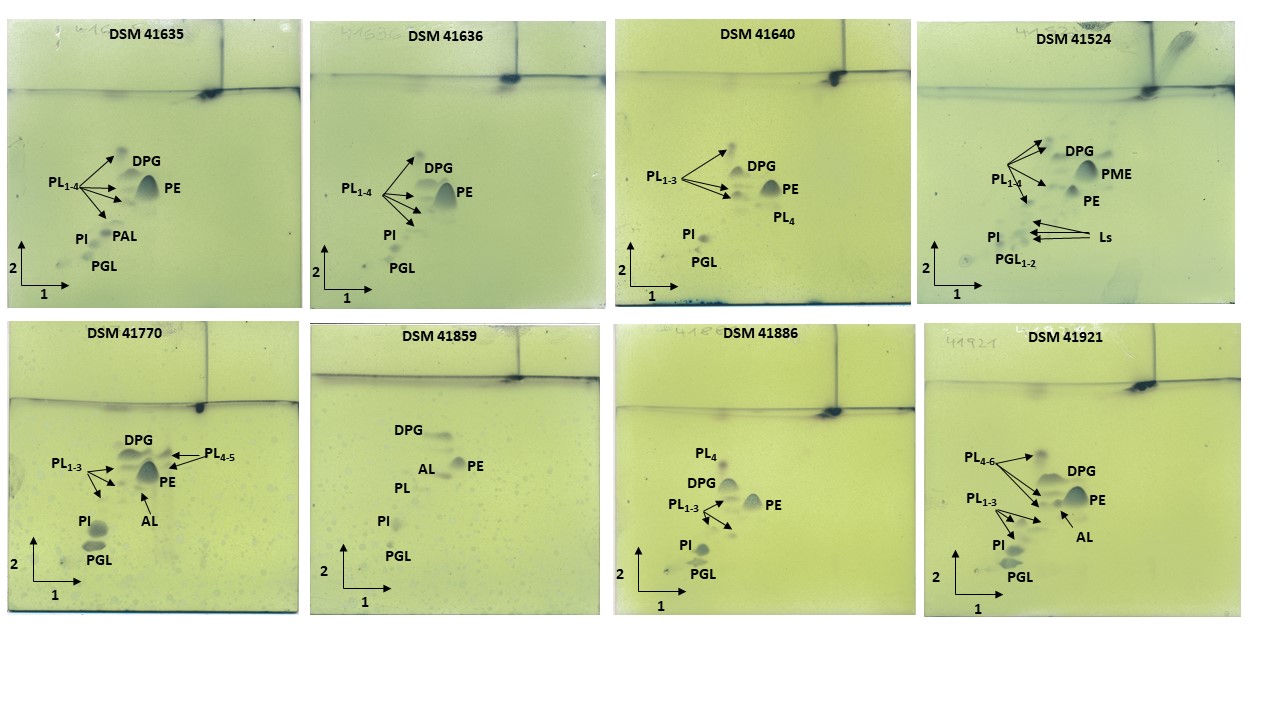
**

**
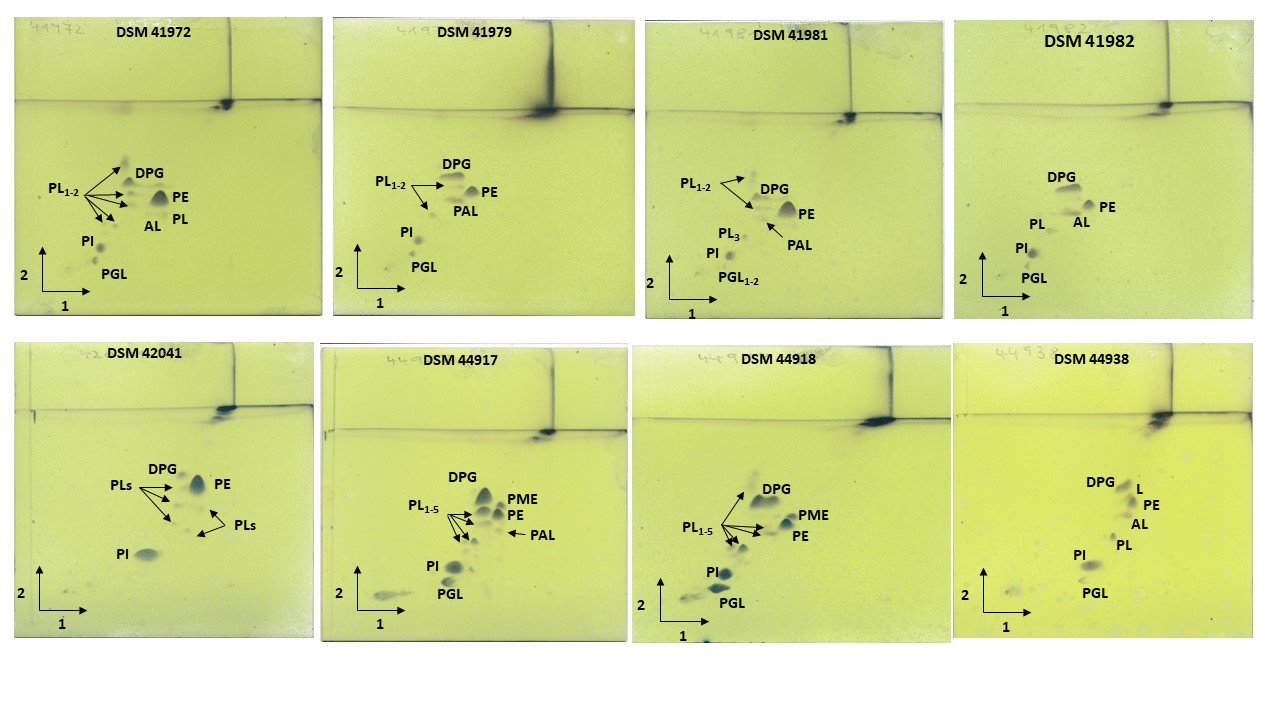
**

**
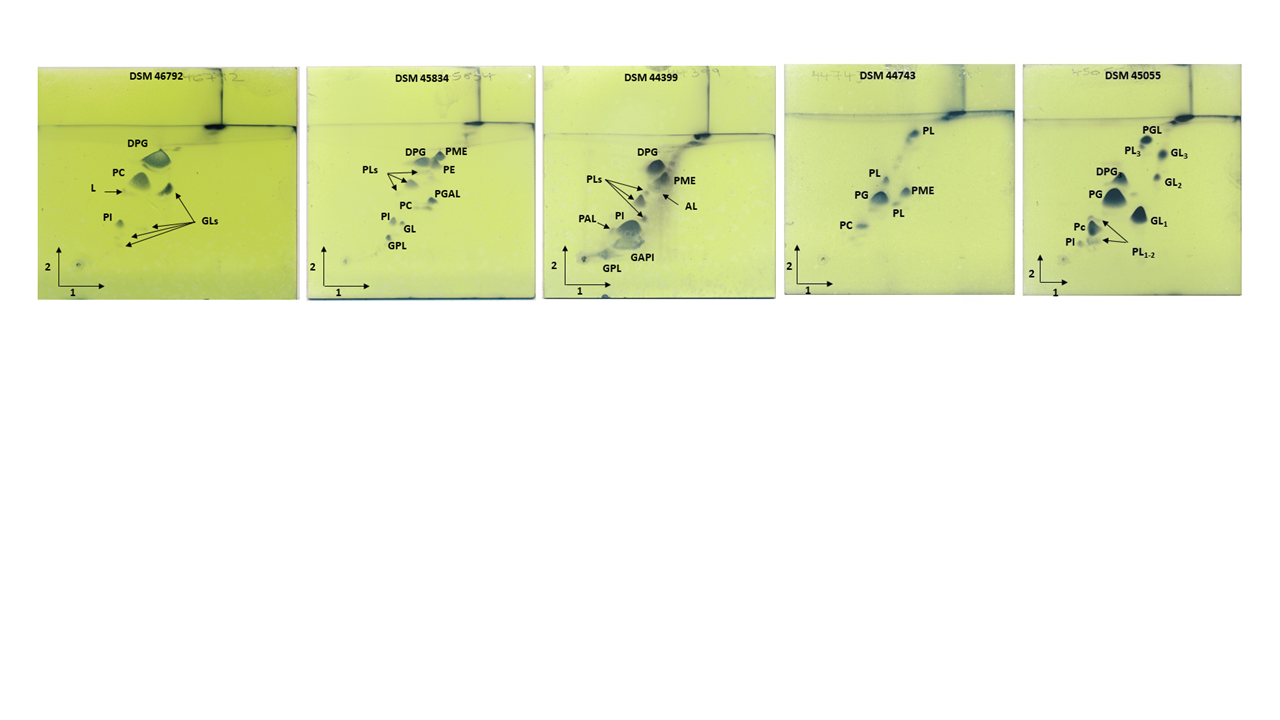
**

**Figure S4.** Two-dimensional TLC plate of polar lipids extracted from the studied strains stained with molybdatophosphoric acid (SigmaP1518). Key: DPG, diphosphatidylglycerol; PI, phosphatidylinositol; PGL; phosphoglycolipid; PL, phospholipid; L, lipid; PC phosphatidylcholine; PME phosphatidylmethylethanolamine; GL glycolipid; PG; phosphatidylglycerol; PGL phosphoglycolipid: PAL phosphoaminolipid: AL aminolipid. Solvent1: chloroform: methanol: distilled water (65:25:4 v/v/v/); solvent 2: chloroform: glacial acetic acid: methanol: distilled water (80:12:15:4 v/v/v).

A)


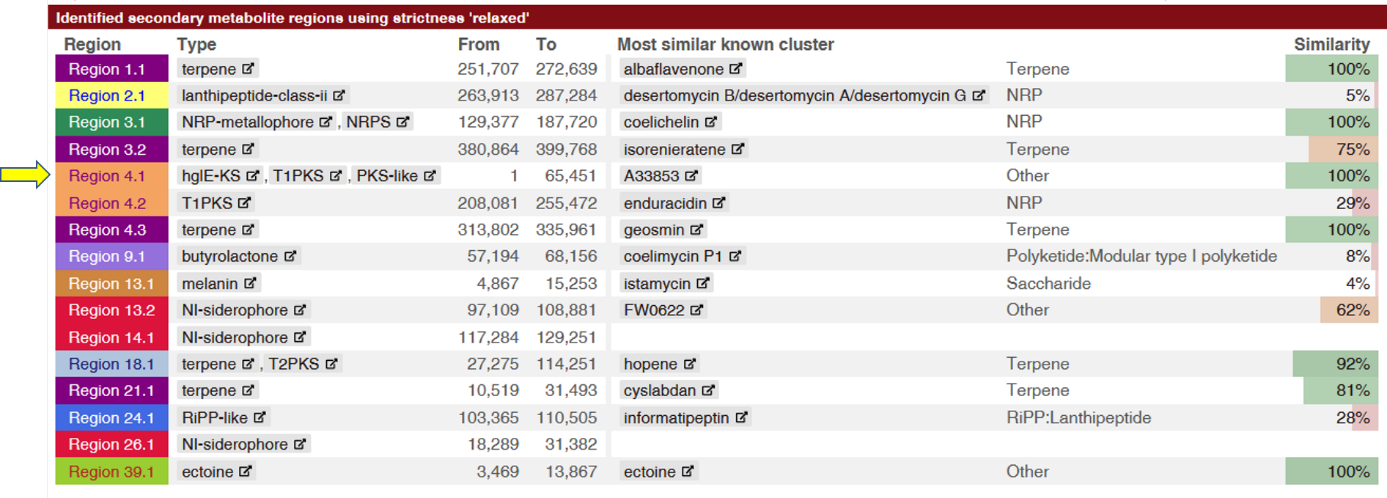


B)


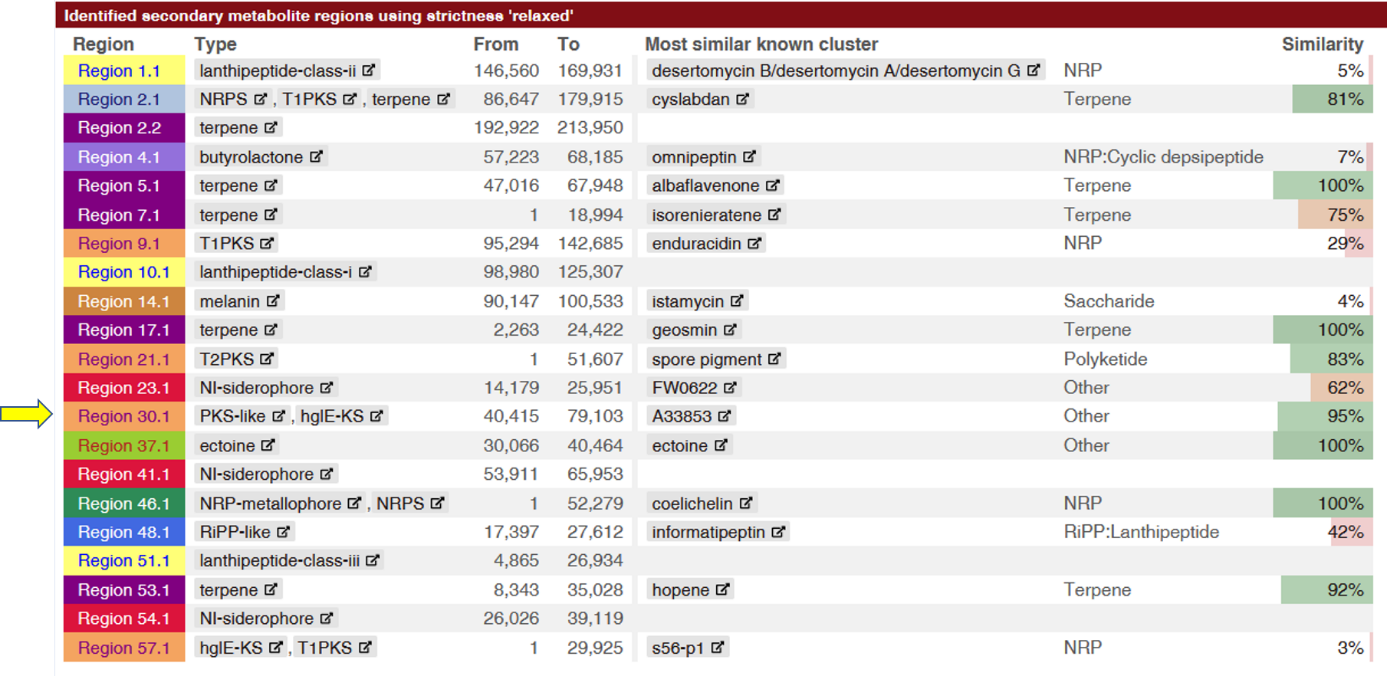


C)


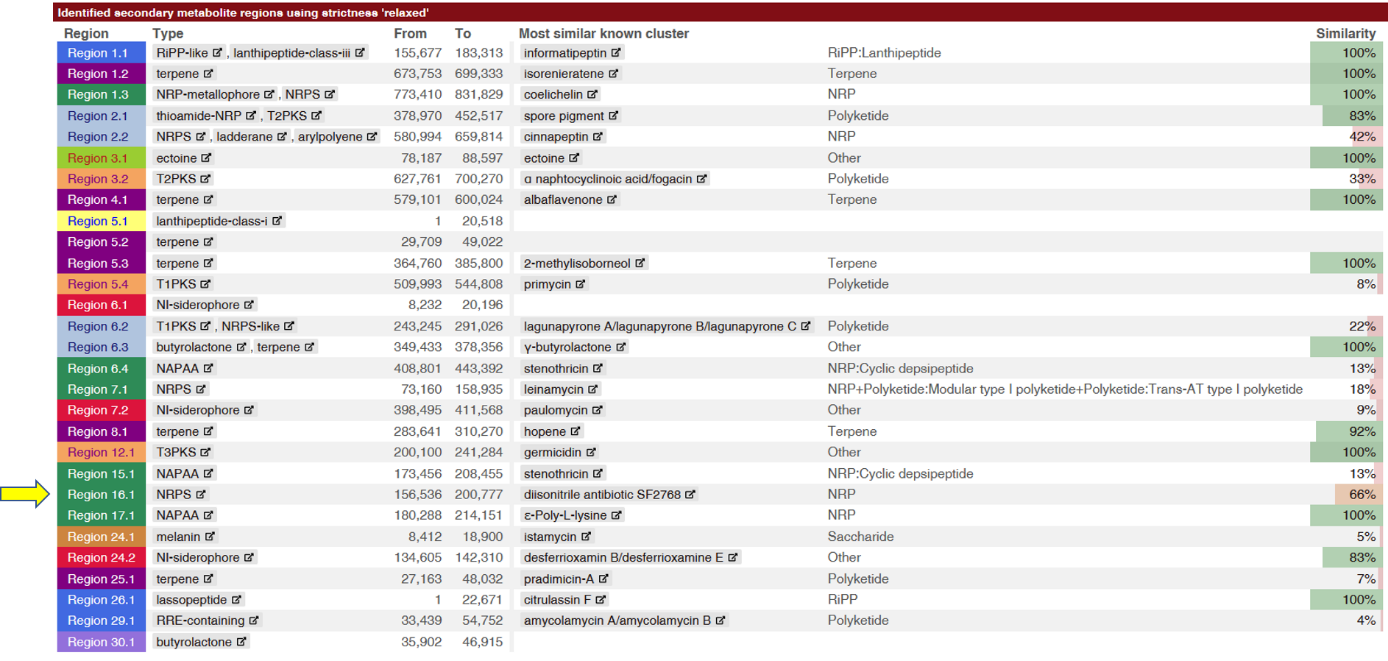


**Figure S5:** AntiSMASH output for strains (A) DSM 41635, (B) DSM 41636^T^, and (C) DSM 41640^T^. Candidate BGCs are highlighted by a yellow arrow.

**
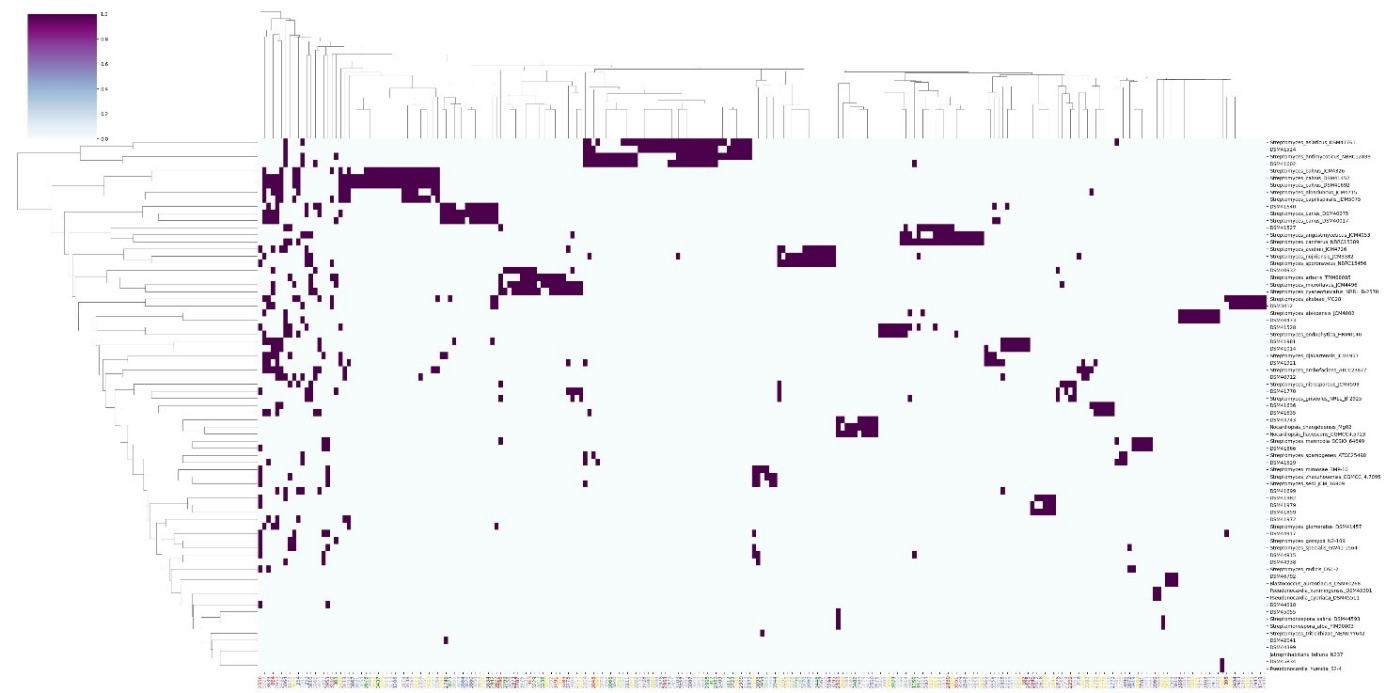
**

**Figure S6.** Presence/absence map and hierarchical clustering showing the relatedness of the strains and their closest neighbours based on the GCFs predicted with BiG-SCAPE. Dark blue indicates the presence of a GCF, light blue the absence. Only BGCs within contigs were considered here. Singletons are excluded in this graphic.

**Table S1.** Genomic features of the strains and their closest phylogenomic relatives

| **Strains** | **Genome size [Mb]** | **GC percent** | **Contig N50** | **Genes (RNA)** | **CDSs (total)** | **NCBI nucelotide accession number** | **NCBI assembly accession number** |
| --- | --- | --- | --- | --- | --- | --- | --- |
| DSM 41886 | 7,5 | 72,5 | 373.6 kb | 76 | 6644 | JAVREV000000000 | GCF_031845565.1 |
| *Streptomyces marincola* SCSIO 64649^T^ | 6,6 | 73,5 | 6.6 Mb | 74 | 5680 | NZ_CP084541.1 | GCF_020410765.1 |
| DSM 44938 | 7,7 | 72,5 | 288.2 kb | 69 | 7112 | JAVREL000000000 | GCF_031845365.1 |
| *Streptomyces radicis* DS1-2^T^ | 7,6 | 73 | 225.2 kb | 72 | 6392 | NZ_RBDY00000000.1 | GCF_003626575.1 |
| DSM 44917 | 6,1 | 74,5 | 108.3 kb | 78 | 5477 | JAVREN000000000 | GCF_031845405.1 |
| *Streptomyces specialis* GW41-1564^T^ | 5,9 | 72,5 | 185.2 kb | 68 | 5462 | NZ_FAXE00000000.1 | GCF_001493375.1 |
| DSM 44918 | 8 | 73 | 98.8 kb | 77 | 7430 | JAVREM000000000 | GCF_031845425.1 |
| *Streptomyces hainanensis* DSM 41900^T^ | 8 | 73 | 13.7 kb | 70 | 7384 | NZ_SMKI00000000.1 | GCF_004348415.1 |
| DSM 44915 | 7,5 | 73,5 | 309.2 kb | 71 | 6619 | JAVREO000000000 | GCF_031845445.1 |
| *Streptomyces zhaozhouensis* CGMCC 4.7095^T^ | 6,5 | 73,5 | 234.5 kb | 69 | 5557 | NZ_OCNE00000000.1 | GCF_900230195.1 |
| *Streptomyces sedi* JCM 16909^T^ | 6,6 | 73 | 245 kb | 66 | 5571 | NZ_VDGT00000000.1 | GCF_006335015.1 |
| *Streptomyces mimosae* 3MP-10^T^ | 7,2 | 73,5 | 294.1 kb | 67 | 6097 | NZ_VDLY00000000.2 | GCF_006334995.2 |
| *Streptomyces triticirhizae* NEAU-YY642^T^ | 6,6 | 73,5 | 25.8 kb | 68 | 5944 | NZ_RFFJ00000000.1 | GCF_003696235.1 |
| DSM 41981 | 9,2 | 73 | 254.1 kb | 105 | 8068 | JAVRES000000000 | GCF_031845505.1 |
| DSM 41014 | 9,1 | 72 | 156.2 kb | 92 | 8193 | JAVRFF000000000 | GCF_031845745.1 |
| DSM 41640 | 11,3 | 70 | 430.6 kb | 93 | 10622 | JAVREZ000000000 | GCF_031845645.1 |
| *Streptomyces canus* DSM 40275 | 9,8 | 70,5 | 219 kb | 78 | 8903 | NZ_LMWO00000000.1 | GCF_001514045.1 |
| *Streptomyces canus* DSM 40017T | 11,6 | 70 | 309.6 kb | 80 | 10545 | NZ_LMWU00000000.1 | GCF_001514145.1 |
| DSM 41972 | 6,6 | 72 | 141 kb | 93 | 6111 | JAVSGH000000000 | GCA_032027505.1 |
| DSM 41636 | 7,9 | 71,5 | 103.2 kb | 91 | 7280 | JAVRFA000000000 | GCF_031845685.1 |
| DSM 41635 | 7,8 | 71,5 | 225.1 kb | 94 | 7304 | JAVRFB000000000 | GCF_031845705.1 |
| *Streptomyces calvus* JCM 4326^T^ | 7,9 | 72,5 | 295 kb | 84 | 6810 | NZ_BMSU00000000.1 | GCF_014649315.1 |
| *Streptomyces calvus* DSM 41692 | 8 | 72,5 | 2.7 Mb | 97 | 6759 | NZ_JACJHU000000000.1 | GCF_014138545.1 |
| *Streptomyces calvus* DSM 41452 | 7,8 | 72,5 | 7.8 Mb | 83 | 6554 | NZ_CP022310.1 | GCF_006716135.1 |
| *Streptomyces capillispiralis* JCM 5075^T^ | 8,4 | 72,5 | 187.2 kb | 78 | 7159 | NZ_BNCE00000000.1 | GCF_014656315.1 |
| *Streptomyces albaduncus* JCM 4715^T^ | 7,9 | 71,5 | 70.1 kb | 79 | 7117 | NZ_BMVH00000000.1 | GCF_014650615.1 |
| DSM 41921 | 8,3 | 72,5 | 316.1 kb | 93 | 7396 | JAVREU000000000 | GCF_031845545.1 |
| *Streptomyces djakartensis* JCM 4957^T^ | 7,7 | 71 | 341.7 kb | 83 | 6879 | NZ_BMWE00000000.1 | GCF_014651075.1 |
| DSM 40712 | 9,3 | 71,5 | 137.4 kb | 89 | 8506 | JAVRFH000000000 | GCF_031845825.1 |
| *Streptomyces ambofaciens* ATCC 23877^T^ | 8,4 | 72 | 8.3 Mb | 92 | 7423 | NZ_CP012382.1 | GCF_001267885.1 |
| DSM 3412 | 10,1 | 71 | 183.3 kb | 91 | 8936 | JAVRFJ000000000 | GCF_031845845.1 |
| *Streptomyces akebiae* M G28^T^ | 10,5 | 71 | 10.3 Mb | 91 | 9033 | NZ_CP080647.1 | GCF_019599145.1 |
| DSM 41699 | 10,3 | 70,5 | 97.4 kb | 88 | 9483 | JAVREY000000000 | GCF_031845665.1 |
| *Streptomyces glomeratus* DSM 41457^T^ | 7,8 | 71 | 202.4 kb | 77 | 7047 | NZ_JAKEEB000000000.1 | GCF_021462825.1 |
| DSM 41770 | 7,9 | 71,5 | 427.3 kb | 83 | 7181 | JAVREX000000000 | GCF_031845605.1 |
| *Streptomyces griseolus* NRRL B-2925^T^ | 7,5 | 72 | 201.6 kb | 73 | 6551 | NZ_JOFC00000000.1 | GCF_000721185.1 |
| *Streptomyces nitrosporeus* JCM 4598^T^ | 7,9 | 72 | 272.7 kb | 86 | 6958 | NZ_BMUV00000000.1 | GCF_014650375.1 |
| DSM 40932 | 7,9 | 72 | 131.6 kb | 87 | 6978 | JAVRFG000000000 | GCF_031845765.1 |
| *Streptomyces microflavus* JCM 4496^T^ | 8,5 | 71 | 540.4 kb | 85 | 7515 | NZ_BMUG00000000.1 | GCF_014650075.1 |
| *Streptomyces cyaneofuscatus* NRRL B-2570^T^ | 7,9 | 71,5 | 353.8 kb | 77 | 7101 | NZ_JOEM00000000.1 | GCF_000718135.1 |
| *Streptomyces arboris* TRM68085^T^ | 8,3 | 71,5 | 97.2 kb | 73 | 7494 | NZ_VYUA00000000.1 | GCF_008932075.1 |
| DSM 41979 | 7,5 | 73,5 | 67.1 kb | 101 | 6568 | JAVRET000000000 | GCF_031845585.1 |
| DSM 41859 | 7,3 | 73,5 | 101.9 kb | 94 | 6296 | JAVREW000000000 | GCF_031845625.1 |
| DSM 41982 | 7,1 | 73,5 | 110.3 kb | 82 | 6073 | JAVRER000000000 | GCF_031845525.1 |
| DSM 40473 | 7,7 | 71,5 | 250 kb | 100 | 6779 | JAVRFI000000000 | GCF_031845785.1 |
| *Streptomyces abikoensis* JCM 4002^T^ | 7 | 72 | 785.1 kb | 91 | 6152 | NZ_BMRT00000000.1 | GCF_014648775.1 |
| DSM 41529 | 11,9 | 71 | 299.4 kb | 97 | 10824 | JAVRFD000000000 | GCF_031845865.1 |
| *Streptomyces sparsogenes* ATCC 25498^T^ | 8,9 | 72,5 | 72.4 kb | 88 | 7889 | NZ_MAXF00000000.1 | GCF_001704635.1 |
| DSM 42041 | 6,5 | 73 | 181.3 kb | 81 | 5860 | JAVREQ000000000 | GCF_031845485.1 |
| *Streptomyces gossypii* N2-109^T^ | 7,5 | 70,5 | 343.2 kb | 73 | 6727 | NZ_JAJAGO000000000.1 | GCF_025165795.1 |
| DSM 41527 | 8,4 | 71 | 125.7 kb | 91 | 7480 | JAVRFE000000000 | GCF_031845725.1 |
| *Streptomyces caniferus* NBRC 15389^T^ | 9,1 | 71,5 | 4.9 Mb | 91 | 7962 | NZ_BLIN00000000.1 | GCF_009811555.1 |
| *Streptomyces angustmyceticus* JCM 4053^T^ | 8,1 | 72 | 8.1 Mb | 91 | 7005 | NZ_CP082945.1 | GCF_019933235.1 |
| DSM 41528 | 9,1 | 72 | 173.6 kb | 86 | 8150 | JAZBJP000000000 | GCF_036352915.1 |
| *Streptomyces endophytica* HNM0140^T^ | 6,9 | 72 | 6.9 Mb | 87 | 6046 | NZ_CP110636.1 | GCF_026153355.1 |
| DSM 41602 | 10,6 | 71 | 261 kb | 87 | 9193 | JAZBJQ000000000 | GCF_036352895.1 |
| *Streptomyces antimycoticus* NBRC 12839^T^ | 11,2 | 71 | 10.9 Mb | 85 | 9775 | NZ_BJHV00000000.1 | GCF_005405925.1 |
| DSM 41524 | 11,4 | 71 | 222.3 kb | 106 | 9705 | JAZBJO000000000 | GCF_036352935.1 |
| *Streptomyces asiaticus* DSM 41761^T^ | 11,9 | 71 | 10.1 kb | 90 | 10007 | NZ_JAGSHX000000000.1 | GCF_018138715.1 |
| *Blastococcus* sp*.* DSM 46792 | 4,4 | 73,5 | 213.3 kb | 57 | 4258 | JAVREI000000000 | GCF_031845325.1 |
| *Blastococcus aurantiacus* DSM 44268^T^ | 4,2 | 73,5 | 441.3 kb | 59 | 4047 | NZ_FNBT00000000.1 | GCF_900102425.1 |
| *Pseudonocardia* sp. DSM 45834 | 6,7 | 72 | 210.6 kb | 57 | 6391 | JAVREJ000000000 | GCF_031845345.1 |
| *Pseudonocardia humida* S2-4^T^ | 8,3 | 74,5 | 110.3 kb | 54 | 7956 | NZ_JAGSOV000000000.1 | GCF_023898865.1 |
| *Pseudonocardia kunmingensis* DSM 45301^T^ | 9,1 | 73,5 | 1.6 Mb | 59 | 8624 | NZ_VFPA00000000.1 | GCF_006716445.1 |
| *Pseudonocardia cypriaca* DSM 45511^T^ | 8,3 | 72,5 | 3 Mb | 56 | 7840 | NZ_VFPH00000000.1 | GCF_006717045.1 |
| *Streptomonospora* sp. DSM 45055 | 5,6 | 72,5 | 179.7 kb | 70 | 4890 | JAVREK000000000 | GCF_031845375.1 |
| *Streptomonospora alba* YIM 90003^T^ | 5,9 | 72 | 126.2 kb | 61 | 5148 | NZ_JROO00000000.1 | GCF_000826685.1 |
| *Streptomonospora salina* DSM 44593^T^ | 5,6 | 72 | 5.1 Mb | 75 | 5031 | NZ_JACHLY000000000.1 | GCF_014204715.1 |
| *Nocardiopsis* sp. DSM 44743 | 6,2 | 71,5 | 272.5 kb | 85 | 5426 | JAVREP000000000 | GCF_031845465.1 |
| *Nocardiopsis changdeensis* Mg02^T^ | 7,4 | 73,5 | 7.2 Mb | 83 | 6588 | NZ_CP074133.1 | GCF_018316655.1 |
| *Nocardiopsis flavescens* CGMCC 4.5723^T^ | 7,2 | 74 | 263.2 kb | 71 | 6170 | NZ_FQZK00000000.1 | GCF_900141985.1 |
| *Jatrophihabitans* sp. DSM 44399 | 5,2 | 68 | 96 kb | 53 | 5016 | JAVREH000000000 | GCF_031845305.1 |
| *Jatrophihabitans telluris* N237^T^ | 4,2 | 68 | 4.2 Mb | 53 | 3811 | NZ_CP097332.1 | GCF_023516435.1 |

**Table S2.** 16S rRNA similarity between the studied strains and their closest relatives

| **Strains** | **16S rRNA similarity %** | **closest relative** | **Accession Number of the closest neighbour** | **Length (bp) of the studied strains** |
| --- | --- | --- | --- | --- |
| 3421 | 99.84 | *Streptomyces scabiei* | LBNJ01000196 | 1318 |
|  |  | *Streptomyces europaeiscabiei* | AY207598 |  |
| 40712 | 99.44 | *Streptomyces levis* | AB184670 | 1517 |
| 41014 | 99.38 | *Streptomyces venetus* | LC073310 | 1513 |
| 41529 | 99.50 | *Streptomyces aldersoniae* | EU170123 | 1436 |
| 41640 | 100 | *Streptomyces canus* | KQ948708 | 1529 |
| 41699 | 99.38 | *Streptomyces glomeratus* | AJ781754 | 1513 |
| 41770 | 99.93 | *Streptomyces flavovirens* | AB184834 | 1514 |
| 41921 | 99.45 | *Streptomyces purpurascens* | AB184859 | 1513 |
|  |  | *Streptomyces spinoverrucosus* | AB184578 |  |
| 41981 | 99.52 | *Streptomyces venetus* | LC073310 | 1513 |
| 41982 | 99.52 | *Streptomyces xylanilyticus* | LC128341 | 1517 |
| 44915 | 98.23 | *Streptomyces triticirhizae* | MH144589 | 1531 |
|  | 98.49 | *Streptomyces triticirhizae* | MH144589 | 1413 |
| 44917 | 99.59 | *Streptomyces klenkii* | KF656772 | 1517 |
| 44918 | 99.42 | *Streptomyces mayteni* | EU200683 | 1516 |
| 44938 | 99.29 | *Streptomyces manganisoli* | KY911452 | 1522 |
| 41886 | 98.59 | *Streptomyces marincola* | MZ889118 | 1545 |
| 41859 | 99.44 | *Streptomyces xylanilyticus* | LC128341 | 1508 |
| 40473 | 99.57 | *Streptomyces hiroshimensis* | AB184802 | 1450 |
| 42041 | 100 | *Streptomyces qinglanensis* | HQ660227 | 814 |
| 41524 | 99.86 | *Streptomyces rhizosphaericus* | AB249941 | 1533 |
| 41527 | 99.86 | *Streptomyces angustmyceticus* | MUAY01000275 | 1520 |
|  |  | *Streptomyces nigrescens* | AB184225 |  |
|  |  | *Streptomyces libani subsp. libani* | AB184414 |  |
| 41528 | 99.86 | *Streptomyces lydicus* | RDTD01000009 | 1522 |
|  |  | *Streptomyces chattanoogensis* | LGKG01000206 |  |
| 41602 | 99.93 | *Streptomyces antimycoticus* | AB184185 | 1533 |
| 41972 | 99.86 | *Streptomyces althioticus* | AY999791 | 1512 |
| 41635 | 99.58 | *Streptomyces rubrogriseus* | AJ781373 | 1515 |
| 41636 | 99.58 | *Streptomyces rubrogriseus* | AJ781373 | 1519 |
| 41979 | 99.44 | *Streptomyces xylanilyticus* | LC128341 | 1511 |
| 40932 | 99.63 | *Streptomyces cavourensis* | AB184264 | 1112 |
| 46792 | 99.72 | *Blastococcus fimeti* | MH479059 | 1507 |
| 45834 | 97.78 | *‘Pseudonocardia humida’* | MW442970 |  |
| 45055 | 99.48 | *Streptomonospora arabica* | EF095149 | 1443 |
| 44743 | 99.33 | *Nocardiopsis flavescens* | jgi.1085022 | 955 |
| 44399 | 94.84 | *Jatrophihabitans telluris* | LC323105 | 1467 |

**Table S3**. dDDH between the strains and their close phylogenomic neighbours

| **Query strain** | **Subject strain** | **dDDH (d4. in %)** | **G+C content difference (in %)** |
| --- | --- | --- | --- |
| *Streptomyces* sp. DSM 41859 | *Streptomyces* sp. DSM 41979^T^ | 87.1 | 0.00 |
| *Streptomyces* sp. DSM 41635 | *Streptomyces* sp. DSM 41636^T^ | 82.1 | 0.36 |
| *Streptomyces* sp. DSM 41859 | *Streptomyces* sp. DSM 41982 | 81.6 | 0.11 |
| *Streptomyces* sp. DSM 41982 | *Streptomyces* sp. DSM 41979^T^ | 80.7 | 0.11 |
| *Streptomyces* sp. DSM 41972^T^ | *Streptomyces althioticus* JCM 4344^T^ | 72.3 | 0.30 |
| *Streptomyces* sp. DSM 41972^T^ | *Streptomyces matensis* JCM 4277^T^ | 72.0 | 0.42 |
| *Streptomyces* sp. DSM 41972^T^ | *Streptomyces griseorubens* JCM 4383^T^ | 68.7 | 0.18 |
| *Streptomyces* sp. DSM 41972^T^ | *Streptomyces griseorubens* DSM 40160^T^ | 68.7 | 0.29 |
| *Streptomyces* sp. DSM 3412^T^ | *Streptomyces akebiae* MG28^T^ | 64.7 | 0.11 |
| *Streptomyces* sp. DSM 41640^T^ | *Streptomyces canus* DSM 40017^T^ | 64.6 | 0.25 |
| *Streptomyces* sp. DSM 41640^T^ | *Streptomyces ciscaucasicus* DSM 40275^T^ | 64.1 | 0.35 |
| *Streptomyces* sp. DSM 40473^T^ | *Streptomyces abikoensis* JCM 4002^T^ | 47.1 | 0.57 |
| *Streptomyces* sp. DSM 3412^T^ | *Streptomyces deccanensis* KCTC 19241^T^ | 46.8 | 0.14 |
| *Streptomyces* sp. DSM 3412^T^ | *Streptomyces caniscabiei* NE06-02D^T^ | 45.9 | 0.29 |
| *Streptomyces* sp. DSM 41527^T^ | *Streptomyces caniferus* NBRC 15389^T^ | 42.5 | 0.23 |
| *Streptomyces* sp. DSM 40932^T^ | *Streptomyces arboris* TRM 68085^T^ | 42.2 | 0.56 |
| *Streptomyces* sp. DSM 41014^T^ | *Streptomyces* sp. DSM 41981 | 41.9 | 0.89 |
| *Streptomyces* sp. DSM 41527^T^ | *Streptomyces angustmyceticus* JCM 4053^T^ | 41.7 | 1.12 |
| *Streptomyces* sp. DSM 40932^T^ | *Streptomyces cyaneofuscatus* NRRL B-2570^T^ | 41.7 | 0.42 |
| *Streptomyces* sp. DSM 40932^T^ | *Streptomyces microflavus* NBRC 13062^T^ | 41.6 | 0.80 |
| *Streptomyces* sp. DSM 40932^T^ | *Streptomyces microflavus* JCM 4496^T^ | 41.6 | 0.74 |
| *Streptomyces* sp. DSM 41699^T^ | *Streptomyces glomeratus* DSM 41457^T^ | 38.1 | 0.35 |
| *Streptomyces* sp. DSM 41770^T^ | *Streptomyces griseolus* NRRL B-2925^T^ | 38.0 | 0.29 |
| *Streptomyces* sp. DSM 41770^T^ | *Streptomyces halstedii* NRRL-ISP 5068^T^ | 37.8 | 0.22 |
| *Streptomyces* sp. DSM 40712^T^ | *Streptomyces ambofaciens* ATCC 23877^T^ | 37.1 | 0.76 |
| *Streptomyces* sp. DSM 41529^T^ | *Streptomyces sparsogenes* DSM 40356^T^ | 37.0 | 0.99 |
| *Streptomyces* sp. DSM 41529^T^ | *Streptomyces sparsogenes* ATCC 25498^T^ | 36.8 | 1.14 |
| *Streptomyces* sp. DSM 41921^T^ | *Streptomyces djakartensis* JCM 4957^T^ | 36.2 | 1.14 |
| *Streptomyces* sp. DSM 41636^T^ | *Streptomyces griseoloalbus* JCM 4480^T^ | 35.5 | 0.57 |
| *Streptomyces* sp. DSM 41635 | *Streptomyces albaduncus* CECT 3226^T^ | 35.4 | 1.00 |
| *Streptomyces* sp. DSM 41635 | *Streptomyces griseoloalbus* JCM 4480^T^ | 35.4 | 0.93 |
| *Streptomyces* sp. DSM 41636^T^ | *Streptomyces albaduncus* CECT 3226^T^ | 35.4 | 0.64 |
| *Streptomyces* sp. DSM 41636^T^ | *Streptomyces albaduncus* JCM 4715^T^ | 35.3 | 0.56 |
| *Streptomyces* sp. DSM 41635 | *Streptomyces albaduncus* JCM 4715^T^ | 35.3 | 0.93 |
| *Streptomyces* sp. DSM 41636^T^ | *Streptomyces capillispiralis* JCM 5075^T^ | 35.2 | 1.42 |
| *Streptomyces* sp. DSM 41635 | *Streptomyces capillispiralis* JCM 5075^T^ | 35.1 | 1.78 |
| *Streptomyces* sp. DSM 41636^T^ | *Streptomyces aureorectus* DSM 41692^T^ | 34.3 | 1.23 |
| *Streptomyces* sp. DSM 41636^T^ | *Streptomyces calvus* JCM 4326^T^ | 34.2 | 1.29 |
| *Streptomyces* sp. DSM 41635 | *Streptomyces asterosporus* DSM 41452^T^ | 34.2 | 1.71 |
| *Streptomyces* sp. DSM 41636^T^ | *Streptomyces asterosporus* DSM 41452^T^ | 34.2 | 1.34 |
| *Streptomyces* sp. DSM 41635 | *Streptomyces aureorectus* DSM 41692 | 34.1 | 1.59 |
| *Streptomyces* sp. DSM 41635 | *Streptomyces calvus* JCM 4326^T^ | 34.0 | 1.66 |
| *Streptomyces* sp. DSM 41636^T^ | *Streptomyces griseoflavus* JCM 4479^T^ | 33.8 | 1.10 |
| *Streptomyces* sp. DSM 41635 | *Streptomyces griseoflavus* JCM 4479^T^ | 33.6 | 1.46 |
| *Streptomyces* sp. DSM 44918^T^ | *Streptomyces hainanensis* DSM 41900^T^ | 33.0 | 0.04 |
| *Streptomyces* sp. DSM 41770^T^ | *Streptomyces nitrosporeus* ATCC 12769^T^ | 31.6 | 0.48 |
| *Streptomyces* sp. DSM 41770^T^ | *Streptomyces nitrosporeus* JCM 4598^T^ | 31.5 | 0.44 |
| *Streptomyces* sp. DSM 41699^T^ | *Streptomyces colonosanans* MUSC 93^T^ | 30.9 | 0.66 |
| *Streptomyces* sp. DSM 41527^T^ | *Streptomyces endophytica* HNM0140^T^ | 30.9 | 1.09 |
| *Streptomyces* sp. DSM 41635 | *Streptomyces* sp. DSM 41972^T^ | 30.5 | 1.40 |
| *Streptomyces* sp. DSM 41636^T^ | *Streptomyces* sp. DSM 41972^T^ | 30.4 | 1.03 |
| *Streptomyces* sp. DSM 41699^T^ | *Streptomyces macrolidinus* RY43-2^T^ | 29.0 | 0.22 |
| *Streptomyces* sp. DSM 44915^T^ | *Streptomyces triticirhizae* NEAU-YY642^T^ | 28.4 | 0.40 |
| *Streptomyces* sp. DSM 40712^T^ | *Streptomyces* sp. DSM 41921^T^ | 27.9 | 0.97 |
| *Streptomyces* sp. DSM 44915^T^ | *Streptomyces mimosae* 3MP-10^T^ | 27.9 | 0.30 |
| *Streptomyces* sp. DSM 41636^T^ | *Streptomyces* sp. DSM 41921^T^ | 27.8 | 1.22 |
| *Streptomyces* sp. DSM 41635 | *Streptomyces* sp. DSM 41921^T^ | 27.7 | 1.59 |
| *Streptomyces* sp. DSM 41886^T^ | *Streptomyces marincola* SCSIO 64649^T^ | 27.7 | 1.10 |
| *Streptomyces* sp. DSM 40712^T^ | *Streptomyces* sp. DSM 41636^T^ | 27.7 | 0.25 |
| *Streptomyces* sp. DSM 40712^T^ | *Streptomyces* sp. DSM 41635 | 27.5 | 0.62 |
| *Streptomyces* sp. DSM 41921^T^ | *Streptomyces* sp. DSM 41972^T^ | 27.3 | 0.19 |
| *Streptomyces* sp. DSM 44915 | *Streptomyces zhaozhouensis* CGMCC 4.7095^T^ | 27.2 | 0.33 |
| *Streptomyces* sp. DSM 40712^T^ | *Streptomyces* sp. DSM 41972^T^ | 27.0 | 0.78 |
| *Streptomyces* sp. DSM 44915^T^ | *Streptomyces sedi* JCM 16909^T^ | 26.9 | 0.79 |
| *Streptomyces* sp. DSM 41640^T^ | *Streptomyces* sp. DSM 41921^T^ | 26.6 | 2.40 |
| *Streptomyces* sp. DSM 40932^T^ | *Streptomyces* sp. DSM 41770^T^ | 26.6 | 0.28 |
| *Streptomyces* sp. DSM 40712^T^ | *Streptomyces* sp. DSM 41640^T^ | 26.4 | 1.43 |
| *Streptomyces* sp. DSM 41014^T^ | *Streptomyces* sp. DSM 41640^T^ | 26.1 | 2.03 |
| *Streptomyces* sp. DSM 41014^T^ | *Streptomyces canus* DSM 40017^T^ | 26.1 | 1.78 |
| *Streptomyces* sp. DSM 41014^T^ | *Streptomyces ciscaucasicus* DSM 40275^T^ | 26.1 | 1.68 |
| *Streptomyces* sp. DSM 41014^T^ | *Streptomyces* sp. DSM 41921^T^ | 26.0 | 0.36 |
| *Streptomyces* sp. DSM 41921^T^ | *Streptomyces* sp. DSM 41981 | 26.0 | 0.53 |
| *Streptomyces* sp. DSM 41886^T^ | *Streptomyces* sp. DSM 44938^T^ | 25.9 | 0.12 |
| *Streptomyces* sp. DSM 40712^T^ | *Streptomyces* sp. DSM 41014^T^ | 25.9 | 0.61 |
| *Streptomyces* sp. DSM 40712^T^ | *Streptomyces* sp. DSM 41981 | 25.9 | 1.50 |
| *Streptomyces* sp. DSM 3412^T^ | *Streptomyces* sp. DSM 41640^T^ | 25.8 | 0.99 |
| *Streptomyces* sp. DSM 41636^T^ | *Streptomyces* sp. DSM 41640^T^ | 25.7 | 1.18 |
| *Streptomyces* sp. DSM 41981 | *Streptomyces ciscaucasicus* DSM 40275^T^ | 25.7 | 2.58 |
| *Streptomyces* sp. DSM 41640^T^ | *Streptomyces* sp. DSM 41981 | 25.7 | 2.93 |
| *Streptomyces* sp. DSM 41981 | *Streptomyces canus* DSM 40017^T^ | 25.7 | 2.68 |
| *Streptomyces* sp. DSM 41635 | *Streptomyces* sp. DSM 41640^T^ | 25.6 | 0.81 |
| *Streptomyces* sp. DSM 41014^T^ | *Streptomyces* sp. DSM 41636^T^ | 25.6 | 0.86 |
| *Streptomyces* sp. DSM 41014^T^ | *Streptomyces* sp. DSM 41635 | 25.6 | 1.22 |
| *Streptomyces* sp. DSM 40712^T^ | *Streptomyces* sp. DSM 41699^T^ | 25.6 | 0.85 |
| *Streptomyces* sp. DSM 41699^T^ | *Streptomyces* sp. DSM 41921^T^ | 25.6 | 1.82 |
| *Streptomyces* sp. DSM 41014^T^ | *Streptomyces* sp. DSM 41972^T^ | 25.5 | 0.17 |
| *Streptomyces* sp. DSM 3412^T^ | *Streptomyces* sp. DSM 40712^T^ | 25.4 | 0.44 |
| *Streptomyces* sp. DSM 41636^T^ | *Streptomyces* sp. DSM 41981 | 25.4 | 1.75 |
| *Streptomyces* sp. DSM 41635 | *Streptomyces* sp. DSM 41981 | 25.4 | 2.12 |
| *Streptomyces* sp. DSM 41972^T^ | *Streptomyces* sp. DSM 41981 | 25.3 | 0.72 |
| *Streptomyces* sp. DSM 3412^T^ | *Streptomyces* sp. DSM 41921^T^ | 25.3 | 1.41 |
| *Streptomyces* sp. DSM 41640^T^ | *Streptomyces* sp. DSM 41699^T^ | 25.3 | 0.57 |
| *Streptomyces* sp. DSM 41636^T^ | *Streptomyces* sp. DSM 41699^T^ | 25.2 | 0.60 |
| *Streptomyces* sp. DSM 41014^T^ | *Streptomyces* sp. DSM 41699^T^ | 25.2 | 1.46 |
| *Streptomyces* sp. DSM 3412^T^ | *Streptomyces* sp. DSM 41699^T^ | 25.1 | 0.41 |
| *Streptomyces* sp. DSM 41640^T^ | *Streptomyces* sp. DSM 41972^T^ | 25.1 | 2.21 |
| *Streptomyces* sp. DSM 41635 | *Streptomyces* sp. DSM 41699^T^ | 25.1 | 0.24 |
| *Streptomyces* sp. DSM 41699^T^ | *Streptomyces* sp. DSM 41972^T^ | 25.1 | 1.64 |
| *Streptomyces* sp. DSM 41699^T^ | *Streptomyces* sp. DSM 41981 | 25.0 | 2.36 |
| *Streptomyces* sp. DSM 3412^T^ | *Streptomyces* sp. DSM 41636^T^ | 24.9 | 0.19 |
| *Streptomyces* sp. DSM 3412^T^ | *Streptomyces* sp. DSM 41635 | 24.9 | 0.18 |
| *Streptomyces* sp. DSM 3412^T^ | *Streptomyces* sp. DSM 41014^T^ | 24.6 | 1.05 |
| *Streptomyces* sp. DSM 3412^T^ | *Streptomyces* sp. DSM 41981 | 24.6 | 1.94 |
| *Streptomyces* sp. DSM 3412^T^ | *Streptomyces* sp. DSM 41972^T^ | 24.6 | 1.22 |
| *Streptomyces* sp. DSM 40932^T^ | *Streptomyces* sp. DSM 41529^T^ | 24.5 | 0.76 |
| *Streptomyces* sp. DSM 44917^T^ | *Streptomyces specialis* GW41-1564^T^ | 23.8 | 2.01 |
| *Streptomyces* sp. DSM 44917^T^ | *Streptomyces* sp. DSM 44938^T^ | 23.8 | 2.37 |
| *Streptomyces* sp. DSM 44938^T^ | *Streptomyces radicis* DS1-2^T^ | 23.7 | 0.85 |
| *Streptomyces* sp. DSM 44918^T^ | *Streptomyces hoynatensis* KCTC 29097^T^ | 23.5 | 1.43 |
| *Streptomyces* sp. DSM 41979^T^ | *Streptomyces speibonae* NRRL B-24240^T^ | 23.4 | 1.42 |
| *Streptomyces* sp. DSM 41859 | *Streptomyces speibonae* NRRL B-24240^T^ | 23.4 | 1.42 |
| *Streptomyces* sp. DSM 41982 | *Streptomyces speibonae* NRRL B-24240^T^ | 23.4 | 1.53 |
| *Streptomyces* sp. DSM 44918^T^ | *Streptomyces* sp. DSM 44938^T^ | 23.4 | 0.64 |
| *Streptomyces* sp. DSM 40932^T^ | *Streptomyces* sp. DSM 41635 | 23.3 | 1.17 |
| *Streptomyces* sp. DSM 40932^T^ | *Streptomyces* sp. DSM 41014^T^ | 23.3 | 0.06 |
| *Streptomyces* sp. DSM 40932^T^ | *Streptomyces* sp. DSM 41640^T^ | 23.2 | 1.98 |
| *Streptomyces* sp. DSM 40932^T^ | *Streptomyces* sp. DSM 41636^T^ | 23.1 | 0.80 |
| *Streptomyces* sp. DSM 40712^T^ | *Streptomyces* sp. DSM 41770^T^ | 23.0 | 0.27 |
| *Streptomyces* sp. DSM 41527^T^ | *Streptomyces* sp. DSM 40473^T^ | 23.0 | 0.57 |
| *Streptomyces* sp. DSM 41886^T^ | *Streptomyces* sp. DSM 44917^T^ | 23.0 | 2.25 |
| *Streptomyces* sp. DSM 40932^T^ | *Streptomyces* sp. DSM 40712^T^ | 23.0 | 0.55 |
| *Streptomyces* sp. DSM 40932^T^ | *Streptomyces* sp. DSM 41527^T^ | 23.0 | 0.86 |
| *Streptomyces* sp. DSM 40932^T^ | *Streptomyces* sp. DSM 41981 | 23.0 | 0.95 |
| *Streptomyces* sp. DSM 41527^T^ | *Streptomyces* sp. DSM 41529^T^ | 22.9 | 0.10 |
| *Streptomyces* sp. DSM 40932^T^ | *Streptomyces* sp. DSM 41699^T^ | 22.8 | 1.40 |
| *Streptomyces* sp. DSM 41602 | *Streptomyces antimycoticus* NBRC 12839^T^ | 75.6 | 0.03 |
| *Streptomyces* sp. DSM 41524^T^ | *Streptomyces asiaticus* DSM 41761^T^ | 71.6 | 0.16 |
| *Streptomyces* sp. DSM 41524^T^ | *Streptomyces indonesiensis* DSM 41759^T^ | 71.5 | 0.09 |
| *Streptomyces* sp. DSM 41524^T^ | *Streptomyces cangkringensis* DSM 41769^T^ | 71.5 | 0.13 |
| *Streptomyces* sp. DSM 41524^T^ | *Streptomyces rhizosphaericus* DSM 41760^T^ | 71.0 | 0.09 |
| *Streptomyces* sp. DSM 41528^T^ | *Streptomyces endophytica* HNM0140^T^ | 70.8 | 0.47 |
| *Streptomyces* sp. DSM 41528^T^ | *Streptomyces inhibens* NEAU-D10^T^ | 32.1 | 1.27 |
| *Streptomyces* sp. DSM 41528^T^ | *Streptomyces decoyicus* NRRL ISP-5087 | 31.9 | 1.00 |
| *Blastococcus* sp. DSM 46792^T^ | *Blastococcus aurantiacus* DSM 44268^T^ | 31.4 | 0.31 |
| *Nocardiopsis* sp. DSM 44743^T^ | *Nocardiopsis changdeensis* Mg02^T^ | 30.4 | 2.19 |
| *Nocardiopsis* sp. DSM 44743^T^ | *Nocardiopsis flavescens* CGMCC 4.5723^T^ | 29.1 | 2.81 |
| *Streptomonospora* sp. DSM 45055^T^ | *Streptomonospora alba* YIM 90003^T^ | 28.1 | 0.69 |
| *Streptomonospora* sp. DSM 45055^T^ | *Streptomonospora salina* DSM 44593^T^ | 26.8 | 0.50 |
| *Nocardiopsis* sp. DSM 44743^T^ | *Nocardiopsis algeriensis* CECT 8712^T^ | 24.2 | 0.05 |
| *Pseudonocardia* sp. DSM 45834^T^ | *Pseudonocardia kunmingensis* DSM 45301^T^ | 22.0 | 1.50 |
| *Pseudonocardia* sp. DSM 45834^T^ | *Pseudonocardia humida* S2-4^T^ | 21.9 | 2.63 |
| *Pseudonocardia* sp. DSM 45834^T^ | *Pseudonocardia xinjiangensis* JCM 11839^T^ | 21.8 | 0.09 |
| *Pseudonocardia* sp. DSM 45834^T^ | *Pseudonocardia cypriaca* DSM 45511^T^ | 21.7 | 0.81 |
| *Pseudonocardia* sp. DSM 45834^T^ | *Pseudonocardia alaniniphila* Y-16303^T^ | 21.3 | 1.48 |
| *Pseudonocardia* sp. DSM 45834^T^ | *Pseudonocardia petroleophila* CGMCC 4.1532^T^ | 21.3 | 2.11 |
| *Pseudonocardia* sp. DSM 45834^T^ | *Pseudonocardia oceani* KRD185^T^ | 21.2 | 2.05 |
| *Pseudonocardia* sp. DSM 45834^T^ | *Pseudonocardia abyssalis* KRD168^T^ | 21.1 | 1.50 |
| *Jatrophihabitans* sp. DSM 44399^T^ | *Jatrophihabitans telluris* KCTC 39922^T^ | 20.4 | 0.10 |
| *Jatrophihabitans* sp. DSM 44399^T^ | *Jatrophihabitans endophyticus* DSM 45627^T^ | 19.9 | 4.71 |

**Table S4.** Growth properties of the studied strains (in an Excel file)

**Table S5.** Biochemical and enzymatic profile of the studied strains (in an Excel file).

**Table S6.** Chemotaxonomic features of the studied strains and their closest neighbours (in an Excel file).

**Table S7.** Statistics on GCFs of the proposed novel type strains.

| **cutoff** | **all BGCs** | | | | **BGCs inside contigs** | | | | **BGCs on contig edge** | | | | **BGCs from new strains on contig edge that clustered with BGCs inside contigs** |
| --- | --- | --- | --- | --- | --- | --- | --- | --- | --- | --- | --- | --- | --- |
|  | **BGCs from new strains** | **GCFs with new strains** | **singleton GCFs from new strains** | **known GCFs with new strains** | **BGCs from new strains** | **GCFs with new strains** | **singleton GCFs from new strains** | **known GCFs with new strains** | **BGCs from new strains** | **GCFs with new strains** | **singleton GCFs from new strains** | **known GCFs with new strains** |  |
| 0,3 | 982 | 745 | 481 | 57 | 575 | 449 | 265 | 40 | 407 | 352 | 275 | 37 | 126 |

| **Strains** | **Genome size** | **G+C content mol %** | **N50** | **RNA numbers** | **coding sequences** | **NCBI accession number** | **BGCs separated** | **# BGCs** | **# on contig edge** |
| --- | --- | --- | --- | --- | --- | --- | --- | --- | --- |
| **DSM 41886** | 7.53 | 72.5 | 373560 | 57 | 6849 | JAVREV000000000 | yes | 42 | 19 |
| **DSM 44938** | 7.65 | 72.5 | 288245 | 56 | 7365 | JAVREL000000000 | yes | 30 | 13 |
| **DSM 44917** | 6.05 | 74.7 | 108347 | 58 | 5674 | JAVREN000000000 | yes | 23 | 10 |
| **DSM 44918** | 8.03 | 73.0 | 98825 | 63 | 7655 | JAVREM000000000 | yes | 30 | 13 |
| **DSM 44915** | 7.51 | 73.7 | 309249 | 65 | 6888 | JAVREO000000000 | yes | 25 | 10 |
| **DSM 41981** | 9.23 | 72.9 | 254073 | 79 | 8512 | JAVRES000000000 | yes | 38 | 14 |
| **DSM 41014** | 9.09 | 72.0 | 156173 | 80 | 8496 | JAVRFF000000000 | yes | 42 | 20 |
| **DSM 41640** | 11.26 | 70.0 | 430585 | 83 | 11127 | JAVREZ000000000 | yes | 29 | 6 |
| **DSM 41972** | 6.64 | 72.2 | 140976 | 75 | 6523 | JAVSGH000000000 | yes | 19 | 9 |
| **DSM 41636** | 7.96 | 71.2 | 103164 | 79 | 7519 | JAVRFA000000000 | yes | 22 | 11 |
| **DSM 41635** | 7.93 | 70.8 | 225144 | 80 | 7566 | JAVRFB000000000 | yes | 17 | 6 |
| **DSM 41921** | 8.26 | 72.4 | 316072 | 77 | 7678 | JAVREU000000000 | yes | 31 | 5 |
| **DSM 40712** | 9.33 | 71.4 | 137395 | 76 | 9091 | JAVRFH000000000 | yes | 52 | 30 |
| **DSM 3412** | 10.10 | 71.0 | 183349 | 79 | 9451 | JAVRFJ000000000 | yes | 42 | 17 |
| **DSM 41699** | 10.28 | 70.6 | 97361 | 76 | 10235 | JAVREY000000000 | yes | 32 | 15 |
| **DSM 41770** | 7.92 | 71.7 | 427311 | 73 | 7526 | JAVREX000000000 | yes | 29 | 9 |
| **DSM 40932** | 7.94 | 72.0 | 131579 | 74 | 7296 | JAVRFG000000000 | yes | 46 | 29 |
| **DSM 41979** | 7.50 | 73.3 | 67094 | 72 | 7123 | JAVRET000000000 | yes | 24 | 18 |
| **DSM 41859** | 7.30 | 73.3 | 101886 | 74 | 6762 | JAVREW000000000 | yes | 24 | 15 |
| **DSM 41982** | 7.07 | 73.4 | 110345 | 71 | 6477 | JAVRER000000000 | yes | 21 | 13 |
| **DSM 40473** | 7.66 | 71.1 | 250040 | 82 | 7110 | JAVRFI000000000 | yes | 44 | 14 |
| **DSM 41529** | 11.94 | 71.2 | 299431 | 79 | 11331 | JAVRFD000000000 | yes | 47 | 11 |
| **DSM 42041** | 6.46 | 72.8 | 181340 | 68 | 6152 | JAVREQ000000000 | yes | 29 | 16 |
| **DSM 41527** | 8.45 | 71.1 | 125653 | 78 | 7964 | JAVRFE000000000 | yes | 37 | 15 |
| **DSM 41528** | 9.07 | 71.7 | 173792 | 75 | 8651 | JAZBJP000000000 | yes | 35 | 8 |
| **DSM 41602** | 10.57 | 70.9 | 261044 | 72 | 9561 | JAZBJQ000000000 | yes | 49 | 14 |
| **DSM 41524** | 11.43 | 71.2 | 222260 | 75 | 10043 | JAZBJO000000000 | yes | 56 | 19 |
| ***Blastococcus* sp. DSM 46792** | 4.39 | 73.3 | 213299 | 55 | 4401 | JAVREI000000000 |  | 3 | 0 |
| ***Pseudonocardia* sp. DSM 45834** | 6.70 | 71.9 | 210630 | 51 | 6962 | JAVREJ000000000 | yes | 16 | 6 |
| ***Streptomonospora* sp. DSM 45055** | 5.59 | 72.6 | 179691 | 57 | 5192 | JAVREK000000000 | yes | 17 | 9 |
| ***Nocardiopsis* sp. DSM 44743** | 6.24 | 71.3 | 272499 | 75 | 5881 | JAVREP000000000 | yes | 15 | 3 |
| ***Jatrophihabitans* sp. DSM 44399** |  |  |  |  |  | NZ_JAVREH000000000.1 | yes | 16 | 9 |
| **Total** |  |  |  |  |  |  |  | 1022 | 418 |
